# Supplementary material for: Shifting cultivation and hunting across the savanna-forest mosaic in the Gran Sabana, Venezuela: facing changes
Source: PeerJ. 2021 Jun 17;9:e11612. doi: 10.7717/peerj.11612 (PMC8214850; doi:10.7717/peerj.11612)
Supplement: Supplemental Information 1 — Example model for Dasyprocta leporina. [file peerj-09-11612-s001.pdf]

Latent abundance mixture models for mammals and birds in La Gran Sabana, Venezuela

Ferrer-Paris, J.R.; Stachowicz, I; Sanchez-Mercado, A.

January 20, 2021

We want to test the response of mammal and bird species to the proximity of "conucos" in an area of the Gran Sabana in Venezuela using data from a pre-existing camera trap survey.

First we describe the general workflow for fitting the model for one species (*Dasyprocta leporina*) in four steps: (1) setup of analysis, (2) model fitting, (3) GoF, model criticism, and model selection, and (4) analysis of results.

Then we run this process for all target species in a batch and present the summary of result for all of them at the end of the document.

Model for *Dasyprocta leporina*

Setup of the analysis

We load up the R packages needed for the analysis and load the Rdata file with all the data.

```
# load packages
require(unmarked)
require(AICcmodavg)
require(chron)
require(raster)
require(cluster)
require(MuMIn)
require(reshape2)
require(dplyr)
require(ggplot2)
require(RColorBrewer)
# set working directory
setwd(work.dir)
# load Rdata file from repository
GIS.data <- sprintf("%s/Rdata/GIS.rda",script.dir)
load(GIS.data)
```

Sampling design and camera trap location

We used data from a previous camera trap survey conducted between September 2015 – April 2016. The original sampling design was developed to optimize covering habitat diversity in order to evaluate how mammal species richness is related to habitat types, and is described in detail in Stachowicz et al. (2020), but we provide here a brief summary of the initial setting and how we adapted data a posteriori for our analysis. Sampling design comprised six $50km^2$ blocks within the study area (B01 – B06) each one subdivided into 25 sampling units of $2km^2$.

The sampling universe included $N = 150$ sampling units, but only 30 cameras were available, thus sampling was divided into three periods of 60-days each, and in each period a two-levels stratified random sampling was used to select 30 sampling units (five in each block) for camera deployment. This stratification ensures a balanced representation of sampling units with different coverage of habitat types and fragmentation in each block during each period. As a side effect of this, some sampling units with unique values within each block (for example cells with high tree cover within a block dominated by savanna) were selected for sampling in two or three periods and those cameras were neither relocated nor replaced.

We filter the camera data and show their location in relation with the blocks 1 to 6 located in the eastern part of the Gran Sabana on the border of the Canaima National Park, circle size and colour is proportional to the time the camera was active (duration):

```
camaras %>% filter(bloque %in% sprintf("B%02i",1:6)) %>%
  mutate(bloque=droplevels(bloque),
    fecha1 = chron(dates.=as.character(fecha.act), times.=as.character(hora.act),
      format = c(dates = "y-m-d", times = "h:m:s")),
    fecha2 = chron(dates.=as.character(fecha.desact.real),
    times.=as.character(hora.desact.real),
      format = c(dates = "y-m-d", times = "h:m:s")),
    cdg = as.character(ID.original)) %>%
  group_by(cdg) %>%
  summarise(lat=mean(lat), lon=mean(lon), bloque=unique(bloque),
    hunting=unique(factor(caza.celda>0)), grp=unique(grp), H=mean(H), h=mean(h),
    tree_0500m=mean(tree_0500m), tree_1000m=mean(tree_1000m),
    tree_2500m=mean(tree_2500m), tree_5000m=mean(tree_5000m),
    drios=mean(drios), bsq=mean(buf.fragmen), ndvi=mean(ndvi.mu),
    fecha1=min(fecha1), fecha2=max(fecha2)) %>%
  mutate(duration=as.numeric(fecha2-fecha1)) ->
    cam.data
```

```
## `summarise()` ungrouping output (override with `.groups` argument)
```

We map the location of cameras in relation to the blocks

```
sampling.design <- subset(grd,cuadrado %in% 1:6)

map <- ggplot() +
  geom_polygon(data = sampling.design,
    aes(x = long, y = lat, group = group), colour = "black", fill = NA)
```

```
## Regions defined for each Polygons
```

```
map + theme_void() + coord_equal() +
  geom_point(data=cam.data,
    mapping=aes(x=lon,y=lat,size=duration,colour=duration))
```

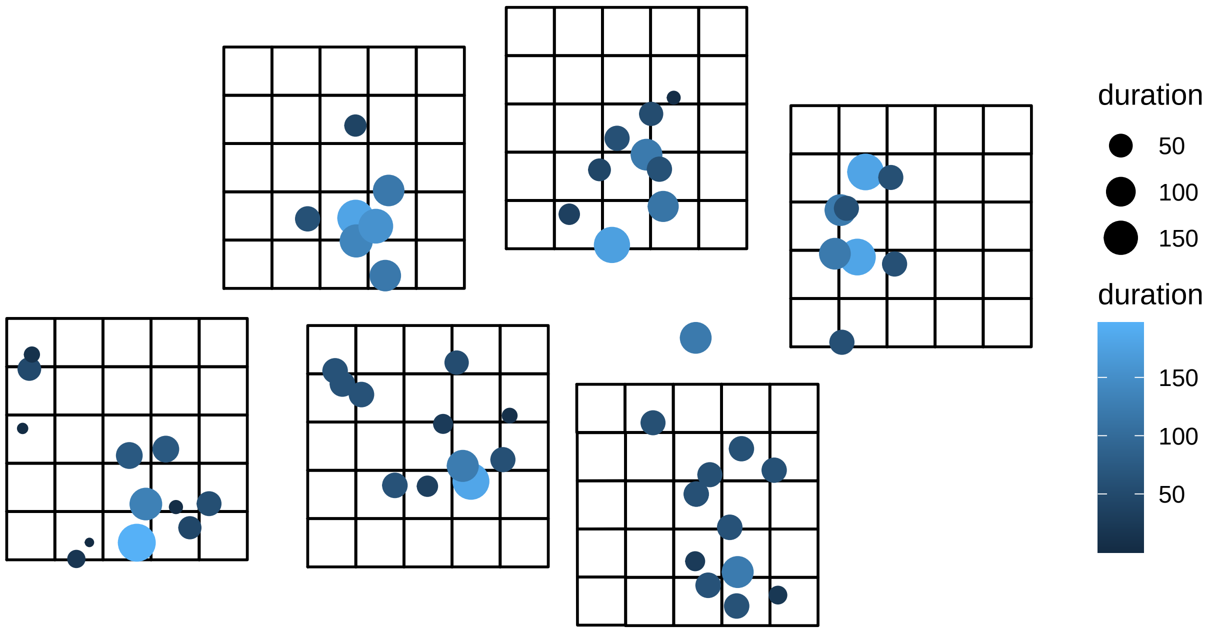

Vegetation / habitat types

Blocks were selected to represent landscapes with different values of total forest cover (*h*) and landscape fragmentation index (*H*; Stachowicz et al. 2020) :

```
cam.data %>% select(bloque,H,h) %>% unique()
```

```
## # A tibble: 6 x 3
##   bloque     H     h
##   <fct>  <dbl> <dbl>
## 1 B04     1.54  39.4
## 2 B02     1.64   5.4
## 3 B06     1.61  20.3
## 4 B05     1.5   53
## 5 B01     1.69   0.4
## 6 B03     1.62  82.5
```

Vegetation is dominated by scrub (*Clusia* spp. and *Gongylolepis* spp.), broadleaf grassland and savannas of Axonopus spp. and patches of gallery forest around the rivers, and evergreen montane surrounding the Ilú and Tramén tepuis massif. Sampling units were selected to represent the different habitat types present in each block:

```
cam.data %>% select(bloque,grp) %>% table()
```

```
##       grp
## bloque savanna shrub forest
##    B01      12     0      0
##    B02       6     3      0
```

```
##     B03         0       0       8
##     B04         1       1       5
##     B05         3       9       1
##     B06         4       5       2
```

Previous analysis suggested that most mammal species in the study area are associated with forest habitat and few are associated with shrubs or savanna (Stachowicz et al. 2020). We used mean tree cover (in percentage) as a quantitative variable correlated with these habitat types and consistent with metrics used for the sampling design. Mean tree cover was calculated from remote sensing products (Hansen et al. 2003) using a 1 km buffer around the camera location, the variable has a bimodal distribution with a lower mode at 10-20% corresponding with the savanna, a higher mode at 70-80% corresponding with forest and intermediate values roughly corresponding with the less common shrub habitat.

These groups have different values of tree cover and vegetation indices estimated from remote sensors. We tested buffers with different radii. A $1km$ radius is wide enough to represent the area of the most abundant game species home range (Cuniculus paca 2 - 3 ha, Jorge and Peres 2005; Dasyprocta leporina 3.4 - 1.6 ha, Benavides et al. 2017) and narrow enough to maintain variability in tree cover within the scale of a camera trapping site (Scotsont et al 2017). Increasing the radius implies more homogenous values and overlap between habitat types.

```
dat.m <- melt(cam.data,id.vars='grp', measure.vars=c('tree_0500m','tree_1000m','tree_2500m','tree_5000m

ggplot(dat.m) +
   geom_boxplot(aes(x=grp, y=value, color=variable)) +
   labs(title="Tree cover around the camera sites for each habitat type") +
   labs(y='% tree cover', x="habitats",  color='Buffer size',
      caption="Tree cover from Hansen et al. (2013)")
```

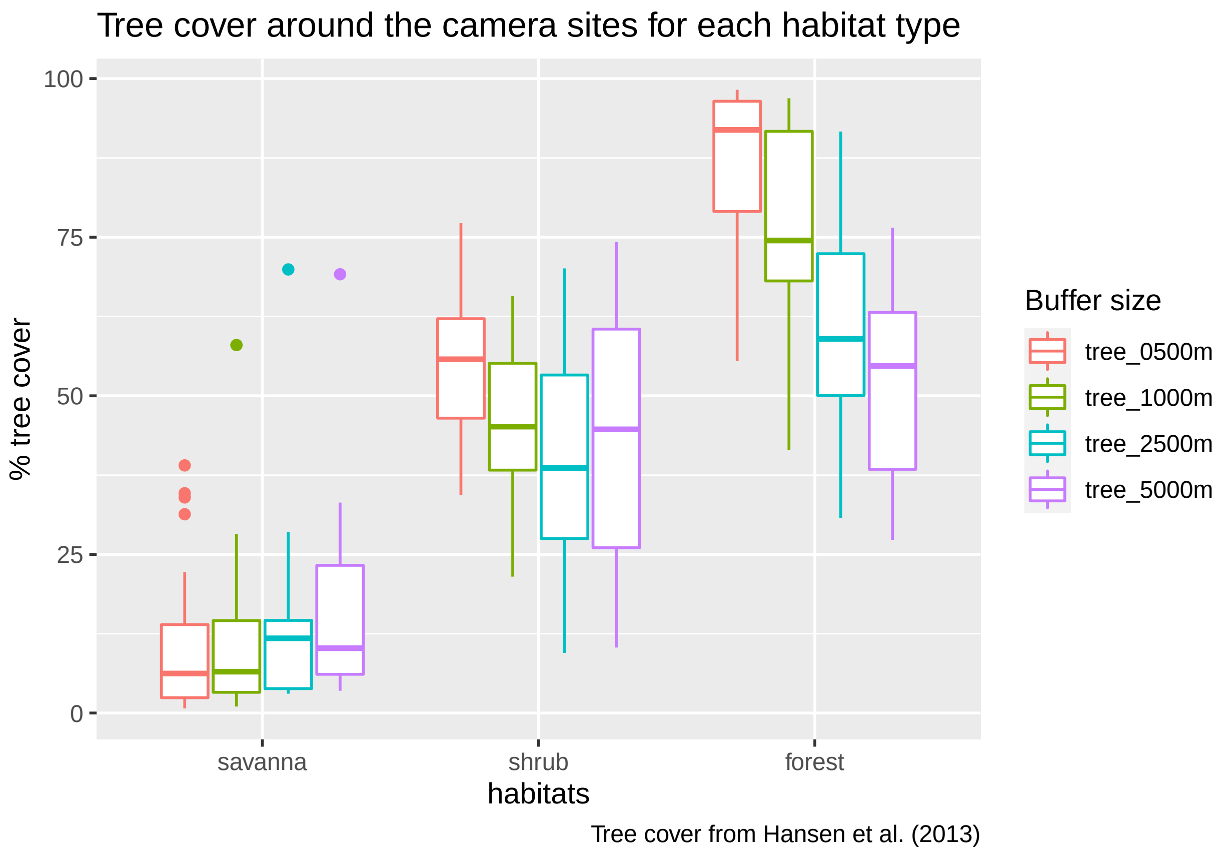

```r
ggplot(cam.data, aes(x=grp, y=ndvi)) +
    geom_boxplot(notch=F) + # or notch=T
    labs(title="NDVI around the camera sites for each habitat type (500m)") +
    labs(y='Normalized difference vegetation index', x="habitats",
      caption="NDVI derived from MODIS")
```

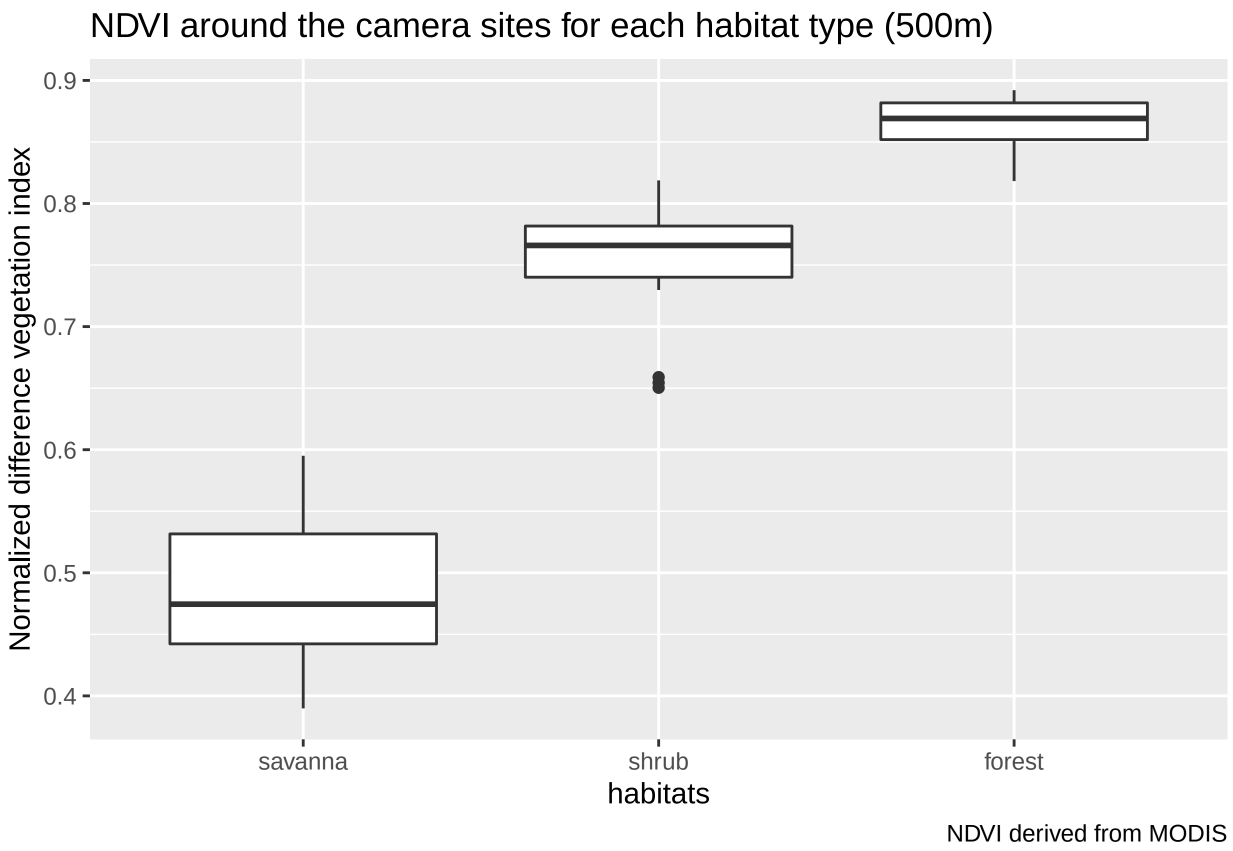

These two measures of vegetation are significantly correlated with landscape metrics for the blocks:

```r
cam.data %>% select(bsq,ndvi,H,h,tree_0500m,tree_1000m,tree_2500m,tree_5000m,drios) %>% cor()
```

```
##                   bsq       ndvi          H          h tree_0500m tree_1000m
## bsq         1.0000000  0.8309999 -0.3761795  0.6642176  0.8862003  0.8329604
## ndvi        0.8309999  1.0000000 -0.5309255  0.7490655  0.9607293  0.9088306
## H          -0.3761795 -0.5309255  1.0000000 -0.6004536 -0.4848918 -0.4907362
## h           0.6642176  0.7490655 -0.6004536  1.0000000  0.7434643  0.7838421
## tree_0500m  0.8862003  0.9607293 -0.4848918  0.7434643  1.0000000  0.9574364
## tree_1000m  0.8329604  0.9088306 -0.4907362  0.7838421  0.9574364  1.0000000
## tree_2500m  0.6915607  0.8282899 -0.6210522  0.8847185  0.8436329  0.9137586
## tree_5000m  0.5959857  0.7634414 -0.7159879  0.9030378  0.7555087  0.8130623
## drios      -0.1488141 -0.2985787  0.4527932 -0.1444651 -0.2375248 -0.2297796
##            tree_2500m tree_5000m      drios
## bsq         0.6915607  0.5959857 -0.1488141
## ndvi        0.8282899  0.7634414 -0.2985787
## H          -0.6210522 -0.7159879  0.4527932
## h           0.8847185  0.9030378 -0.1444651
## tree_0500m  0.8436329  0.7555087 -0.2375248
## tree_1000m  0.9137586  0.8130623 -0.2297796
```

```
## tree_2500m  1.0000000  0.9545729 -0.2311191
## tree_5000m  0.9545729  1.0000000 -0.2137828
## drios      -0.2311191 -0.2137828  1.0000000
```

```
with(cam.data,cor.test(bsq,H))
```

```
##
##  Pearson's product-moment correlation
##
## data:  bsq and H
## t = -3.092, df = 58, p-value = 0.003054
## alternative hypothesis: true correlation is not equal to 0
## 95 percent confidence interval:
##  -0.5751638 -0.1351660
## sample estimates:
##        cor
## -0.3761795
```

```
with(cam.data,cor.test(drios,tree_1000m ))
```

```
##
##  Pearson's product-moment correlation
##
## data:  drios and tree_1000m
## t = -1.7981, df = 58, p-value = 0.07737
## alternative hypothesis: true correlation is not equal to 0
## 95 percent confidence interval:
##  -0.45703763  0.02564122
## sample estimates:
##        cor
## -0.2297796
```

Influence of human populations / conucos

The Pemón are the only indigenous people inhabiting the Gran Sabana. There are four communities within the study area: Kawi (1100 m; - 61.243 W; 5.451 N; 50 people 2016), Mare-Paru (884 m; - 61.184 W; 5.594 N; 45 people in 2016), Uroy-Uaray (1,093 m; - 61.232 W; 5.442 N; 150 people in 2016) and Wuarapata (896 m; - 61.157; W 5.512 N; 50 people in 2016; information about the number of inhabitants was obtained from community leaders or capitanes).

We calculate the distance from each camera to the nearest community, blocks 1 and 5 are furthest away from the communities:

```
d1 <- pointDistance(cam.data[,c("lon","lat")],
  coordinates(comunidades), lonlat=T, allpairs=T)

cam.data$dcom <- apply(d1,1,min)

ggplot(cam.data, aes(x=dcom,fill=bloque)) +
  geom_histogram(binwidth=500, alpha = .8,col='black') +
  labs(title="Histogram for distance to communities") +
  labs(x="Distance (m)", y="Count")
```

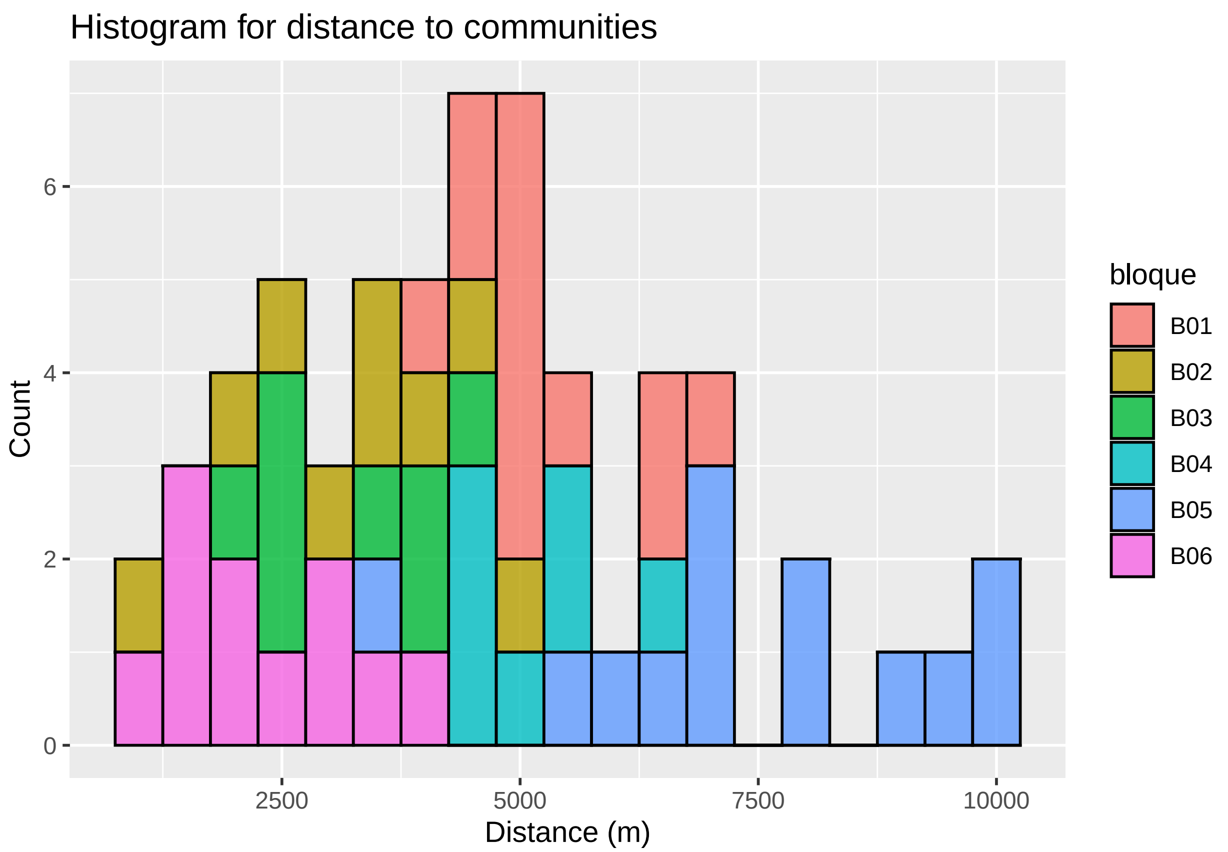

During fieldwork we marked with a GPS the location of active and recently abandoned conucos (n=25) identified in situ and hunting sites (n=32) reported by interviewees and confirmed by the local guides.

```
table(coordinates((conucos))[,1]>-61.3) #
```

```
##
## FALSE  TRUE
##    15    25
```

```
table(cam.data$hunting)
```

```
##
## FALSE  TRUE
##    26    34
```

Distance from each camera to the nearest conuco was calculated using the GPS coordinates from cameras and conucos. This variable had an asymmetric distribution with a mean value of 1.58 km and a range from 0 to 8 km. We also recorded which cameras were located adjacent or near reported hunting sites (binomial variable hunting, FALSE n = 23, TRUE n = 34). Notice that three camaras are located in savanna habitat more than 5km from the nearest conuco.

```
d1 <- pointDistance(cam.data[,c("lon","lat")],
   coordinates(conucos)[,1:2], lonlat=T, allpairs=T)

cam.data$dcon <- apply(d1,1,min)

ggplot(cam.data,aes(x=dcom,y=dcon,shape=grp,colour=hunting,size=duration)) +
geom_point() + labs(title="Distance to communities and conucos") +
labs(y="Distance to conucos (m)", x="Distance to communities (m)")
```

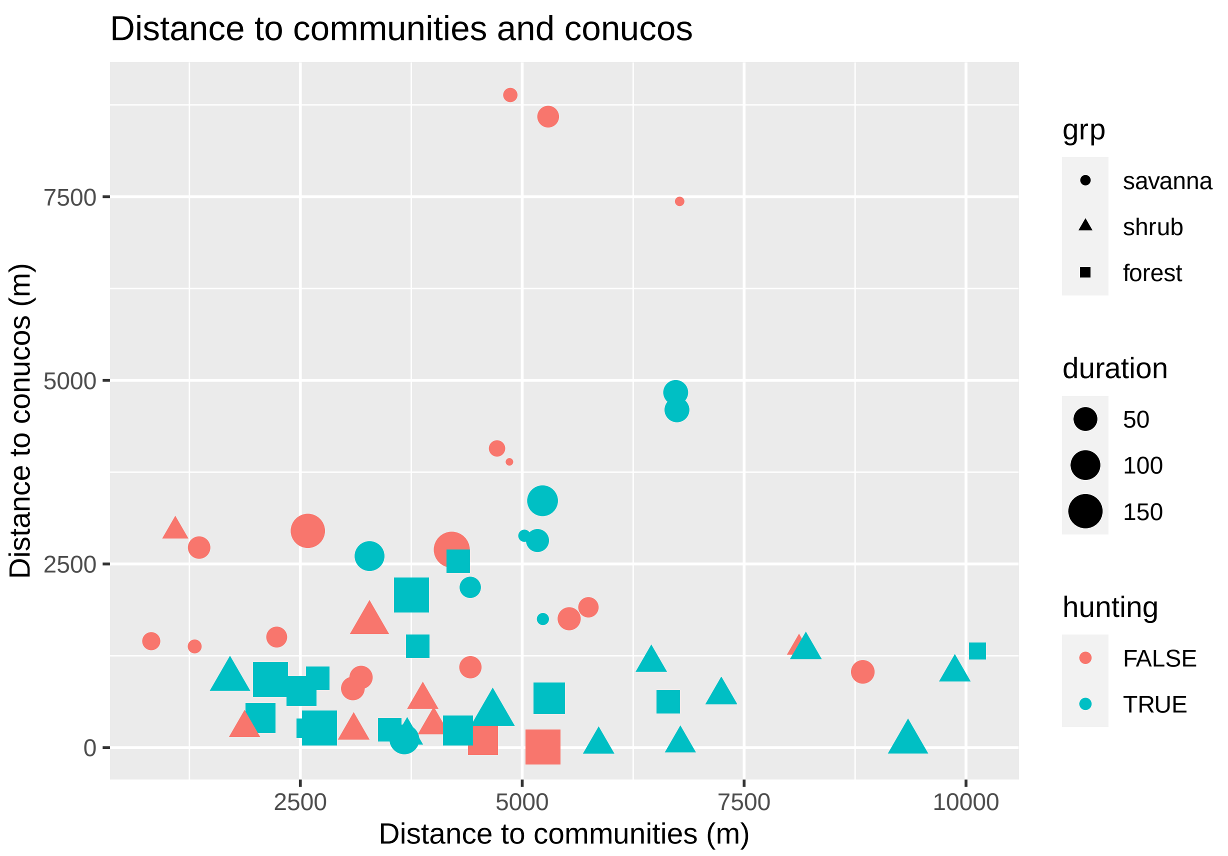

As an alternative measure of the influence of conuco, we calculate density as $g(u) = (\sum w[i])$ where the the weights are the inverse p-th powers of distance, $w[i] = 1/d(u, x[i])^p$ where $d(u, x[i])$ is the Euclidean distance from u to x[i].

```
p <- 0.25
w <- 1/((d1)^p)
cam.data$wcon <- apply(w,1,sum)

ggplot(cam.data,
  aes(y=wcon,x=dcon,colour=hunting,size=duration)) +
geom_point()
```

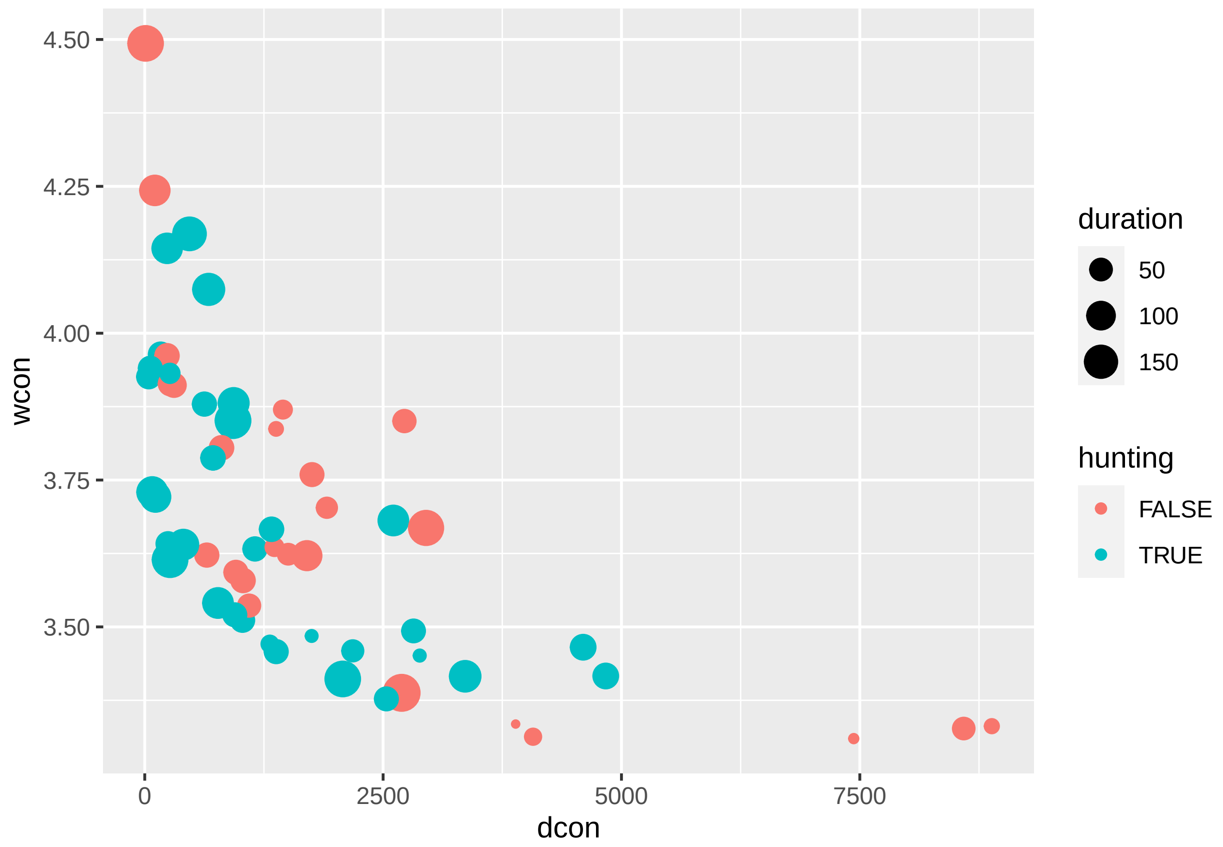

We check their correlation

```
cam.data %>% select(dcon,wcon,dcom) %>% cor()
```

```
##             dcon       wcon        dcom
## dcon  1.00000000 -0.6362453  0.08880916
## wcon -0.63624528  1.0000000 -0.17406593
## dcom  0.08880916 -0.1740659  1.00000000
```

```
# distance to conuco and density are neg. correlated
with(cam.data,cor.test(dcon,wcon))
```

```
##
##  Pearson's product-moment correlation
##
## data:  dcon and wcon
## t = -6.2807, df = 58, p-value = 4.674e-08
## alternative hypothesis: true correlation is not equal to 0
## 95 percent confidence interval:
##  -0.7663584 -0.4559895
## sample estimates:
##        cor
## -0.6362453
```

```
# distance to conuco and dist. to communities are not sig. correlated
 with(cam.data,cor.test(dcom,dcon))
```

```
##
##  Pearson's product-moment correlation
```

```
##
## data:  dcom and dcon
## t = 0.67903, df = 58, p-value = 0.4998
## alternative hypothesis: true correlation is not equal to 0
## 95 percent confidence interval:
##  -0.1689249  0.3351754
## sample estimates:
##        cor
## 0.08880916
```

Here we plot distance to nearest conuco/community against tree cover:

```r
cam.data$dhum <- with(cam.data,ifelse(dcon<dcom,dcon,dcom))

ggplot(data=cam.data, aes(y=dhum,x=tree_1000m,colour=hunting,size=duration)) +
geom_point() +
labs(title="Forest cover vs distance to nearest conuco/community") +
labs(y="Distance (m)", x="Forest cover (%)",colour="Hunting")
```

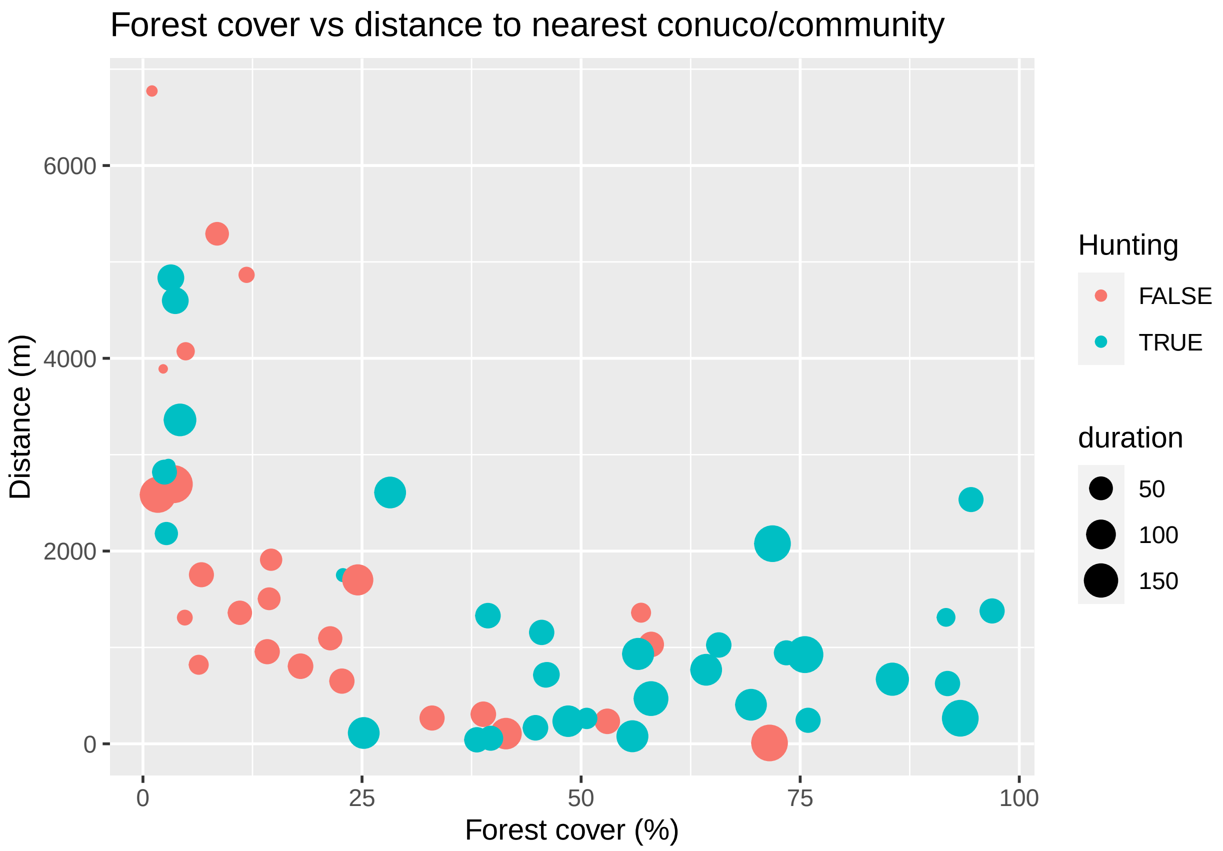


```r
##ggsave("Fig-bosque-caceria.pdf")
```

Signs of animal activity

We also recorded direct observations and indirect evidence (scats, tracks, scratches on trees, burrows, etc) of animal presence along the routes walked during field work, and recorded their coordinates with GPS. We had a total of n=159 records during 29 days of camera deployment and maintenance, with a mean of 16.8 km walked each day.

```r
table(subset(eventos,bloque %in% sprintf("B%02i",1:6))$camara %in% "RAS")
```

```
##
## FALSE  TRUE
##   920   159
```

```r
field.walk <- subset(track_points,coordinates(track_points)[,1]>-61.3)
field.xy <- spTransform(field.walk,crs("+proj=utm +zone=19n"))
walk.dist <- pointDistance(coordinates(field.xy)[-length(field.xy),], coordinates(field.xy)[-1,], lonla
walk.effort <- aggregate(walk.dist/1000,list(field.walk$time[-1]),sum)

summary(walk.effort$x)
```

```
##    Min. 1st Qu.  Median    Mean 3rd Qu.    Max.
##  0.6851 11.6386 16.9653 16.8187 24.2906 34.3734
```

We would expect that detection will be higher near areas of more animal activity. We calculate density of animal observations and tracks as $g(u) = (sum of w[i])$ where the the weights are the inverse p-th powers of distance, \$ w[i] = 1/d(u,x[i])^p\$ where d(u,x[i]) is the Euclidean distance from u to x[i].

```r
drastros <- pointDistance(subset(eventos,camara %in% "RAS")[,c("long","lat")], cam.data[,c("lon","lat")]

p <- 0.25
w <- 1/((drastros)^p)
cam.data$dras <- apply(w,2,sum)

ggplot(cam.data,aes(x=dras,fill=bloque)) +
geom_histogram(binwidth=.5, alpha = .8,col='black') +
labs(title="Histogram for density of animal tracks") +
labs(x="Density", y="Count")
```

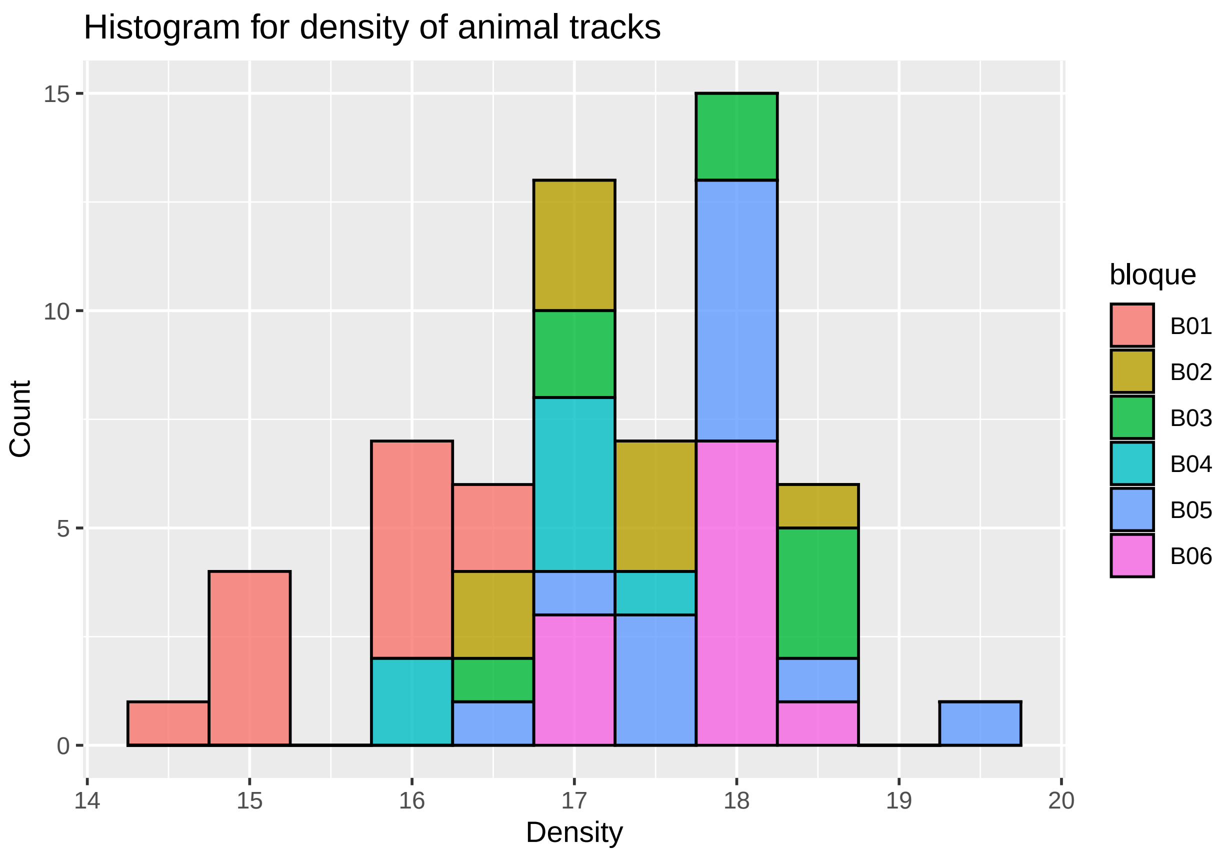

Summarizing detections from the camera trap survey

Total sampling effort was 4,548 cameras per day, resulting in 7,466 captures, which translated into 771 detection events for mammals and 226 detection events for birds across 86 sampling units (Table 2 in Stachowicz et al., 2020). We identified mammal species (Eisenberg, 1989; Linares, 1998) and birds (Hilty, Tudor & Gwynne, 2003) using reference works for Venezuela and South America. We can summarize the number of events, fotos and individuals for each species:

```
eventos %>% mutate(on.camera=!camara %in% "RAS") %>%
  group_by(species,on.camera) %>%
    summarise(events=n(),fotos=sum(fotos),inds=sum(number.of.animals))
```

```
## `summarise()` regrouping output by 'species' (override with `.groups` argument)
```

```
## # A tibble: 46 x 5
## # Groups:   species [29]
##    species      on.camera events fotos  inds
##    <fct>        <lgl>      <int> <int> <int>
##  1 C.alector    TRUE          48   777    84
##  2 C.olivaceus  TRUE           8    42     8
##  3 C.paca       FALSE          7     0     7
##  4 C.paca       TRUE         265  1916   268
##  5 C.thous      FALSE         32     0    32
##  6 C.thous      TRUE          44   246    49
##  7 C.unicinctus FALSE          2     0     2
##  8 C.unicinctus TRUE           2    33     2
##  9 D.imperfecta TRUE          14    51    14
```

```
## 10 D.kappleri   FALSE         18     0    18
## # ... with 36 more rows
```

We now filter the detection events registered on cameras for a single species (here *Dasyprocta leporina*):

```
eventos$cdg <- as.character(camaras$ID.original)[
  match(paste(eventos$bloque,eventos$periodo,eventos$camara),
    paste(camaras$bloque,camaras$period,camaras$camera))]

eventos %>% mutate(f1 = chron(dates.=sprintf("%s-%s-%s",ano,mes,dia),
    times.=as.character(hora.ini),
    format = c(dates = "y-m-d", times = "h:m:s")),
   f2 = chron(dates.=sprintf("%s-%s-%s",ano,mes,dia),
    times.=as.character(hora.ini),
    format = c(dates = "y-mon-d", times = "h:m:s"),
    out.format = c(dates = "y-m-d", times = "h:m:s"))) %>%
  transmute(cdg, camara, fotos, species=as.character(species), number.of.animals,
   fecha=chron(ifelse(is.na(f2),f1,f2),
   format = c(dates = "y-m-d", times = "h:m:s"))) %>%
  filter(cdg %in% cam.data$cdg & species %in% "D.leporina") ->
    event.data

event.data %>%
  mutate(on.camera=!camara %in% "RAS") %>%
    group_by(species,on.camera) %>%
      summarise(events=n(),fotos=sum(fotos),inds=sum(number.of.animals))
```

```
## `summarise()` regrouping output by 'species' (override with `.groups` argument)
```

```
## # A tibble: 1 x 5
## # Groups:   species [1]
##   species    on.camera events fotos  inds
##   <chr>      <lgl>      <int> <int> <int>
## 1 D.leporina TRUE         191  1423   192
```

To fit the model, we have to divide the continuous camera record (from camera activation to deactivation) into visits of fixed length. So we define a simple function to transform the list of events to a matrix:

```
make.obs.matrix <- function(x,y,w=NULL) {
  mtz <- matrix(0,nrow=length(unique(x$cdg)),
    ncol=length(y)-1,
      dimnames=list(unique(x$cdg),as.character(y)[-1]))
  for (k in 1:nrow(x)) {
     mtz[ x[k,"cdg"],] <-
      mtz[ x[k,"cdg"],] +
        table(cut(seq(x$fecha1[k],x$fecha2[k],by=1),
          breaks=y,label=as.character(y)[-1]))
  }
  if (!is.null(w)) {
    w %>%  mutate(sessions=cut(fecha,breaks=y,label=as.character(y)[-1])) -> z
    mtz[mtz==0] <- NA
    mtz <- mtz*0
    for (k in  seq(along=z$species)) {
       mtz[z[k,"cdg"],z[k,"sessions"]] <-
        mtz[z[k,"cdg"],z[k,"sessions"]] +
          z[k,"number.of.animals"]
    }
```

```
  }
  return(mtz)
}
```

We set a start date on the 21 september 2015, and select visit duration to create the matrix of sampling effort and observations. Initially we used a visit duration of one week, but this yields a large matrix with several zeros (no observations) and *NAs* (camera inactive):

```
ini <- chron(dates.="2015-09-21",times.="00:00:00",
  format = c(dates = "y-m-d", times = "h:m:s"))
visits <- ini + seq(from=7,by=7,length.out=28)

obs <- make.obs.matrix(data.frame(cam.data),visits,data.frame(event.data))
table(obs,useNA='always')
```

```
## obs
##    0    1    2    3    4    5    6    8    9   11 <NA>
##  598   62   25    7    2    1    3    1    1    1  919
```

We test larger visit duration to balance the number of zeros in the observation matrix, but this also reduces the number of detections. In fact we are "degrading" the input data since we are aggregating all detections per combination of site/visit, so multiple events get reduced to a single value of "1" (Kery and Royle 2016).

```
visits2 <- ini + seq(from=0,to=210,by=14)
visits3 <- ini + seq(from=0,to=210,by=21)
visits4 <- ini + seq(from=0,to=210,by=28)
clrs <- brewer.pal(3,'YlOrRd')

layout(matrix(1:4,ncol=4))
par(oma=c(2,2,0,0),mar=c(2,2,4,1))
obs <- make.obs.matrix(data.frame(cam.data),visits,data.frame(event.data))
image(1:ncol(obs),1:nrow(obs),t(obs>0),pty='m',col=clrs,xlab="",ylab="",main='7 days')
obs <- make.obs.matrix(data.frame(cam.data),visits2,data.frame(event.data))
image(1:ncol(obs),1:nrow(obs),t(obs>0),pty='m',col=clrs,xlab="",ylab="",main='14 days')
obs <- make.obs.matrix(data.frame(cam.data),visits3,data.frame(event.data))
image(1:ncol(obs),1:nrow(obs),t(obs>0),pty='m',col=clrs,xlab="",ylab="",main='21 days')
obs <- make.obs.matrix(data.frame(cam.data),visits4,data.frame(event.data))
image(1:ncol(obs),1:nrow(obs),t(obs>0),pty='m',col=clrs,xlab="",ylab="",main='28 days')

mtext('Visits',1,outer=T)
mtext('Sites (cameras)',2,outer=T)
```

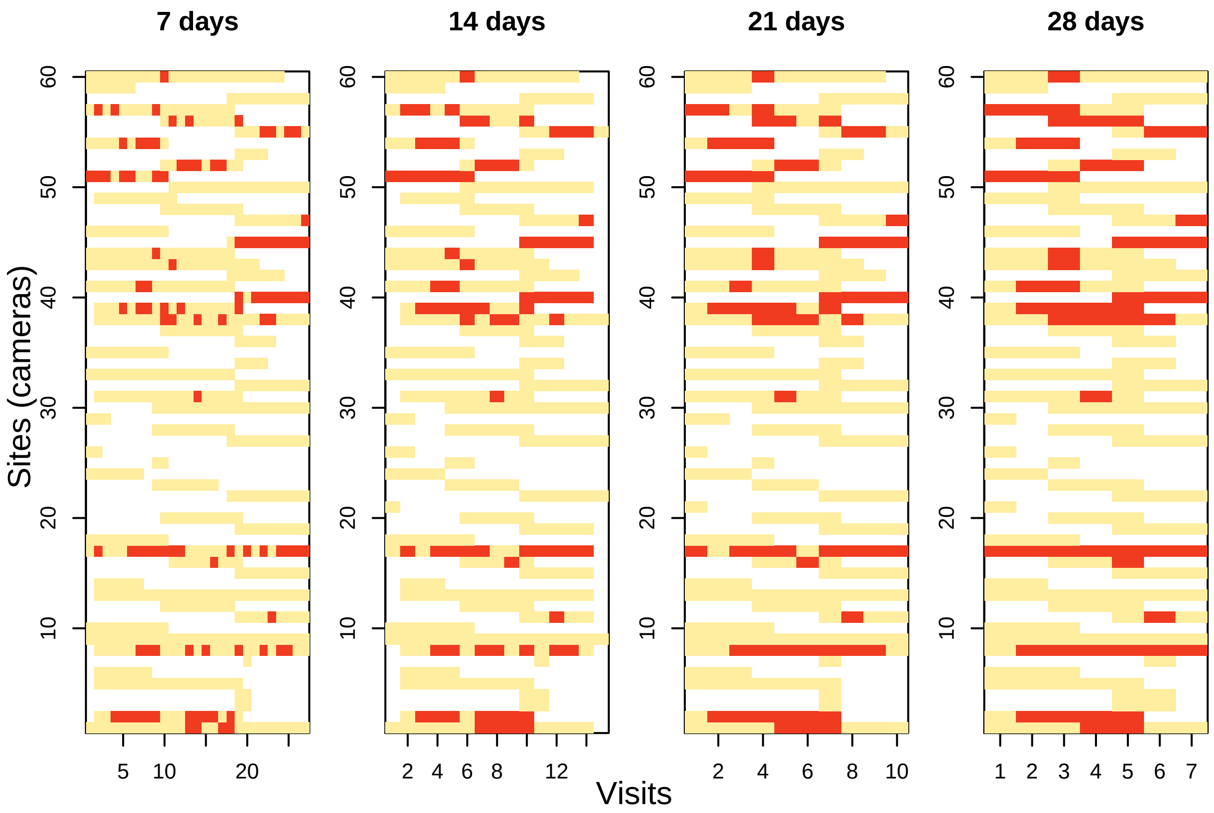

Model fitting

For each species we fit a hierarchical Bernoulli/Poisson N-mixture model (Royle–Nichols models, RN-models: Royle & Nichols, 2003) to evaluate how the probability of occupancy relates to forest cover and distance to conuco, allowing for abundance-induced heterogeneity in detection probability. RN-models are based on the assumption that the detection probability at a survey point p_{ij} depends on the species' site-specific abundance N_i:

$p_{ij} = 1 - (1 - r_{ij})N_i$

where r_{ij} is the detection probability of a single individual. Repeated visits at a survey point generate a history of detection/nondetection events y_{ij}, from which p_{ij} is estimated. The abundance state (N_i) of site i was modeled as $N_i\ Poison(\lambda_i)$, while the observation process was modeled as $y_{ij}/N_i\ Bernoulli(p_{ij})$. Estimation of p_{ij} allows us to draw conclusions about N_i.

In order to build detection histories for species recorded during the camera trap survey, we considered each camera location as a "site" (i; 57 in total). We divided the total sampling period of 180 days into several "visits" (j). Here we use a visit duration of 21 days.

Covariates of N_i (site covariates) and p_{ij} (observation covariates) were modeled using the logit link.

To test our hypothesis we need a measure of influence of conucos while controlling for the influence of habitat on species abundance, and the spatial and temporal heterogeneity in probability of detection.

We explored several covariates with alternative parameterizations to ensure best possible model fit given the restricted sample size and low number of detection for some species.

Formatting input

Now we arrange all data into a `unmarkedFrameOccu` object, we start with the detection history and observation covariates. Sampling date (date) was recorded as the number of days since the start of sampling (21 September 2015) to the beginning of the "visit" and rescaled between -1 and 1.

Sampling effort (effort) was calculated as the number of days the camera remained active divided by the duration of the visit. Thus, effort was always ≤ 1, and was set to empty value (NA) when the camera was not present or inoperative during the whole duration of the visit.

```
## Detection history
obs <- make.obs.matrix(data.frame(cam.data),visits3,data.frame(event.data))

## Observation covariates:
## sampling effort
sfrz <- make.obs.matrix(data.frame(cam.data),visits3)

## observation date
x <- seq(-1,1,length=ncol(obs))
obsDate <- matrix(rep(x,nrow(obs)),nrow=nrow(obs),byrow=T)
```

Now the site covariates. As site covariates we consider the following:

- Sampling design covariates: *block*, fragmentation index H and proportion of forest h
- Tree cover (*tree_xxxx*)percentage around the camera trap using different buffer sizes (500m, 1km, 2.5km and 5km)
- NDVI at the camera location (*ndvi*),
- Distance to nearest rivers (*drios*),
- Influence of conucos (*dcon*: distance to nearest or *wcon*: density of conucos)
- Influence of communities (distance to nearest, *dcom*),
- Influence of any human activity (distance to nearest community or conuco, *dhum*)
- Track density (*dras*; this one is used as a spatial covariate of detectability)

```
sC <- data.frame(cam.data[match(rownames(obs),cam.data$cdg),
   c("bloque","H","h","dcon","dcom","wcon",
      "dhum","tree_0500m","tree_1000m","tree_2500m","tree_5000m","drios","ndvi","grp","dras","hunting")]
sC$bloque <- droplevels(sC$bloque)
```

The continuous variables were standardized to zero mean and unit standard deviation:

```
for (k in c("H","h","dcon","dras","ndvi","tree_0500m","tree_1000m","tree_2500m","tree_5000m","drios","d
   sC[,k] <- (sC[,k]-mean(sC[,k]))/sd(sC[,k])
}
```

We select all camaras up to 5km from the conucos with total duration of at least one week

```
ss <- rownames(obs) %in% subset(cam.data,duration>7 & dcon<5000)$cdg
table(ss)
```

```
## ss
## FALSE  TRUE
##     6    54
```

Now everything together:

```
UMF <- unmarkedFrameOccu((obs[ss,]>0)+0,
  siteCovs=sC[ss,,drop=F],
  obsCovs=list(date=obsDate[ss,],sfrz=sfrz[ss,]/21))
```

Build the model: covariates of detection

First we check all possible combinations of covariates for detectability for a fixed null model of abundance:

```
fm00 <- occuRN(~ bloque+H+h+date+dras+sfrz ~ H+h+grp, UMF,K=50)
if (!exists("all.combs.1"))
   all.combs.1 <- dredge(fm00,fixed=c("lam(grp)","lam(h)","lam(H)"))
sw(all.combs.1)
```

```
##                        lam(grp) lam(h) lam(H) p(sfrz) p(H) p(h) p(date) p(dras)
## Sum of weights:        1.00     1.00   1.00   0.96    0.48 0.39 0.27    0.21
## N containing models:     64       64     64     32      32   32   32      32
##                        p(bloque)
## Sum of weights:        0.03
## N containing models:     32
```

This test suggest that *effort* (*p(sfrz)*) is the detectability covariate with most support, and there is negligible support to covariate *blocks*, but the combination of *H* or *h* has moderate support. We check if this holds with a different covariate of abundance:

```
 fm01 <- occuRN(~ bloque+H+h+date+dras+sfrz ~ tree_1000m, UMF,K=50)
if (!exists("all.combs.2"))
   all.combs.2 <- dredge(fm01,fixed=c("lam(tree_1000m)"))
  sw(all.combs.2)
```

```
##                        lam(tree_1000m) p(sfrz) p(dras) p(date) p(h) p(H)
## Sum of weights:        1.00            0.98    0.47    0.28    0.27 0.25
## N containing models:     64              32      32      32      32   32
##                        p(bloque)
## Sum of weights:        0.09
## N containing models:     32
```

... and found a similar result for *effort*, but better support for *dras* and *date*.

The best ranking models include those three variables:

```
subset(all.combs.2,delta < 2)
```

```
## Global model call: occuRN(formula = ~bloque + H + h + date + dras + sfrz ~ tree_1000m,
##     data = UMF, K = 50)
## ---
## Model selection table
##    p(Int) lam(Int) p(dat) p(drs)    p(h)   p(H) p(sfr) lam(tre_100) df   logLik
## 33 -2.623  -0.4762                                1.764        1.111  4 -106.134
## 37 -2.774  -0.4909        0.4887                  1.711        1.157  5 -105.056
## 39 -2.831  -0.4839 0.3158 0.6008                  1.779        1.125  6 -104.515
## 49 -2.514  -0.4921                         0.2225  1.746        1.099  5 -105.838
## 45 -2.635  -0.5783        0.5681 -0.2367          1.673        1.288  6 -104.644
##     AICc delta weight
## 33 221.1  0.00  0.327
## 37 221.4  0.28  0.285
## 39 222.8  1.73  0.138
## 49 222.9  1.84  0.130
## 45 223.1  1.99  0.121
## Models ranked by AICc(x)
```

Given these results, we selected sampling date, sampling effort (camera/day), and density of direct and indirect off-camera records to account for spatial and temporal heterogeneity in detectability (Cubaynes et al., 2010, REFs).

Build the model: covariates of abundance

In order to compare the covariates of abundance, we take a slightly different approach, given the high correlation between variables related to habitat type and vegetation. We compare alternative models avoiding duplicate variables:

```
mdls <- list(
   "H+h" = occuRN(~ date+sfrz+dras~ H+h, UMF,K=50),
   "H+h+habitat" = occuRN(~ date+sfrz+dras~ H+h+grp, UMF,K=50),
   "H+h+treecover 500m" = occuRN(~ date+sfrz+dras~ H+h+tree_0500m, UMF,K=50),
   "H+h+treecover 1k" = occuRN(~ date+sfrz+dras~ H+h+tree_1000m, UMF,K=50),
   "H+h+treecover 2,5k" = occuRN(~ date+sfrz+dras~ H+h+tree_2500m, UMF,K=50),
   "H+h+treecover 5k" = occuRN(~ date+sfrz+dras~ H+h+tree_5000m, UMF,K=50),
   "H+h+ndvi" = occuRN(~ date+sfrz+dras~ H+h+ndvi, UMF,K=50),
   "habitat" =  occuRN(~ date+sfrz+dras~ grp, UMF,K=50),
   "treecover 500m" = occuRN(~ date+sfrz+dras~ tree_0500m, UMF,K=50),
   "treecover 1k" = occuRN(~ date+sfrz+dras~ tree_1000m, UMF,K=50),
   "treecover 2.5k" = occuRN(~ date+sfrz+dras~ tree_2500m, UMF,K=50),
   "treecover 5k" = occuRN(~ date+sfrz+dras~ tree_5000m, UMF,K=50),
   "ndvi" = occuRN(~ date+sfrz+dras~ ndvi, UMF,K=50)
)

aictab(mdls,second.ord=T)
```

```
##
## Model selection based on AICc:
##
##                      K   AICc Delta_AICc AICcWt Cum.Wt      LL
## treecover 1k         6 222.82       0.00   0.62   0.62 -104.51
## ndvi                 6 225.19       2.38   0.19   0.81 -105.70
## treecover 500m       6 227.07       4.25   0.07   0.88 -106.64
## H+h+treecover 1k     8 228.21       5.39   0.04   0.93 -104.50
## treecover 2.5k       6 228.48       5.67   0.04   0.96 -107.35
## H+h+ndvi             8 230.57       7.75   0.01   0.98 -105.68
## habitat              7 230.80       7.99   0.01   0.99 -107.18
## H+h+treecover 500m 8 232.20       9.39   0.01   0.99 -106.50
## H+h+treecover 2,5k 8 232.46       9.64   0.01   1.00 -106.63
## H+h+habitat          9 235.14      12.33   0.00   1.00 -106.53
## treecover 5k         6 237.10      14.29   0.00   1.00 -111.66
## H+h                  7 239.49      16.67   0.00   1.00 -111.53
## H+h+treecover 5k     8 240.53      17.72   0.00   1.00 -110.67
```

Model with tree cover at 1km is slightly better than other alternatives.

Now rivers:

```
mdls <- list(
   "treecover 500m" = occuRN(~ date+sfrz+dras~ tree_0500m, UMF,K=50),
   "treecover 1k" = occuRN(~ date+sfrz+dras~ tree_1000m, UMF,K=50),
   "treecover 2.5k" = occuRN(~ date+sfrz+dras~ tree_2500m, UMF,K=50),
   "treecover 5k" = occuRN(~ date+sfrz+dras~ tree_5000m, UMF,K=50),
   "treecover 500m + rios" = occuRN(~ date+sfrz+dras~ tree_0500m+drios, UMF,K=50),
   "treecover 1k + rios" = occuRN(~ date+sfrz+dras~ tree_1000m+drios, UMF,K=50),
   "treecover 2.5k + rios" = occuRN(~ date+sfrz+dras~ tree_2500m+drios, UMF,K=50),
   "treecover 5k + rios" = occuRN(~ date+sfrz+dras~ tree_5000m+drios, UMF,K=50)
)
```

```r
aictab(mdls,second.ord=T)
```

```
##
## Model selection based on AICc:
##
##                         K   AICc Delta_AICc AICcWt Cum.Wt      LL
## treecover 1k            6 222.82       0.00   0.49   0.49 -104.51
## treecover 1k + rios     7 223.43       0.61   0.36   0.85 -103.50
## treecover 500m          6 227.07       4.25   0.06   0.90 -106.64
## treecover 500m + rios 7 227.13       4.31   0.06   0.96 -105.35
## treecover 2.5k          6 228.48       5.67   0.03   0.99 -107.35
## treecover 2.5k + rios 7 230.45       7.63   0.01   1.00 -107.01
## treecover 5k            6 237.10      14.29   0.00   1.00 -111.66
## treecover 5k + rios     7 239.42      16.60   0.00   1.00 -111.49
```

Rivers do not improve the fit (at least for this species) .

Models for all different measures of human influence (conuco density, distance, distance to communities and combined conuco/community distance) provide very similar results when using one variable at a time.

```r
fm11 <- occuRN(~ date+sfrz+dras~ tree_1000m+dcom, UMF,K=50)
fm12 <- occuRN(~ date+sfrz+dras~ tree_1000m+dcon, UMF,K=50)
fm13 <- occuRN(~ date+sfrz+dras~ tree_1000m+wcon, UMF,K=50)
fm14 <- occuRN(~ date+sfrz+dras~ tree_1000m+dhum, UMF,K=50)
fm15 <- occuRN(~ date+sfrz+dras~ tree_1000m+dcon+dcom, UMF,K=50)
fm16 <- occuRN(~ date+sfrz+dras~ tree_1000m+dcon+wcon, UMF,K=50)
fm17 <- occuRN(~ date+sfrz+dras~ tree_1000m+dcon*wcon, UMF,K=50)

aictab(list(fm11,fm12,fm13,fm14,fm15,fm16,fm17),
modnames=c("comunity","conuco (dist)","conuco (dens)","comunity/conuco combined","comunity + conuco","c
```

```
##
## Model selection based on AICc:
##
##                            K   AICc Delta_AICc AICcWt Cum.Wt      LL
## conuco (dist)              7 224.50       0.00   0.26   0.26 -104.03
## comunity/conuco combined 7 224.71       0.21   0.24   0.50 -104.14
## comunity                   7 225.39       0.89   0.17   0.67 -104.48
## conuco (dens)              7 225.43       0.93   0.16   0.83 -104.50
## conuco (dist+dens)         8 226.91       2.40   0.08   0.91 -103.85
## comunity + conuco          8 227.12       2.62   0.07   0.98 -103.96
## conuco (dist*dens)         9 229.71       5.21   0.02   1.00 -103.81
```

Distance to conuco is slightly better than other measures, but there is a strong correlation between them:

```r
cor.test(cam.data$dcon,cam.data$tree_1000m)
```

```
##
##  Pearson's product-moment correlation
##
## data:  cam.data$dcon and cam.data$tree_1000m
## t = -4.645, df = 58, p-value = 1.999e-05
## alternative hypothesis: true correlation is not equal to 0
## 95 percent confidence interval:
##  -0.6841717 -0.3074330
## sample estimates:
##        cor
```

```
## -0.5207087
```

Goodness of fit test

Finally we decided to fit a full model including the three observation covariates (p (date + effort + tracks_dens)) and the two site covariates (λ(tree_buffer + tree_buffer2 + dist_conuco)) using the occuRN function of the R package unmarked (Fiske & Chandler, 2011).

```
(fm01 <- occuRN(~ dras+sfrz+date ~ tree_1000m+dcon+drios, UMF,K=50))
```

```
##
## Call:
## occuRN(formula = ~dras + sfrz + date ~ tree_1000m + dcon + drios,
##     data = UMF, K = 50)
##
## Abundance:
##             Estimate    SE      z  P(>|z|)
## (Intercept)   -0.625 0.452 -1.381 1.67e-01
## tree_1000m     1.118 0.246  4.545 5.48e-06
## dcon          -0.299 0.564 -0.531 5.95e-01
## drios         -0.294 0.254 -1.161 2.46e-01
##
## Detection:
##             Estimate    SE     z  P(>|z|)
## (Intercept)   -2.862 0.621 -4.61 4.10e-06
## dras           0.485 0.360  1.35 1.78e-01
## sfrz           1.795 0.594  3.02 2.53e-03
## date           0.351 0.308  1.14 2.55e-01
##
## AIC: 222.7032
```

Since a couple of species might be associated with shrub habitat with intermediate values of tree cover (Stachowicz et al. 2020) it could be desirable to add a quadratic term for tree cover to the model.

```
(fm03 <- occuRN(~ dras+sfrz+date ~ tree_1000m+I(tree_1000m^2)+dcon+drios, UMF,K=50))
```

```
##
## Call:
## occuRN(formula = ~dras + sfrz + date ~ tree_1000m + I(tree_1000m^2) +
##     dcon + drios, data = UMF, K = 50)
##
## Abundance:
##                 Estimate    SE      z P(>|z|)
## (Intercept)      -0.6802 0.480 -1.418  0.1561
## tree_1000m        0.9756 0.477  2.046  0.0408
## I(tree_1000m^2)   0.0982 0.286  0.343  0.7315
## dcon             -0.3913 0.628 -0.623  0.5333
## drios            -0.2734 0.262 -1.043  0.2970
##
## Detection:
##             Estimate    SE     z  P(>|z|)
## (Intercept)   -2.875 0.622 -4.62 3.75e-06
## dras           0.513 0.368  1.39 1.63e-01
## sfrz           1.801 0.595  3.03 2.46e-03
## date           0.348 0.308  1.13 2.58e-01
##
```

```
## AIC: 224.5876
```

In this case the quadratic terms does not seem to be informative:

```r
AICc(fm01)
```

```
## [1] 225.9032
```

```r
AICc(fm03)
```

```
## [1] 228.6785
```

We assessed model fit for the full model using goodness of fit test based on Pearson χ^2 and parameter bootstrapping with 10,000 samples, and inspecting under- or overdispersion ($\hat{c}$, calculated by dividing the observed Chisq statistic by the mean of the statistics obtained from bootstrap samples), magnitude of parameter estimates and standard errors, and predicted values of the state variable at the sample locations (MacKenzie and Bailey 2004; Royle and Nichols 2016).

```r
nsim.val <- 100#00
if (!exists("ts01"))
  ts01 <- mb.gof.test(fm01,nsim=nsim.val,maxK=50,parallel = TRUE)
ts01
```

```
##
## MacKenzie and Bailey goodness-of-fit for Royle-Nichols occupancy model
##
## Pearson chi-square table:
##
##            Cohort Observed Expected Chi-square
## 0000000000      0        2     2.32       0.05
## 0000111000      0        1     0.01     166.43
## 0001110100      0        1     0.00     246.87
## 0011111110      0        1     0.03      33.25
## 1011101111      0        1     0.00     282.57
## 000100000.      1        1     0.02      63.06
## 00010000..      2        1     0.01      78.55
## 0000000...      3        2     3.28       0.50
## 0000100...      3        1     0.16       4.32
## 0001000...      3        1     0.15       4.82
## 0010000...      3        1     0.14       5.37
## 0111101...      3        1     0.04      25.37
## 0111111...      3        1     0.13       5.56
## 1101000...      3        1     0.01      82.50
## 0000......      4        5     4.08       0.21
## 0111......      4        1     0.14       5.09
## 1111......      4        1     0.06      14.66
## 000.......      5        3     2.44       0.13
## 00........      6        1     0.99       0.00
## ...0000000      7        2     1.64       0.08
## ...0000...      8        5     4.95       0.00
## ...0010...      8        1     0.55       0.37
## ...0110...      8        1     0.42       0.82
## ...1101...      8        1     0.05      18.29
## ...000....      9        1     0.96       0.00
## ......0000     10        6     5.79       0.01
## ......0001     10        1     0.18       3.83
## ......0100     10        1     0.59       0.29
## ......0110     10        1     0.78       0.07
```

```
## ......1111     10        2     0.51       4.33
## ......000.     11        1     0.93       0.01
## ......00..     12        3     1.78       0.83
## ......0...     13        1     0.97       0.00
##
## Chi-square statistic = 1068.102
## Number of bootstrap samples = 100
## P-value = 0.81
##
## Quantiles of bootstrapped statistics:
##    0%   25%   50%   75%  100%
##   221  1168  1538  2316 11146
##
## Estimate of c-hat = 0.5
```

For this species the model seem to be a good fit with no sign of over-dispersion. The estimate of $\hat{c}$ is well below 1, which might point to some degree of under-dispersion. I suspect this might be due to the heterogeneity in the sampling effort between cameras, with some cameras having very short periods with repeated visits. General practice is to ignore under-dispersion in model predictions (as long as it is not very extreme), and consider uncertainty estimates (standard deviation and confidence intervals) as rather conservative.

```r
(oms01 <- dredge(fm01,rank="AICc"))
```

```
## Fixed terms are "p(Int)" and "lam(Int)"

## Global model call: occuRN(formula = ~dras + sfrz + date ~ tree_1000m + dcon + drios,
##     data = UMF, K = 50)
## ---
## Model selection table
##     p(Int) lam(Int)  p(dat) p(dras) p(sfr) lam(dcn) lam(dris) lam(tre_100) df
## 53 -2.7660  -0.4698                  1.789          -0.356000        1.146  5
## 37 -2.6230  -0.4762                  1.764                           1.111  4
## 39 -2.7740  -0.4909          0.4887  1.711                           1.157  5
## 45 -2.5370  -0.7337                  1.698  -0.5853                  1.065  5
## 55 -2.8570  -0.4997          0.4130  1.740          -0.316600        1.183  6
## 40 -2.8310  -0.4839 0.31580  0.6008  1.779                           1.125  6
## 61 -2.6810  -0.6445                  1.742  -0.3819 -0.300300        1.109  6
## 54 -2.7930  -0.4608 0.20860          1.845          -0.370700        1.122  6
## 47 -2.6860  -0.6783          0.3958  1.676  -0.4287                  1.114  6
## 38 -2.6330  -0.4710 0.16730          1.803                           1.089  5
## 56 -2.9240  -0.4961 0.33540  0.5260  1.820          -0.330300        1.153  7
## 46 -2.5470  -0.7530 0.24090          1.746  -0.6530                  1.030  6
## 63 -2.8030  -0.6025          0.3759  1.718  -0.2365 -0.287800        1.156  7
## 48 -2.7410  -0.6959 0.34740  0.5053  1.746  -0.4913                  1.074  7
## 62 -2.6990  -0.6649 0.24660          1.796  -0.4503 -0.307100        1.075  7
## 64 -2.8620  -0.6247 0.35080  0.4846  1.795  -0.2993 -0.294300        1.118  8
## 35 -1.3880  -0.5050          0.5406                                  1.133  4
## 41 -1.1190  -0.8184                         -0.6993                  1.035  4
## 33 -1.1440  -0.5084                                                  1.073  3
## 49 -1.2420  -0.5116                                 -0.318900        1.105  4
## 51 -1.4290  -0.5213          0.4751                 -0.275800        1.157  5
## 43 -1.3130  -0.7437          0.4242         -0.5241                  1.091  5
## 36 -1.3870  -0.4987 0.20690  0.6144                                  1.108  5
## 57 -1.1920  -0.7606                         -0.5512 -0.240000        1.064  5
## 42 -1.0970  -0.8335 0.15270                 -0.7465                  1.010  5
## 34 -1.1310  -0.5073 0.07635                                          1.061  4
```

```
## 52 -1.4260  -0.5174 0.21610  0.5496                  -0.281700        1.131  6
## 50 -1.2270  -0.5098 0.10080                          -0.324400        1.090  5
## 59 -1.3710  -0.6912          0.4105         -0.3797 -0.229900        1.120  6
## 44 -1.3090  -0.7573 0.24220  0.5002         -0.5740                  1.058  6
## 58 -1.1690  -0.7776 0.15130                 -0.5997 -0.240900        1.039  6
## 60 -1.3640  -0.7087 0.23920  0.4847         -0.4319 -0.229900        1.087  7
## 13 -2.1930  -0.6529                  1.679  -1.2570                         4
## 14 -2.2330  -0.6648 0.45220          1.755  -1.2900                         5
## 16 -2.3990  -0.6476 0.56760  0.4575  1.789  -1.2240                         6
## 15 -2.2840  -0.6381          0.2717  1.684  -1.2080                         5
## 29 -2.1870  -0.6548                  1.678  -1.2680  0.028130               5
## 30 -2.2310  -0.6656 0.45180          1.754  -1.2940  0.008449               6
## 32 -2.3980  -0.6481 0.56730  0.4576  1.789  -1.2260  0.005726               7
## 31 -2.2790  -0.6400          0.2724  1.682  -1.2200  0.030460               6
## 9  -0.7933  -0.7200                         -1.2920                         3
## 8  -2.4780  -0.1716 0.55480  0.7226  1.830                                  5
## 10 -0.7754  -0.7307 0.35150                 -1.3230                         4
## 7  -2.3770  -0.1645          0.5551  1.723                                  4
## 5  -2.1580  -0.2068                  1.722                                  3
## 11 -0.8858  -0.7031          0.2832         -1.2410                         4
## 24 -2.5010  -0.1990 0.58730  0.6513  1.857          -0.282000               6
## 6  -2.1820  -0.2172 0.40240          1.795                                  4
## 12 -0.9110  -0.7101 0.44900  0.4339         -1.2570                         5
## 21 -2.2240  -0.2157                  1.739          -0.288200               4
## 25 -0.7848  -0.7234                         -1.3160  0.060450               4
## 22 -2.2670  -0.2216 0.44600          1.822          -0.323400               5
## 23 -2.3910  -0.1908          0.4880  1.740          -0.244100               5
## 26 -0.7688  -0.7339 0.34980                 -1.3410  0.045180               5
## 27 -0.8789  -0.7054          0.2853         -1.2640  0.065160               5
## 28 -0.9060  -0.7125 0.44710  0.4347         -1.2760  0.046080               6
## 3  -0.9512  -0.2117          0.5682                                         3
## 4  -0.9637  -0.2138 0.43700  0.7027                                         4
## 1  -0.7186  -0.2644                                                         2
## 17 -0.7591  -0.2775                                 -0.258300               3
## 2  -0.6910  -0.2718 0.30060                                                 3
## 20 -0.9577  -0.2439 0.46140  0.6415                 -0.240400               5
## 19 -0.9447  -0.2394          0.5108                 -0.207700               4
## 18 -0.7382  -0.2825 0.33530                         -0.286700               4
##      logLik  AICc delta weight
## 53 -104.894 221.0  0.00  0.139
## 37 -106.134 221.1  0.05  0.136
## 39 -105.056 221.4  0.32  0.118
## 45 -105.328 221.9  0.87  0.090
## 55 -104.114 222.0  0.98  0.085
## 40 -104.515 222.8  1.78  0.057
## 61 -104.624 223.0  2.00  0.051
## 54 -104.633 223.1  2.01  0.051
## 47 -104.685 223.2  2.12  0.048
## 38 -105.968 223.2  2.15  0.047
## 56 -103.497 223.4  2.39  0.042
## 46 -104.991 223.8  2.73  0.035
## 63 -104.021 224.5  3.44  0.025
## 48 -104.035 224.5  3.47  0.025
## 62 -104.267 225.0  3.93  0.019
```

```
## 64 -103.352 225.9  4.87  0.012
## 35 -110.039 228.9  7.86  0.003
## 41 -110.247 229.3  8.27  0.002
## 33 -111.416 229.3  8.28  0.002
## 49 -110.377 229.6  8.53  0.002
## 51 -109.296 229.8  8.80  0.002
## 43 -109.482 230.2  9.18  0.001
## 36 -109.754 230.8  9.72  0.001
## 57 -109.792 230.8  9.80  0.001
## 42 -110.084 231.4 10.38  0.001
## 34 -111.375 231.6 10.53  0.001
## 52 -108.981 231.7 10.71  0.001
## 50 -110.303 231.9 10.82  0.001
## 59 -109.052 231.9 10.85  0.001
## 44 -109.095 232.0 10.94  0.001
## 58 -109.629 233.0 12.01  0.000
## 60 -108.670 233.8 12.74  0.000
## 13 -114.814 238.4 17.41  0.000
## 14 -113.714 238.7 17.64  0.000
## 16 -112.949 239.7 18.65  0.000
## 15 -114.515 240.3 19.24  0.000
## 29 -114.809 240.9 19.83  0.000
## 30 -113.714 241.2 20.18  0.000
## 32 -112.949 242.3 21.29  0.000
## 31 -114.510 242.8 21.77  0.000
## 9  -119.478 245.4 24.40  0.000
## 8  -117.296 245.8 24.80  0.000
## 10 -118.690 246.2 25.16  0.000
## 7  -118.787 246.4 25.35  0.000
## 5  -120.023 246.5 25.49  0.000
## 11 -119.143 247.1 26.07  0.000
## 24 -116.659 247.1 26.07  0.000
## 6  -119.177 247.2 26.13  0.000
## 12 -117.970 247.2 26.15  0.000
## 21 -119.307 247.4 26.39  0.000
## 25 -119.457 247.7 26.69  0.000
## 22 -118.268 247.8 26.75  0.000
## 23 -118.319 247.9 26.85  0.000
## 26 -118.678 248.6 27.57  0.000
## 27 -119.119 249.5 28.45  0.000
## 28 -117.958 249.7 28.67  0.000
## 3  -123.467 253.4 32.38  0.000
## 4  -122.367 253.5 32.51  0.000
## 1  -124.809 253.9 32.82  0.000
## 17 -124.237 255.0 33.92  0.000
## 2  -124.255 255.0 33.95  0.000
## 20 -121.913 255.1 34.04  0.000
## 19 -123.134 255.1 34.05  0.000
## 18 -123.545 255.9 34.87  0.000
## Models ranked by AICc(x)
```

```
sw(oms01)
```

```
##                      lam(tree_1000m) p(sfrz) lam(drios) p(dras) lam(dcon)
## Sum of weights:      1.00            0.98    0.43       0.42    0.31
```

```
## N containing models:   32              32      32          32      32
##                      p(date)
## Sum of weights:      0.29
## N containing models:   32
```

```
mavg01 <- model.avg(oms01, subset = delta < 10,fit=T)
summary(mavg01)
```

```
##
## Call:
## model.avg(object = get.models(object = oms01, subset = delta <
##     10))
##
## Component model call:
## occuRN(formula = ~<24 unique rhs>, data = UMF, K = 50)
##
## Component models:
##        df  logLik   AICc delta weight
## 236     5 -104.89 221.04  0.00   0.14
## 36      4 -106.13 221.09  0.05   0.14
## 356     5 -105.06 221.36  0.32   0.12
## 136     5 -105.33 221.91  0.87   0.09
## 2356    6 -104.11 222.01  0.98   0.09
## 3456    6 -104.51 222.82  1.78   0.06
## 1236    6 -104.62 223.04  2.00   0.05
## 2346    6 -104.63 223.05  2.01   0.05
## 1356    6 -104.69 223.16  2.12   0.05
## 346     5 -105.97 223.19  2.15   0.05
## 23456   7 -103.50 223.43  2.39   0.04
## 1346    6 -104.99 223.77  2.73   0.04
## 12356   7 -104.02 224.48  3.44   0.02
## 13456   7 -104.03 224.50  3.47   0.02
## 12346   7 -104.27 224.97  3.93   0.02
## 123456  8 -103.35 225.90  4.87   0.01
## 35      4 -110.04 228.89  7.86   0.00
## 13      4 -110.25 229.31  8.27   0.00
## 3       3 -111.42 229.31  8.28   0.00
## 23      4 -110.38 229.57  8.53   0.00
## 235     5 -109.30 229.84  8.80   0.00
## 135     5 -109.48 230.21  9.18   0.00
## 345     5 -109.75 230.76  9.72   0.00
## 123     5 -109.79 230.83  9.80   0.00
##
## Term codes:
##       lam(dcon)      lam(drios) lam(tree_1000m)        p(date)         p(dras)
##               1               2               3              4               5
##         p(sfrz)
##               6
##
## Model-averaged coefficients:
## (full average)
##                  Estimate Std. Error z value Pr(>|z|)
## lam(Int)         -0.54672    0.39949   1.369  0.17114
## lam(drios)       -0.14321    0.22857   0.627  0.53096
## lam(tree_1000m)   1.12224    0.23507   4.774 1.80e-06 ***
```

```
## p(Int)          -2.70254    0.63155   4.279 1.88e-05 ***
## p(sfrz)          1.73029    0.61870   2.797  0.00516 **
## p(dras)          0.20053    0.32625   0.615  0.53878
## lam(dcon)       -0.14991    0.37141   0.404  0.68648
## p(date)          0.07737    0.20506   0.377  0.70596
##
## (conditional average)
##                 Estimate Std. Error z value Pr(>|z|)
## lam(Int)         -0.5467     0.3995   1.369  0.17114
## lam(drios)       -0.3321     0.2417   1.374  0.16953
## lam(tree_1000m)   1.1222     0.2351   4.774 1.80e-06 ***
## p(Int)           -2.7025     0.6315   4.279 1.88e-05 ***
## p(sfrz)           1.7556     0.5865   2.993  0.00276 **
## p(dras)           0.4762     0.3485   1.366  0.17185
## lam(dcon)        -0.4806     0.5323   0.903  0.36658
## p(date)           0.2656     0.3072   0.865  0.38730
## ---
## Signif. codes:  0 '***' 0.001 '**' 0.01 '*' 0.05 '.' 0.1 ' ' 1
```

```
cbind(coef(mavg01),confint(mavg01))
```

```
##                                2.5 %     97.5 %
## lam(Int)        -0.5467156 -1.3297005  0.2362694
## lam(drios)      -0.3320656 -0.8058405  0.1417092
## lam(tree_1000m)  1.1222445  0.6615172  1.5829718
## p(Int)          -2.7025430 -3.9403503 -1.4647356
## p(sfrz)          1.7555938  0.6061061  2.9050816
## p(dras)          0.4762068 -0.2069286  1.1593423
## lam(dcon)       -0.4805777 -1.5237834  0.5626280
## p(date)          0.2655562 -0.3364831  0.8675955
```

```
prd <-  predict(mavg01,type='state')
ggplot(data=UMF@siteCovs,aes(x=tree_1000m,y=dcon,size=prd$fit,colour=prd$se.fit)) +
geom_point()
```

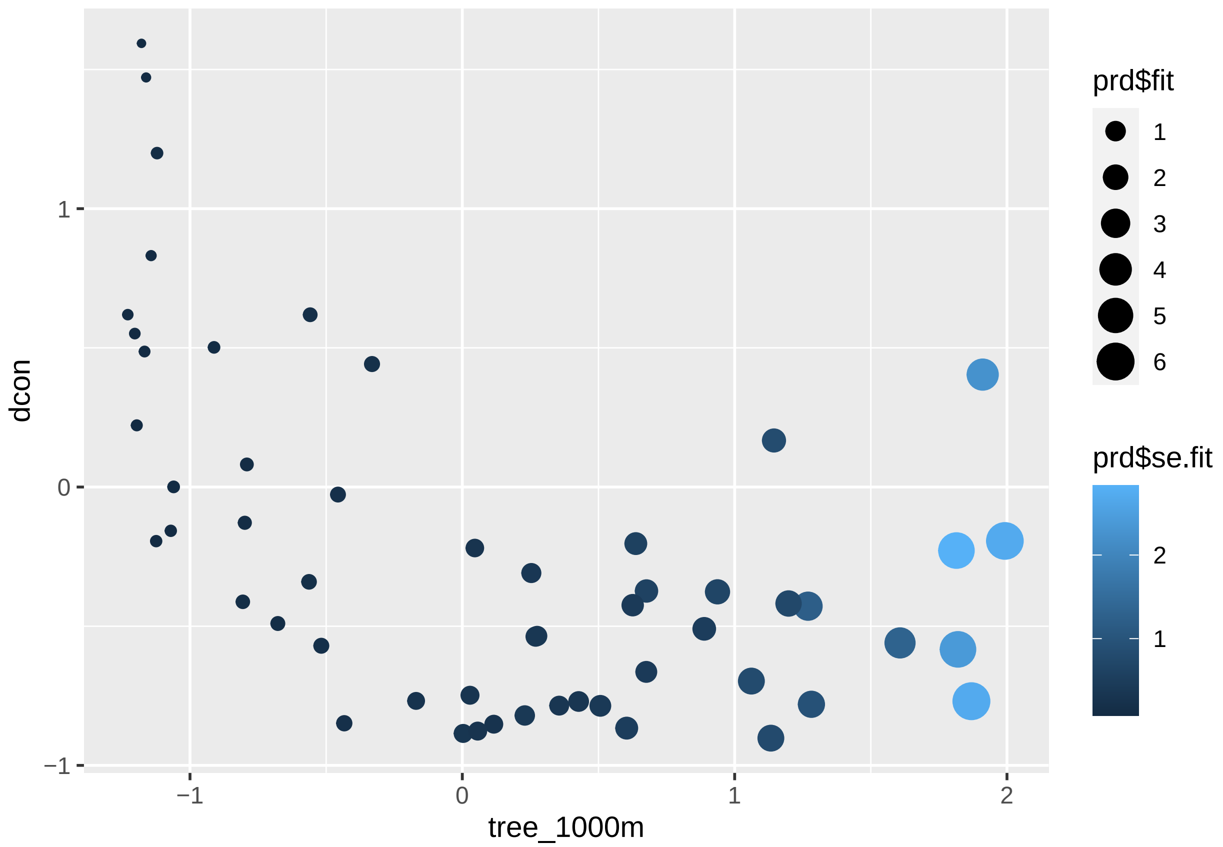

For species with a suitable full model, we proceeded to create a model selection table with all combinations of covariates (32 models for species with linear effect of tree cover and 48 for species with quadratic effect of tree cover), ranked models according to information criteria corrected for small sample size (AICc if c1or QAICc if c>1), and the corresponding delta(Q)AICc and model weights.

We assessed the relative importance of each detection and occupancy covariate by calculating the sum of weights of the model containing that variable (Burnham and Anderson 2002; Symonds and Moussalli 2011). Values range from zero to one indicating increasing levels of support, and we use an informal scale to describe the level of support as very strong (>0.9), strong (0.6 - 0.9), moderate (0.3 - 0.6) and low (<0.3). We further calculated model averaged coefficients and predictions of the state variable (λ) based on the subset of models with $\delta(Q)AICc \leq 10$ (Burnham et al 2011; Mazerolle 2020). In case of overdispersion (c>1) we assumed the lack of fit is due to unaccounted sources of error and used the value of c to inflate the standard errors and confidence intervals. For underdispersed models (c1), no modification to standard errors or intervals was made, but consider these as conservative assessments of uncertainty (Kery and Royle 2019).

Selection of hunting localities

To assess hunting behaviour of the Pemon we evaluated both the interview responses on vegetation type and season with contingency tables, and the spatial information on reported hunting sites with a logistic regression. We tabulated the number of interview responses from each community for the three levels of preferred hunting vegetation types (forest, savanna and mixed) and the two levels of hunting seasons (dry, and rainy season). We used the (Chi-square) test to assess the significance of the relationship between variables. For the logistic regression we used the data collected during field work at 57 sites with cameras and fitted a logistic regression to the binomial hunting variable with formula: Eq 5 $logit(hunting)\ \beta_0 + \beta_1 tree_buffer + \beta_2 dist_conuco$

notes on the occu RN model

Kery and Royle 2016:

> The RN model may be useful to estimate abundance from replicated measurements of presence/absence, to accommodate detection heterogeneity when focusing on occupancy (Dorazio,2007) or to link occupancy data with abundance data in an integrated model (Conroy et al., 2008)
>
> In practice, the RN model has not been extensively used to actually estimate abundance. Arguably, it is more sensitive to parametric assumptions and it will fail when abundance and/or detection are high. Moreover, adopting mixture distributions other than the Poisson, such as the NB, leads to unstable estimates and often also to biologically unreasonably high estimates for abundanceHence, the RN model may be most useful as an occupancy model that accounts for part of the site-specific heterogeneity in detection (Dorazio, 2007) or in integrated models to link occupancy data with data that are directly informative about abundance (Conroy et al., 2008). Note also that Yamaura et al. (2011) have used it as the basis for their community models (Chapter 11).
