# Supplementary material for: Shifting cultivation and hunting across the savanna-forest mosaic in the Gran Sabana, Venezuela: facing changes
Source: PeerJ. 2021 Jun 17;9:e11612. doi: 10.7717/peerj.11612 (PMC8214850; doi:10.7717/peerj.11612)
Supplement: Supplemental Information 2 [file peerj-09-11612-s002.pdf]

Test of garden hunting hypothesis for mammals in La Gran Sabana, Venezuela using occupancy models

Stachowicz, I; Ferrer-Paris, J.R.; Sanchez-Mercado, A. (in prep)

December 2, 2020

```
## `summarise()` ungrouping output (override with `.groups` argument)
```

Results of latent abundance models for 29 species detected in camera trap surveys

We attempted modelling the abundance of 29 species detected during the camera trap survey. These species were detected in at least two different occasions:

```
eventos %>% group_by(species) %>% summarise(nr.events=n(),nr.fotos=sum(fotos),max.nr.individuals=max(nu
```

```
## `summarise()` ungrouping output (override with `.groups` argument)

##           species nr.events nr.fotos max.nr.individuals
## 1   D.marsupialis         2        6                  1
## 2        L.wiedii         2        7                  1
## 3        T.pecari         2        9                  1
## 4     C.unicinctus        4       33                  1
## 5        P.tajacu         4       15                  2
## 6    O.virginianus        5       38                  1
## 7   T.tetradactyla        6       33                  2
## 8      C.olivaceus        8       42                  1
## 9          N.nasua        8       54                  7
## 10      P.jacquacu        8       63                  1
## 11       P.maximus        9       60                  1
## 12 H.hydrochaeris        10      192                  3
## 13    D.imperfecta       14       51                  1
## 14      L.pardalis       18       99                  1
## 15      P.concolor       18       82                  1
## 16          P.onca       18       73                  1
## 17       E.barbara       21      166                  1
## 18    M.tridactyla       21      152                  1
## 19         T.major       24      112                  2
## 20     M.americana       30      263                  1
## 21    T.terrestris       33      186                  2
## 22 D.novemcinctus        38     1181                  1
## 23       C.alector       48      777                  4
## 24      D.kappleri       52      248                  1
## 25   M.gouazoubira       66      712                  2
## 26     L.rufaxilla       68      385                  2
## 27         C.thous       76      246                  2
## 28      D.leporina      194     1423                  2
```

```
## 29        C.paca       272     1916                    2
```

Here one event was defined as a sequence of consecutive photographs from a single camera. For most species each event recorded a single individual, but in some species pairs or small groups could be capture in a single event.

The RN model uses data from detection history matrix, where each row represents a "site" (camera location) and each column represents a time unit or "visit". This means we need to divide the period of camera activity into time units of fixed duration. Each entry in the matrix consist of a 0 for non-detection or a 1 for detection (or empty values if the camera was not active during a giving time unit).

This format of detection histories does do not use information on the number of indivuals per detection event, or number of independent detections events per time units (for example two events in following days within a time unit count as a single detection).

Thus the effective number of detections for modeling species anundance will depend on how these events are distributed among different cameras and time units.

Species with few effective detections

Fitted models for species with only two effective detections among the 54 camara traps selected for the analysis showed clear signs of lack of fit: * MacKenzie and Bailey Goodness-of-fit Test with p-values <0.05, * estimate of c-hat (overdispersion) » 1 * large or very large values in coefficients estimates

```
tbl1 %>% filter(n.detect<5) %>% select(1:5)
```

```
##          species n.detect chi.square p.value   c.hat.est
## 1   C.unicinctus        2 164.850705  0.0212  6.3495930
## 2  D.marsupialis        2  14.869533  0.3859  0.9202898
## 3 H.hydrochaeris        2  16.880230  0.0684  4.5178344
## 4        L.wiedii        2  27.117515  0.3138  1.1922936
## 5  O.virginianus        2  29.868537  0.2262  1.4201888
## 6        P.tajacu        2  47.969248  0.0062 14.4928694
## 7        T.pecari        2   7.803391  0.4498  0.9334348
```

Species with 5 to 10 effective detections among the 54 camara traps performed better on the Goodness of fit test, but still had problems with very large or unrealistic values in coefficients estimates, and were also discarded.

```
tbl1 %>% filter(n.detect>=5 & n.detect <10) %>% select(1:5)
```

```
##          species n.detect chi.square p.value c.hat.est
## 1    C.olivaceus        7  268.68752  0.1137 1.9639307
## 2        N.nasua        5  154.40128  0.2162 1.2860354
## 3      P.concolor        9  184.96443  0.1000 1.8972678
## 4      P.jacquacu        6  130.10821  0.3483 1.0667008
## 5       P.maximus        6   63.55353  0.6537 0.3748963
## 6    T.terrestris        8  164.60607  0.5368 0.5499967
## 7 T.tetradactyla        5  128.92489  0.2194 1.1707677
```

So we focus the analysis on 15 species with at least 11 effective detections.

Results for each species

D.imperfecta

No sign of lack of fit, c-hat values less than 1

```
spp <- "D.imperfecta"
mod <- ifelse(spp %in% with.quad.term,"03","01")
```

```r
tbl1 %>% filter(species %in% spp) %>% select(1:5)
```

```
##        species n.detect chi.square p.value c.hat.est
## 1 D.imperfecta       11   292.1195  0.4206  0.570783
```

Most support for variables:

```r
sw(get(sprintf("oms%s.%s",mod,spp)))
```

```
##                      p(dras) p(sfrz) lam(tree_1000m) lam(dcon) lam(drios)
## Sum of weights:      0.96    0.45    0.38            0.24      0.24
## N containing models:   32      32      32              32        32
##                      p(date)
## Sum of weights:      0.23
## N containing models:   32
```

Summary of model averaging estimates (use conditional average):

```r
summary(get(sprintf("mavg%s.%s",mod,spp)))
```

```
##
## Call:
## model.avg(object = get.models(object = oms01, subset = delta <
##     10))
##
## Component model call:
## occuRN(formula = ~<48 unique rhs>, data = UMF, K = 50)
##
## Component models:
##        df logLik  AICc delta weight
## 5       3 -35.76 78.00  0.00   0.14
## 56      4 -34.71 78.23  0.23   0.13
## 35      4 -35.03 78.87  0.88   0.09
## 356     5 -34.06 79.38  1.38   0.07
## 25      4 -35.69 80.20  2.20   0.05
## 15      4 -35.75 80.32  2.32   0.04
## 45      4 -35.75 80.32  2.33   0.04
## 256     5 -34.64 80.53  2.53   0.04
## 156     5 -34.70 80.66  2.66   0.04
## 456     5 -34.70 80.66  2.66   0.04
## 135     5 -34.89 81.02  3.03   0.03
## 235     5 -35.00 81.24  3.25   0.03
## 345     5 -35.03 81.30  3.31   0.03
## 1356    6 -33.92 81.62  3.62   0.02
## 2356    6 -34.03 81.84  3.85   0.02
## 3456    6 -34.06 81.91  3.92   0.02
## 125     5 -35.66 82.58  4.58   0.01
## 245     5 -35.69 82.63  4.63   0.01
## 145     5 -35.75 82.74  4.74   0.01
## 1256    6 -34.62 83.02  5.03   0.01
## 2456    6 -34.64 83.07  5.07   0.01
## 1456    6 -34.70 83.19  5.19   0.01
## 1235    6 -34.88 83.55  5.55   0.01
## 1345    6 -34.89 83.56  5.56   0.01
## 2345    6 -34.99 83.78  5.78   0.01
## 12356   7 -33.91 84.26  6.26   0.01
```

```
## 13456   7 -33.91 84.26  6.27   0.01
## (Null)  2 -40.02 84.27  6.28   0.01
## 23456   7 -34.03 84.49  6.50   0.01
## 6       3 -39.04 84.56  6.56   0.01
## 3       3 -39.27 85.02  7.02   0.00
## 1245    6 -35.66 85.11  7.12   0.00
## 36      4 -38.31 85.44  7.44   0.00
## 12456   7 -34.62 85.67  7.67   0.00
## 12345   7 -34.88 86.20  8.20   0.00
## 4       3 -39.91 86.30  8.30   0.00
## 1       3 -39.92 86.32  8.32   0.00
## 2       3 -40.02 86.52  8.52   0.00
## 46      4 -38.91 86.63  8.63   0.00
## 16      4 -38.95 86.71  8.72   0.00
## 26      4 -39.03 86.88  8.89   0.00
## 123456  8 -33.91 87.02  9.02   0.00
## 34      4 -39.13 87.07  9.07   0.00
## 13      4 -39.26 87.35  9.35   0.00
## 23      4 -39.27 87.35  9.36   0.00
## 346     5 -38.14 87.54  9.54   0.00
## 136     5 -38.30 87.86  9.86   0.00
## 236     5 -38.31 87.87  9.87   0.00
##
## Term codes:
##       lam(dcon)      lam(drios) lam(tree_1000m)         p(date)         p(dras)
##               1               2               3               4               5
##         p(sfrz)
##               6
##
## Model-averaged coefficients:
## (full average)
##                  Estimate Std. Error z value Pr(>|z|)
## lam(Int)        -1.017970   0.706150   1.442   0.1494
## p(Int)          -3.774553   1.529065   2.469   0.0136 *
## p(dras)          1.403994   0.589514   2.382   0.0172 *
## p(sfrz)          0.858206   1.370132   0.626   0.5311
## lam(tree_1000m)  0.213528   0.406591   0.525   0.5995
## lam(drios)       0.039905   0.271496   0.147   0.8831
## lam(dcon)        0.031842   0.480174   0.066   0.9471
## p(date)          0.007073   0.360610   0.020   0.9844
##
## (conditional average)
##                 Estimate Std. Error z value Pr(>|z|)
## lam(Int)        -1.01797    0.70615   1.442  0.14942
## p(Int)          -3.77455    1.52906   2.469  0.01357 *
## p(dras)          1.46005    0.52873   2.761  0.00575 **
## p(sfrz)          1.90910    1.47301   1.296  0.19496
## lam(tree_1000m)  0.56882    0.48815   1.165  0.24392
## lam(drios)       0.17160    0.54256   0.316  0.75179
## lam(dcon)        0.13633    0.98637   0.138  0.89007
## p(date)          0.03153    0.76087   0.041  0.96695
## ---
## Signif. codes:  0 '***' 0.001 '**' 0.01 '*' 0.05 '.' 0.1 ' ' 1
```

```r
ss <- match(rownames(get(sprintf("UMF.%s",spp))@y),cam.data$cdg)
prd <- predict(get(sprintf("mavg%s.%s",mod,spp)),type='state')
dtf <- data.frame(fit=prd$fit, se.fit=prd$se.fit, hunting=cam.data[ss,"hunting"], dcon=cam.data[ss,"dcon
ggplot(dtf, aes(y=dcon, x=tree_1000m,size=fit,colour=se.fit)) +
    geom_point() + ylab("Distance to conucos  (m)") + xlab("Tree cover (% in 1km buffer)") +
    labs(title=spp,size='Predicted values',colour='Prediction S.E.')
```

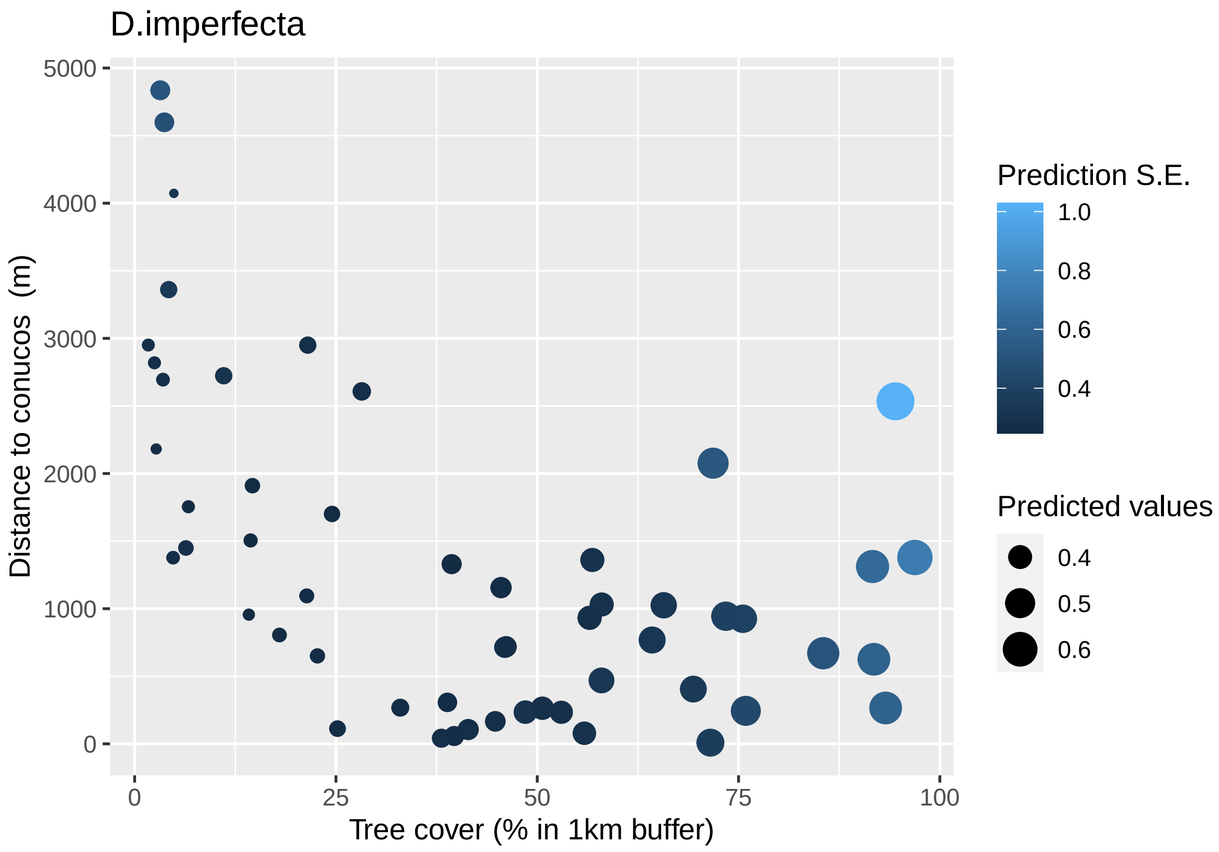

P.onca

No sign of lack of fit, c-hat values less than 1

```r
spp <- "P.onca"
mod <- ifelse(spp %in% with.quad.term,"03","01")

 tbl1 %>% filter(species %in% spp) %>% select(1:5)
```

```
##   species n.detect chi.square p.value c.hat.est
## 1  P.onca       12   427.2777    0.35 0.8463562
```

Most support for variables:

```r
sw(get(sprintf("oms%s.%s",mod,spp)))
```

```
##                      lam(tree_1000m) lam(drios) lam(dcon) p(dras) p(date)
## Sum of weights:      0.68            0.30       0.26      0.25    0.24
## N containing models:   32              32         32        32      32
##                      p(sfrz)
## Sum of weights:      0.23
```

```
## N containing models:   32
```

Summary of model averaging estimates (use conditional average):

```
summary(get(sprintf("mavg%s.%s",mod,spp)))
```

```
##
## Call:
## model.avg(object = get.models(object = oms01, subset = delta <
##     10))
##
## Component model call:
## occuRN(formula = ~<61 unique rhs>, data = UMF, K = 50)
##
## Component models:
##        df logLik   AICc delta weight
## 3       3 -46.01  98.49  0.00   0.16
## (Null)  2 -47.95 100.13  1.64   0.07
## 23      4 -45.69 100.20  1.71   0.07
## 13      4 -45.92 100.66  2.17   0.05
## 36      4 -45.98 100.78  2.29   0.05
## 34      4 -46.00 100.81  2.32   0.05
## 35      4 -46.01 100.83  2.34   0.05
## 2       3 -47.72 101.92  3.43   0.03
## 1       3 -47.80 102.07  3.58   0.03
## 4       3 -47.91 102.30  3.81   0.02
## 235     5 -45.52 102.30  3.81   0.02
## 6       3 -47.93 102.35  3.86   0.02
## 5       3 -47.94 102.37  3.88   0.02
## 135     5 -45.62 102.49  4.00   0.02
## 236     5 -45.67 102.58  4.09   0.02
## 123     5 -45.69 102.64  4.15   0.02
## 234     5 -45.69 102.64  4.15   0.02
## 136     5 -45.90 103.05  4.56   0.02
## 12      4 -47.13 103.07  4.58   0.02
## 134     5 -45.92 103.10  4.61   0.02
## 346     5 -45.98 103.20  4.71   0.01
## 356     5 -45.98 103.21  4.72   0.01
## 345     5 -46.00 103.24  4.75   0.01
## 25      4 -47.43 103.68  5.19   0.01
## 26      4 -47.70 104.22  5.73   0.01
## 24      4 -47.71 104.24  5.75   0.01
## 2345    6 -45.24 104.27  5.78   0.01
## 14      4 -47.73 104.28  5.79   0.01
## 15      4 -47.77 104.36  5.87   0.01
## 16      4 -47.78 104.38  5.89   0.01
## 1345    6 -45.37 104.53  6.04   0.01
## 1235    6 -45.38 104.54  6.05   0.01
## 45      4 -47.88 104.57  6.08   0.01
## 46      4 -47.90 104.62  6.13   0.01
## 56      4 -47.93 104.68  6.19   0.01
## 2356    6 -45.50 104.79  6.30   0.01
## 1356    6 -45.61 105.01  6.52   0.01
## 1236    6 -45.66 105.12  6.63   0.01
## 2346    6 -45.67 105.12  6.63   0.01
```

```
## 1234    6 -45.69 105.17  6.68   0.01
## 124     5 -47.09 105.42  6.93   0.00
## 126     5 -47.10 105.46  6.97   0.00
## 125     5 -47.13 105.50  7.01   0.00
## 245     5 -47.13 105.52  7.03   0.00
## 1346    6 -45.90 105.59  7.10   0.00
## 3456    6 -45.98 105.74  7.25   0.00
## 256     5 -47.42 106.09  7.59   0.00
## 246     5 -47.69 106.64  8.15   0.00
## 12345   7 -45.11 106.66  8.17   0.00
## 146     5 -47.73 106.70  8.21   0.00
## 156     5 -47.76 106.77  8.28   0.00
## 23456   7 -45.24 106.91  8.42   0.00
## 456     5 -47.88 107.00  8.51   0.00
## 145     5 -47.88 107.00  8.51   0.00
## 12356   7 -45.36 107.15  8.66   0.00
## 13456   7 -45.37 107.17  8.68   0.00
## 12346   7 -45.66 107.76  9.27   0.00
## 1245    6 -47.01 107.80  9.31   0.00
## 1246    6 -47.07 107.93  9.44   0.00
## 1256    6 -47.10 107.99  9.50   0.00
## 2456    6 -47.13 108.05  9.56   0.00
##
## Term codes:
##       lam(dcon)      lam(drios) lam(tree_1000m)         p(date)         p(dras)
##               1               2               3               4               5
##         p(sfrz)
##               6
##
## Model-averaged coefficients:
## (full average)
##                  Estimate Std. Error z value Pr(>|z|)
## lam(Int)        -0.99764    0.79362   1.257   0.2087
## lam(tree_1000m)  0.47426    0.45323   1.046   0.2954
## p(Int)          -2.20945    0.87014   2.539   0.0111 *
## lam(drios)       0.10587    0.27774   0.381   0.7031
## lam(dcon)        0.01726    0.43754   0.039   0.9685
## p(sfrz)         -0.04615    0.49514   0.093   0.9257
## p(date)          0.03551    0.29318   0.121   0.9036
## p(dras)          0.18217    0.70451   0.259   0.7960
##
## (conditional average)
##                  Estimate Std. Error z value Pr(>|z|)
## lam(Int)        -0.99764    0.79362   1.257   0.2087
## lam(tree_1000m)  0.70196    0.37974   1.849   0.0645 .
## p(Int)          -2.20945    0.87014   2.539   0.0111 *
## lam(drios)       0.35070    0.41191   0.851   0.3945
## lam(dcon)        0.06549    0.85053   0.077   0.9386
## p(sfrz)         -0.19815    1.01123   0.196   0.8447
## p(date)          0.14956    0.58732   0.255   0.7990
## p(dras)          0.71939    1.25442   0.573   0.5663
## ---
## Signif. codes:  0 '***' 0.001 '**' 0.01 '*' 0.05 '.' 0.1 ' ' 1
```

```r
ss <- match(rownames(get(sprintf("UMF.%s",spp))@y),cam.data$cdg)
prd <- predict(get(sprintf("mavg%s.%s",mod,spp)),type='state')
dtf <- data.frame(fit=prd$fit, se.fit=prd$se.fit, hunting=cam.data[ss,"hunting"], dcon=cam.data[ss,"dcon
ggplot(dtf, aes(y=dcon, x=tree_1000m,size=fit,colour=se.fit)) +
    geom_point() + ylab("Distance to conucos  (m)") + xlab("Tree cover (% in 1km buffer)") +
    labs(title=spp,size='Predicted values',colour='Prediction S.E.')
```

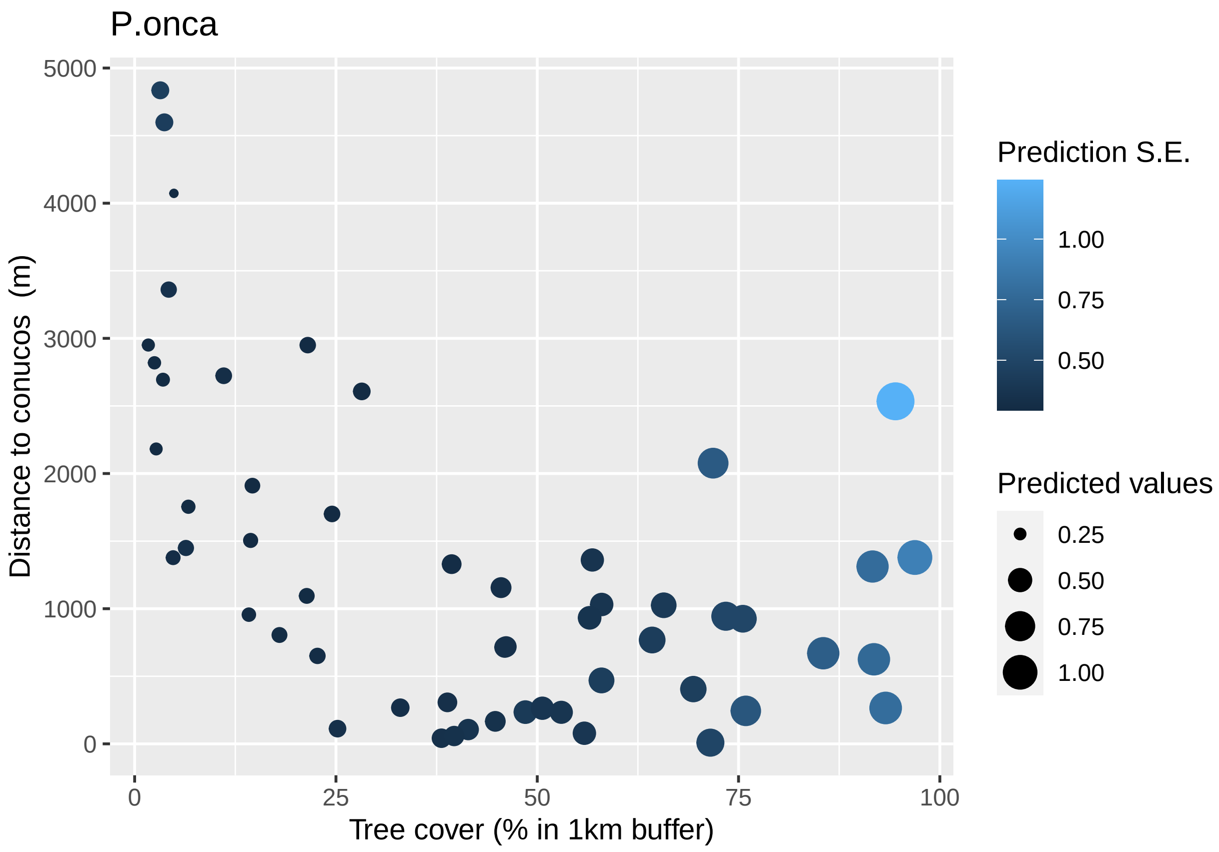

M.tridactyla

No sign of lack of fit, c-hat values less than 1

```r
spp <- "M.tridactyla"
mod <- ifelse(spp %in% with.quad.term,"03","01")

 tbl1 %>% filter(species %in% spp) %>% select(1:5)
```

```
##        species n.detect chi.square p.value c.hat.est
## 1 M.tridactyla       13   413.1652  0.3151 0.8339423
```

Most support for variables:

```r
sw(get(sprintf("oms%s.%s",mod,spp)))
```

```
##                      lam(drios) p(dras) p(sfrz) lam(dcon) lam(tree_1000m)
## Sum of weights:      0.89       0.60    0.48    0.38      0.35
## N containing models:   32         32      32      32        32
##                      p(date)
## Sum of weights:      0.23
```

```
## N containing models:   32
```

Summary of model averaging estimates (use conditional average):

```r
summary(get(sprintf("mavg%s.%s",mod,spp)))
```

```
##
## Call:
## model.avg(object = get.models(object = oms01, subset = delta <
##     10))
##
## Component model call:
## occuRN(formula = ~<61 unique rhs>, data = UMF, K = 50)
##
## Component models:
##         df logLik   AICc delta weight
## 25       4 -47.79 104.41  0.00   0.09
## 256      5 -46.61 104.47  0.06   0.09
## 125      5 -46.93 105.11  0.70   0.06
## 1256     6 -45.67 105.12  0.72   0.06
## 235      5 -47.02 105.30  0.89   0.06
## 2356     6 -45.83 105.45  1.04   0.05
## 12       4 -48.42 105.66  1.26   0.05
## 126      5 -47.40 106.04  1.64   0.04
## 2        3 -49.85 106.18  1.78   0.04
## 23       4 -48.70 106.22  1.81   0.04
## 26       4 -48.84 106.49  2.08   0.03
## 236      5 -47.63 106.52  2.11   0.03
## 245      5 -47.76 106.76  2.36   0.03
## 2456     6 -46.59 106.97  2.56   0.02
## 1235     6 -46.81 107.42  3.01   0.02
## 12356    7 -45.58 107.60  3.19   0.02
## 1245     6 -46.93 107.65  3.24   0.02
## 123      5 -48.25 107.75  3.35   0.02
## 12456    7 -45.66 107.76  3.36   0.02
## 2345     6 -47.01 107.80  3.40   0.02
## 124      5 -48.41 108.08  3.67   0.01
## 23456    7 -45.83 108.09  3.68   0.01
## 6        3 -50.83 108.14  3.73   0.01
## 1236     6 -47.21 108.21  3.81   0.01
## 24       4 -49.71 108.23  3.83   0.01
## 234      5 -48.60 108.45  4.04   0.01
## (Null)   2 -52.11 108.46  4.05   0.01
## 1246     6 -47.40 108.58  4.17   0.01
## 246      5 -48.73 108.71  4.31   0.01
## 2346     6 -47.57 108.92  4.52   0.01
## 36       4 -50.18 109.17  4.76   0.01
## 3        3 -51.41 109.30  4.90   0.01
## 56       4 -50.36 109.53  5.13   0.01
## 5        3 -51.64 109.77  5.36   0.01
## 12345    7 -46.81 110.06  5.65   0.01
## 1234     6 -48.23 110.24  5.84   0.00
## 123456   8 -45.58 110.36  5.95   0.00
## 16       4 -50.80 110.41  6.01   0.00
## 46       4 -50.82 110.45  6.05   0.00
```

```
## 1       3 -52.09 110.66  6.25   0.00
## 4       3 -52.11 110.70  6.30   0.00
## 12346   7 -47.21 110.85  6.44   0.00
## 356     5 -49.82 110.88  6.48   0.00
## 35      4 -51.04 110.89  6.48   0.00
## 136     5 -49.98 111.21  6.80   0.00
## 13      4 -51.20 111.22  6.81   0.00
## 346     5 -50.17 111.59  7.18   0.00
## 34      4 -51.41 111.64  7.23   0.00
## 456     5 -50.31 111.87  7.46   0.00
## 156     5 -50.36 111.97  7.56   0.00
## 45      4 -51.62 112.06  7.65   0.00
## 15      4 -51.64 112.10  7.70   0.00
## 135     5 -50.63 112.52  8.11   0.00
## 1356    6 -49.46 112.71  8.31   0.00
## 146     5 -50.78 112.81  8.40   0.00
## 14      4 -52.08 112.98  8.58   0.00
## 345     5 -51.02 113.29  8.88   0.00
## 3456    6 -49.77 113.33  8.93   0.00
## 134     5 -51.18 113.62  9.21   0.00
## 1346    6 -49.98 113.74  9.33   0.00
## 1456    6 -50.31 114.40 10.00   0.00
##
## Term codes:
##       lam(dcon)      lam(drios) lam(tree_1000m)         p(date)         p(dras)
##               1               2               3               4               5
##         p(sfrz)
##               6
##
## Model-averaged coefficients:
## (full average)
##                 Estimate Std. Error z value Pr(>|z|)
## lam(Int)         1.52015    1.27708   1.190 0.233916
## lam(drios)       0.88469    0.49283   1.795 0.072631 .
## p(Int)          -5.67069    1.63013   3.479 0.000504 ***
## p(dras)          0.46155    0.50749   0.909 0.363100
## p(sfrz)          0.81718    1.22249   0.668 0.503844
## lam(dcon)       -0.24026    0.49004   0.490 0.623938
## lam(tree_1000m)  0.11231    0.24279   0.463 0.643657
## p(date)         -0.01886    0.25354   0.074 0.940715
##
## (conditional average)
##                 Estimate Std. Error z value Pr(>|z|)
## lam(Int)         1.52015    1.27708   1.190 0.233916
## lam(drios)       0.98891    0.41040   2.410 0.015970 *
## p(Int)          -5.67069    1.63013   3.479 0.000504 ***
## p(dras)          0.76738    0.43991   1.744 0.081089 .
## p(sfrz)          1.70479    1.26672   1.346 0.178356
## lam(dcon)       -0.63409    0.61973   1.023 0.306221
## lam(tree_1000m)  0.32008    0.31858   1.005 0.315041
## p(date)         -0.08327    0.52773   0.158 0.874628
## ---
## Signif. codes:  0 '***' 0.001 '**' 0.01 '*' 0.05 '.' 0.1 ' ' 1
```

```r
ss <- match(rownames(get(sprintf("UMF.%s",spp))@y),cam.data$cdg)
prd <- predict(get(sprintf("mavg%s.%s",mod,spp)),type='state')
dtf <- data.frame(fit=prd$fit, se.fit=prd$se.fit, hunting=cam.data[ss,"hunting"], dcon=cam.data[ss,"dco
ggplot(dtf, aes(y=dcon, x=tree_1000m,size=fit,colour=se.fit)) +
    geom_point() + ylab("Distance to conucos  (m)") + xlab("Tree cover (% in 1km buffer)") +
    labs(title=spp,size='Predicted values',colour='Prediction S.E.')
```

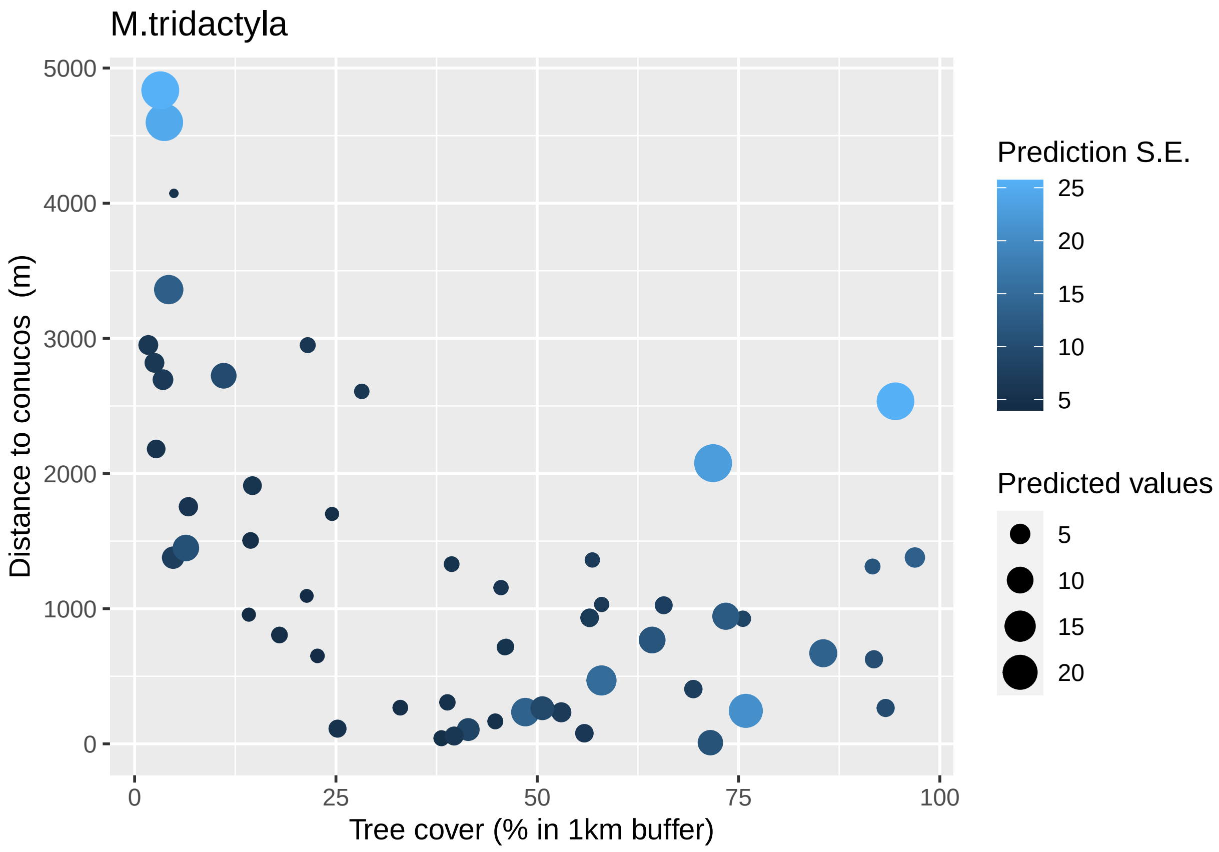

L.pardalis

No sign of lack of fit, c-hat > 1 overdispersion (used to adjust standard errors)

```r
spp <- "L.pardalis"
mod <- ifelse(spp %in% with.quad.term,"03","01")

 tbl1 %>% filter(species %in% spp) %>% select(1:5)
```

```
##      species n.detect chi.square p.value c.hat.est
## 1 L.pardalis       14   1427.131  0.1279  1.773193
```

Most support for variables:

```r
sw(get(sprintf("oms%s.%s",mod,spp)))
```

```
##                        lam(tree_1000m) lam(drios) lam(dcon) p(dras) p(sfrz)
## Sum of weights:        0.35            0.26       0.26      0.25    0.24
## N containing models:     32              32         32        32      32
##                        p(date)
## Sum of weights:        0.23
```

```
## N containing models:   32
```

Summary of model averaging estimates (use conditional average):

```r
summary(get(sprintf("mavg%s.%s",mod,spp)))
```

```
##
## Call:
## model.avg(object = get.models(object = oms01, subset = delta <
##     10))
##
## Component model call:
## occuRN(formula = ~<57 unique rhs>, data = UMF, K = 50)
##
## Component models:
##        df logLik QAICc delta weight
## (Null)  2 -52.56 65.76  0.00   0.14
## 3       3 -51.37 66.76  0.99   0.09
## 1       3 -52.01 67.48  1.71   0.06
## 2       3 -52.11 67.59  1.83   0.06
## 5       3 -52.31 67.81  2.05   0.05
## 6       3 -52.37 67.88  2.12   0.05
## 4       3 -52.56 68.10  2.34   0.04
## 23      4 -51.00 68.78  3.01   0.03
## 35      4 -51.17 68.96  3.20   0.03
## 36      4 -51.20 69.00  3.24   0.03
## 13      4 -51.28 69.08  3.32   0.03
## 34      4 -51.37 69.19  3.42   0.03
## 16      4 -51.82 69.70  3.94   0.02
## 15      4 -51.87 69.75  3.99   0.02
## 12      4 -51.87 69.76  3.99   0.02
## 26      4 -51.88 69.77  4.00   0.02
## 25      4 -51.96 69.85  4.09   0.02
## 14      4 -52.00 69.90  4.14   0.02
## 24      4 -52.10 70.01  4.25   0.02
## 56      4 -52.12 70.04  4.28   0.02
## 45      4 -52.30 70.24  4.47   0.02
## 46      4 -52.37 70.32  4.55   0.01
## 236     5 -50.81 71.10  5.33   0.01
## 235     5 -50.87 71.16  5.40   0.01
## 123     5 -51.00 71.31  5.55   0.01
## 234     5 -51.00 71.31  5.55   0.01
## 356     5 -51.01 71.32  5.55   0.01
## 136     5 -51.11 71.44  5.67   0.01
## 135     5 -51.11 71.44  5.67   0.01
## 345     5 -51.17 71.50  5.74   0.01
## 346     5 -51.20 71.54  5.77   0.01
## 134     5 -51.28 71.62  5.86   0.01
## 126     5 -51.67 72.06  6.30   0.01
## 156     5 -51.69 72.09  6.32   0.01
## 256     5 -51.74 72.15  6.39   0.01
## 125     5 -51.75 72.16  6.40   0.01
## 146     5 -51.80 72.21  6.45   0.01
## 145     5 -51.84 72.26  6.50   0.01
## 246     5 -51.86 72.28  6.52   0.01
```

```
## 124     5 -51.86 72.28  6.52   0.01
## 245     5 -51.93 72.36  6.60   0.01
## 456     5 -52.10 72.55  6.79   0.00
## 2356    6 -50.69 73.61  7.84   0.00
## 2346    6 -50.81 73.74  7.98   0.00
## 1236    6 -50.81 73.74  7.98   0.00
## 2345    6 -50.86 73.80  8.04   0.00
## 1235    6 -50.87 73.81  8.04   0.00
## 1356    6 -50.95 73.91  8.14   0.00
## 3456    6 -51.00 73.96  8.19   0.00
## 1234    6 -51.00 73.96  8.20   0.00
## 1345    6 -51.11 74.08  8.32   0.00
## 1346    6 -51.11 74.08  8.32   0.00
## 1256    6 -51.56 74.59  8.83   0.00
## 1456    6 -51.64 74.68  8.92   0.00
## 1246    6 -51.64 74.68  8.92   0.00
## 2456    6 -51.70 74.75  8.99   0.00
## 1245    6 -51.73 74.78  9.01   0.00
##
## Term codes:
##       lam(dcon)      lam(drios) lam(tree_1000m)         p(date)         p(dras)
##               1               2               3               4               5
##         p(sfrz)
##               6
##
## Model-averaged coefficients:
## (full average)
##                  Estimate Std. Error z value Pr(>|z|)
## lam(Int)        -0.861792   0.674129   1.278  0.20112
## p(Int)          -2.336286   0.836814   2.792  0.00524 **
## lam(tree_1000m)  0.171864   0.314199   0.547  0.58438
## lam(dcon)       -0.126765   0.431781   0.294  0.76907
## lam(drios)      -0.093753   0.284728   0.329  0.74195
## p(dras)          0.065857   0.242115   0.272  0.78562
## p(sfrz)          0.146831   0.563360   0.261  0.79437
## p(date)          0.008511   0.258035   0.033  0.97369
##
## (conditional average)
##                 Estimate Std. Error z value Pr(>|z|)
## lam(Int)        -0.86179    0.67413   1.278  0.20112
## p(Int)          -2.33629    0.83681   2.792  0.00524 **
## lam(tree_1000m)  0.49957    0.35106   1.423  0.15473
## lam(dcon)       -0.49407    0.73835   0.669  0.50340
## lam(drios)      -0.36103    0.46443   0.777  0.43694
## p(dras)          0.27137    0.43102   0.630  0.52895
## p(sfrz)          0.60641    1.01590   0.597  0.55056
## p(date)          0.03808    0.54477   0.070  0.94427
## ---
## Signif. codes:  0 '***' 0.001 '**' 0.01 '*' 0.05 '.' 0.1 ' ' 1
```

```r
ss <- match(rownames(get(sprintf("UMF.%s",spp))@y),cam.data$cdg)
prd <- predict(get(sprintf("mavg%s.%s",mod,spp)),type='state')
dtf <- data.frame(fit=prd$fit, se.fit=prd$se.fit, hunting=cam.data[ss,"hunting"], dcon=cam.data[ss,"dco
ggplot(dtf, aes(y=dcon, x=tree_1000m,size=fit,colour=se.fit)) +
```

```
    geom_point() + ylab("Distance to conucos  (m)") + xlab("Tree cover (% in 1km buffer)") +
    labs(title=spp,size='Predicted values',colour='Prediction S.E.')
```

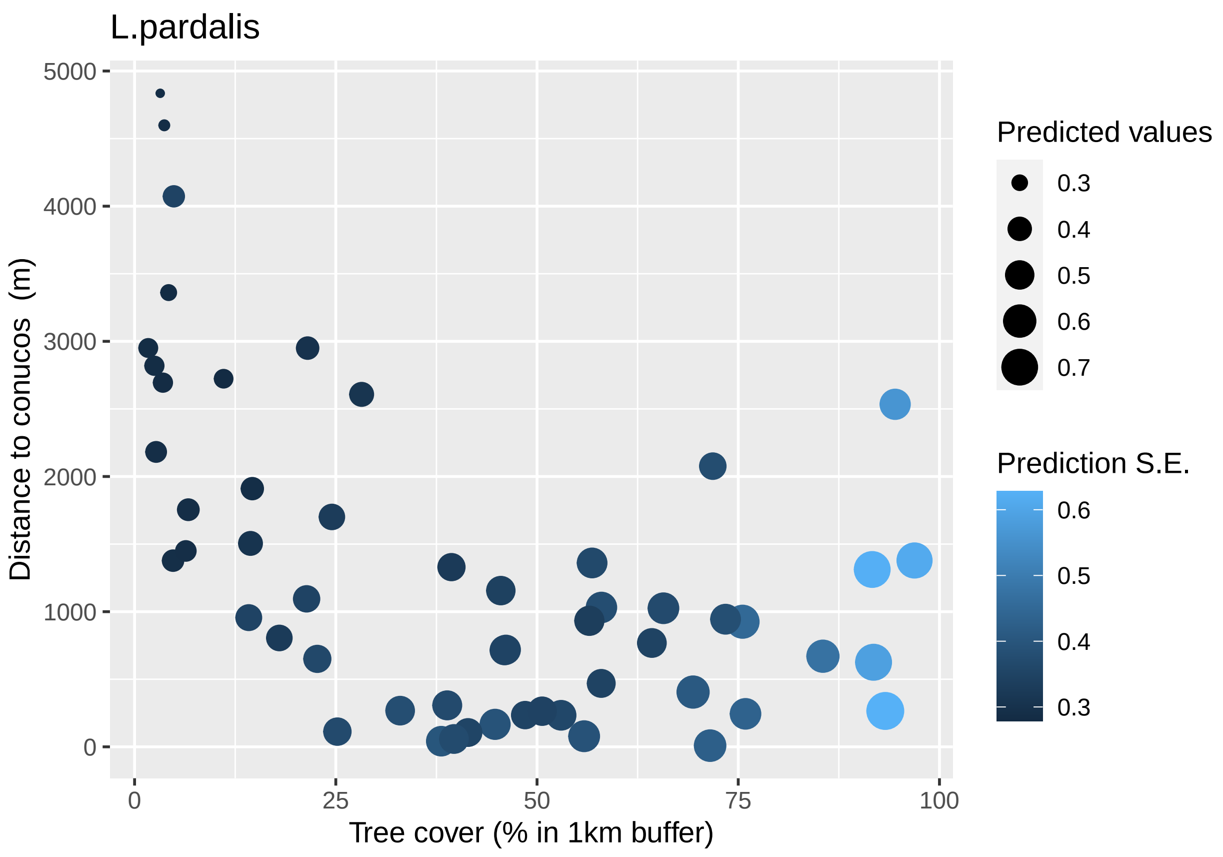

E.barbara

No sign of lack of fit, c-hat <1 : But prediction unrealistic (too high)

```
spp <- "E.barbara"
mod <- ifelse(spp %in% with.quad.term,"03","01")

 tbl1 %>% filter(species %in% spp) %>% select(1:5)
```

```
##     species n.detect chi.square p.value c.hat.est
## 1 E.barbara       16   282.1365  0.6953 0.4064038
```

Most support for variables:

```
sw(get(sprintf("oms%s.%s",mod,spp)))
```

```
##                      lam(tree_1000m) lam(dcon) p(sfrz) lam(drios)
## Sum of weights:      0.87            0.84      0.24    0.24
## N containing models:   64              48        48      48
##                      lam(I(tree_1000m^2)) p(date) p(dras)
## Sum of weights:      0.23                 0.23    0.22
## N containing models:   32                   48      48
```

Summary of model averaging estimates (use conditional average):

```r
summary(get(sprintf("mavg%s.%s",mod,spp)))
```

```
##
## Call:
## model.avg(object = get.models(object = oms03, subset = delta <
##     10))
##
## Component model call:
## occuRN(formula = ~<64 unique rhs>, data = UMF, K = 50)
##
## Component models:
##        df logLik   AICc delta weight
## 14      4 -52.76 114.34  0.00   0.19
## 147     5 -52.66 116.58  2.24   0.06
## 134     5 -52.67 116.59  2.25   0.06
## 145     5 -52.75 116.75  2.42   0.06
## 124     5 -52.75 116.75  2.42   0.06
## 146     5 -52.76 116.77  2.43   0.06
## 1       3 -55.46 117.40  3.06   0.04
## 4       3 -55.90 118.28  3.94   0.03
## 34      4 -55.05 118.92  4.58   0.02
## 1347    6 -52.58 118.94  4.61   0.02
## 1234    6 -52.62 119.02  4.69   0.02
## 1457    6 -52.65 119.08  4.75   0.02
## 1345    6 -52.65 119.09  4.75   0.02
## 1247    6 -52.65 119.10  4.76   0.02
## 1467    6 -52.66 119.12  4.78   0.02
## 1346    6 -52.67 119.12  4.79   0.02
## 1245    6 -52.74 119.28  4.94   0.02
## 1456    6 -52.75 119.29  4.95   0.02
## 1246    6 -52.75 119.29  4.95   0.02
## 17      4 -55.28 119.37  5.03   0.02
## 15      4 -55.35 119.52  5.19   0.01
## 12      4 -55.46 119.73  5.39   0.01
## 16      4 -55.46 119.73  5.40   0.01
## 24      4 -55.49 119.79  5.46   0.01
## 47      4 -55.75 120.31  5.97   0.01
## 234     5 -54.56 120.37  6.03   0.01
## 46      4 -55.83 120.48  6.15   0.01
## 45      4 -55.83 120.49  6.15   0.01
## 347     5 -54.93 121.12  6.78   0.01
## 346     5 -55.05 121.35  7.01   0.01
## 345     5 -55.05 121.35  7.02   0.01
## 12347   7 -52.53 121.49  7.15   0.01
## 157     5 -55.14 121.52  7.19   0.01
## 13457   7 -52.55 121.53  7.19   0.01
## 13467   7 -52.58 121.59  7.25   0.01
## 12345   7 -52.60 121.63  7.29   0.01
## 12346   7 -52.61 121.65  7.32   0.00
## 12457   7 -52.64 121.71  7.38   0.00
## 14567   7 -52.65 121.73  7.39   0.00
## 13456   7 -52.65 121.73  7.40   0.00
## 12467   7 -52.65 121.74  7.41   0.00
## 167     5 -55.28 121.80  7.47   0.00
```

```
## 127     5 -55.28 121.80  7.47   0.00
## 247     5 -55.32 121.88  7.55   0.00
## 12456   7 -52.74 121.92  7.58   0.00
## 125     5 -55.35 121.95  7.61   0.00
## 156     5 -55.35 121.96  7.62   0.00
## 245     5 -55.45 122.15  7.81   0.00
## 126     5 -55.46 122.16  7.83   0.00
## 246     5 -55.48 122.21  7.87   0.00
## 467     5 -55.69 122.63  8.29   0.00
## 2347    6 -54.43 122.64  8.31   0.00
## 457     5 -55.70 122.65  8.32   0.00
## 2346    6 -54.48 122.74  8.41   0.00
## 456     5 -55.79 122.84  8.50   0.00
## 2345    6 -54.56 122.90  8.57   0.00
## 3467    6 -54.93 123.64  9.31   0.00
## 3457    6 -54.93 123.65  9.32   0.00
## 3456    6 -55.05 123.88  9.55   0.00
## 1257    6 -55.13 124.05  9.72   0.00
## 1567    6 -55.14 124.06  9.72   0.00
## 123457  8 -52.49 124.18  9.85   0.00
## 123467  8 -52.52 124.23  9.90   0.00
## 134567  8 -52.55 124.29  9.96   0.00
##
## Term codes:
##           lam(dcon)           lam(drios) lam(I(tree_1000m^2))
##                   1                    2                    3
##      lam(tree_1000m)              p(date)              p(dras)
##                   4                    5                    6
##             p(sfrz)
##                   7
##
## Model-averaged coefficients:
## (full average)
##                       Estimate Std. Error z value Pr(>|z|)
## lam(Int)               0.837624   1.351571   0.620    0.535
## lam(dcon)             -1.784306   1.201836   1.485    0.138
## lam(tree_1000m)        0.806467   0.629903   1.280    0.200
## p(Int)                -5.113520   1.007256   5.077    4e-07 ***
## p(sfrz)                0.098980   0.471376   0.210    0.834
## lam(I(tree_1000m^2)) -0.067226   0.265115   0.254    0.800
## p(date)                0.015204   0.212007   0.072    0.943
## lam(drios)            -0.023838   0.199382   0.120    0.905
## p(dras)                0.001795   0.196068   0.009    0.993
##
## (conditional average)
##                       Estimate Std. Error z value Pr(>|z|)
## lam(Int)               0.837624   1.351571   0.620   0.5354
## lam(dcon)             -2.097631   1.020200   2.056   0.0398 *
## lam(tree_1000m)        0.924982   0.587760   1.574   0.1155
## p(Int)                -5.113520   1.007256   5.077    4e-07 ***
## p(sfrz)                0.417023   0.896397   0.465   0.6418
## lam(I(tree_1000m^2)) -0.289198   0.488024   0.593   0.5535
## p(date)                0.068966   0.447411   0.154   0.8775
## lam(drios)            -0.103903   0.406142   0.256   0.7981
```

```
## p(dras)              0.008349   0.422742   0.020   0.9842
## ---
## Signif. codes:  0 '***' 0.001 '**' 0.01 '*' 0.05 '.' 0.1 ' ' 1
```

```
ss <- match(rownames(get(sprintf("UMF.%s",spp))@y),cam.data$cdg)
prd <- predict(get(sprintf("mavg%s.%s",mod,spp)),type='state')
dtf <- data.frame(fit=prd$fit, se.fit=prd$se.fit, hunting=cam.data[ss,"hunting"], dcon=cam.data[ss,"dcon
ggplot(dtf, aes(y=dcon, x=tree_1000m,size=fit,colour=se.fit)) +
    geom_point() + ylab("Distance to conucos  (m)") + xlab("Tree cover (% in 1km buffer)") +
    labs(title=spp,size='Predicted values',colour='Prediction S.E.')
```

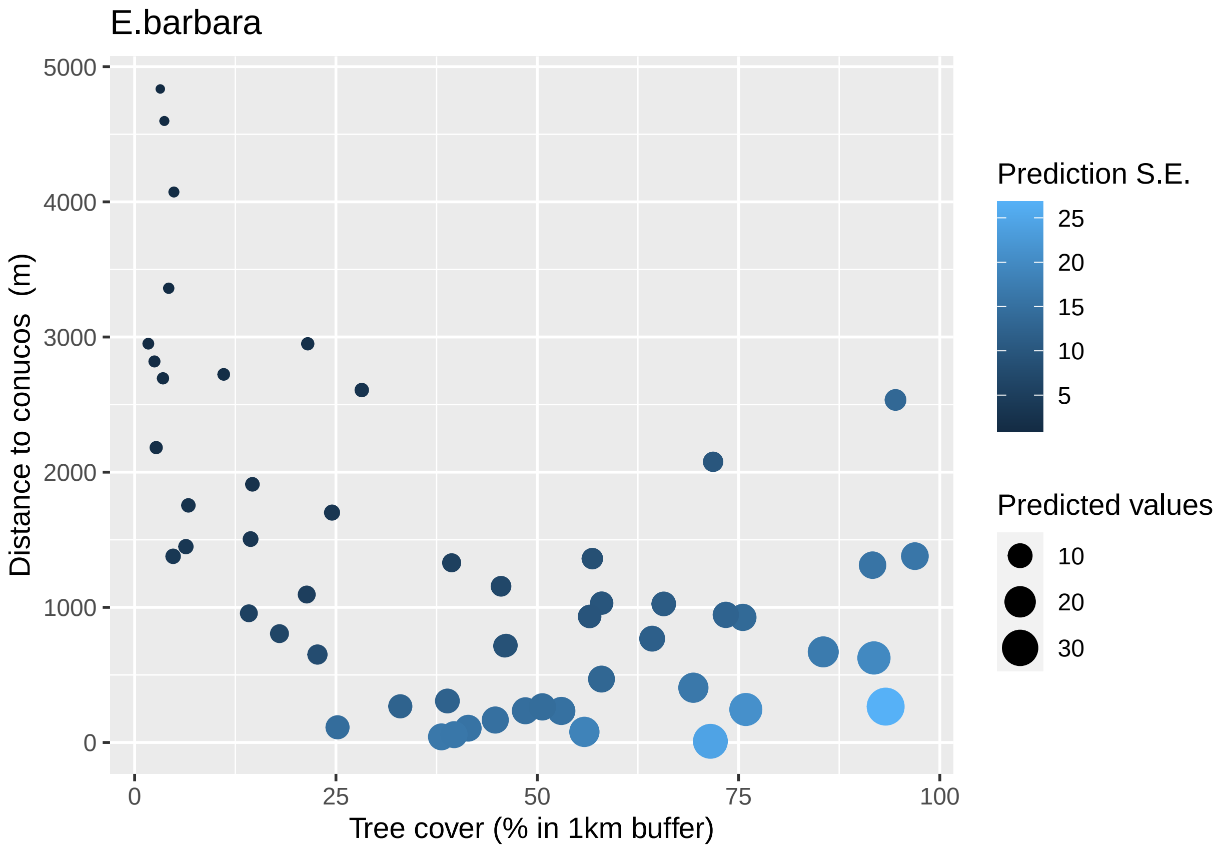

D.novemcinctus

No sign of lack of fit, c-hat > 1 overdispersion (used to adjust standard errors)

```
spp <- "D.novemcinctus"
mod <- ifelse(spp %in% with.quad.term,"03","01")

 tbl1 %>% filter(species %in% spp) %>% select(1:5)
```

```
##          species n.detect chi.square p.value c.hat.est
## 1 D.novemcinctus       17   956.2648  0.1418  1.285367
```

Most support for variables:

```
sw(get(sprintf("oms%s.%s",mod,spp)))
```

```
##                      p(date) lam(tree_1000m) lam(dcon) lam(drios) p(dras)
## Sum of weights:      0.85    0.41            0.32      0.23       0.23
```

```
## N containing models:   32      32               32        32          32
##                       p(sfrz)
## Sum of weights:       0.22
## N containing models:   32
```

Summary of model averaging estimates (use conditional average):

```r
summary(get(sprintf("mavg%s.%s",mod,spp)))
```

```
##
## Call:
## model.avg(object = get.models(object = oms01, subset = delta <
##     10))
##
## Component model call:
## occuRN(formula = ~<56 unique rhs>, data = UMF, K = 50)
##
## Component models:
##        df logLik  QAICc delta weight
## 4       3 -56.66  96.98  0.00   0.14
## 34      4 -55.25  97.22  0.25   0.13
## 14      4 -55.75  98.00  1.02   0.09
## 45      4 -56.56  99.25  2.27   0.05
## 24      4 -56.62  99.34  2.37   0.04
## 46      4 -56.64  99.38  2.40   0.04
## 134     5 -55.09  99.51  2.54   0.04
## 234     5 -55.12  99.55  2.57   0.04
## 346     5 -55.21  99.70  2.72   0.04
## 345     5 -55.24  99.75  2.77   0.04
## 146     5 -55.72 100.49  3.52   0.02
## 145     5 -55.74 100.52  3.55   0.02
## 124     5 -55.75 100.54  3.56   0.02
## (Null)  2 -60.55 100.70  3.72   0.02
## 1       3 -59.29 101.06  4.09   0.02
## 3       3 -59.48 101.37  4.39   0.02
## 456     5 -56.53 101.74  4.77   0.01
## 245     5 -56.54 101.76  4.78   0.01
## 246     5 -56.60 101.85  4.87   0.01
## 1234    6 -55.03 102.06  5.08   0.01
## 1346    6 -55.05 102.10  5.12   0.01
## 2346    6 -55.08 102.14  5.17   0.01
## 1345    6 -55.09 102.16  5.18   0.01
## 2345    6 -55.12 102.20  5.22   0.01
## 3456    6 -55.20 102.33  5.35   0.01
## 5       3 -60.20 102.49  5.52   0.01
## 2       3 -60.37 102.75  5.77   0.01
## 6       3 -60.46 102.89  5.91   0.01
## 13      4 -58.94 102.96  5.99   0.01
## 1456    6 -55.72 103.13  6.15   0.01
## 1246    6 -55.72 103.14  6.16   0.01
## 1245    6 -55.74 103.17  6.19   0.01
## 16      4 -59.19 103.35  6.38   0.01
## 15      4 -59.21 103.38  6.40   0.01
## 35      4 -59.26 103.46  6.48   0.01
## 12      4 -59.28 103.49  6.52   0.01
```

```
## 23      4 -59.30 103.51  6.54   0.01
## 36      4 -59.38 103.64  6.66   0.01
## 2456    6 -56.51 104.37  7.39   0.00
## 12346   7 -54.99 104.77  7.79   0.00
## 56      4 -60.10 104.77  7.79   0.00
## 25      4 -60.13 104.81  7.83   0.00
## 12345   7 -55.03 104.82  7.85   0.00
## 13456   7 -55.05 104.86  7.88   0.00
## 23456   7 -55.08 104.91  7.93   0.00
## 26      4 -60.29 105.06  8.09   0.00
## 136     5 -58.84 105.34  8.36   0.00
## 135     5 -58.86 105.37  8.40   0.00
## 123     5 -58.93 105.48  8.50   0.00
## 156     5 -59.11 105.77  8.79   0.00
## 356     5 -59.15 105.82  8.84   0.00
## 235     5 -59.15 105.82  8.84   0.00
## 126     5 -59.19 105.88  8.91   0.00
## 12456   7 -55.71 105.89  8.91   0.00
## 125     5 -59.20 105.91  8.93   0.00
## 236     5 -59.20 105.91  8.93   0.00
##
## Term codes:
##       lam(dcon)      lam(drios) lam(tree_1000m)         p(date)         p(dras)
##               1               2               3               4               5
##         p(sfrz)
##               6
##
## Model-averaged coefficients:
## (full average)
##                 Estimate Std. Error z value Pr(>|z|)
## lam(Int)        -0.44460    0.73102   0.608  0.54306
## p(Int)          -2.69540    0.92344   2.919  0.00351 **
## p(date)         -1.20607    0.70516   1.710  0.08720 .
## lam(tree_1000m)  0.20297    0.32733   0.620  0.53520
## lam(dcon)       -0.22384    0.52031   0.430  0.66705
## p(dras)          0.03577    0.26640   0.134  0.89318
## lam(drios)      -0.02778    0.20749   0.134  0.89350
## p(sfrz)         -0.05447    0.43379   0.126  0.90007
##
## (conditional average)
##                 Estimate Std. Error z value Pr(>|z|)
## lam(Int)         -0.4446     0.7310   0.608  0.54306
## p(Int)           -2.6954     0.9234   2.919  0.00351 **
## p(date)          -1.4134     0.5383   2.626  0.00864 **
## lam(tree_1000m)   0.5009     0.3394   1.476  0.13996
## lam(dcon)        -0.7070     0.7166   0.987  0.32382
## p(dras)           0.1587     0.5434   0.292  0.77027
## lam(drios)       -0.1232     0.4232   0.291  0.77105
## p(sfrz)          -0.2457     0.8954   0.274  0.78378
## ---
## Signif. codes:  0 '***' 0.001 '**' 0.01 '*' 0.05 '.' 0.1 ' ' 1
```

```
ss <- match(rownames(get(sprintf("UMF.%s",spp))@y),cam.data$cdg)
prd <- predict(get(sprintf("mavg%s.%s",mod,spp)),type='state')
```

```r
dtf <- data.frame(fit=prd$fit, se.fit=prd$se.fit, hunting=cam.data[ss,"hunting"], dcon=cam.data[ss,"dcon
ggplot(dtf, aes(y=dcon, x=tree_1000m,size=fit,colour=se.fit)) +
    geom_point() + ylab("Distance to conucos  (m)") + xlab("Tree cover (% in 1km buffer)") +
    labs(title=spp,size='Predicted values',colour='Prediction S.E.')
```

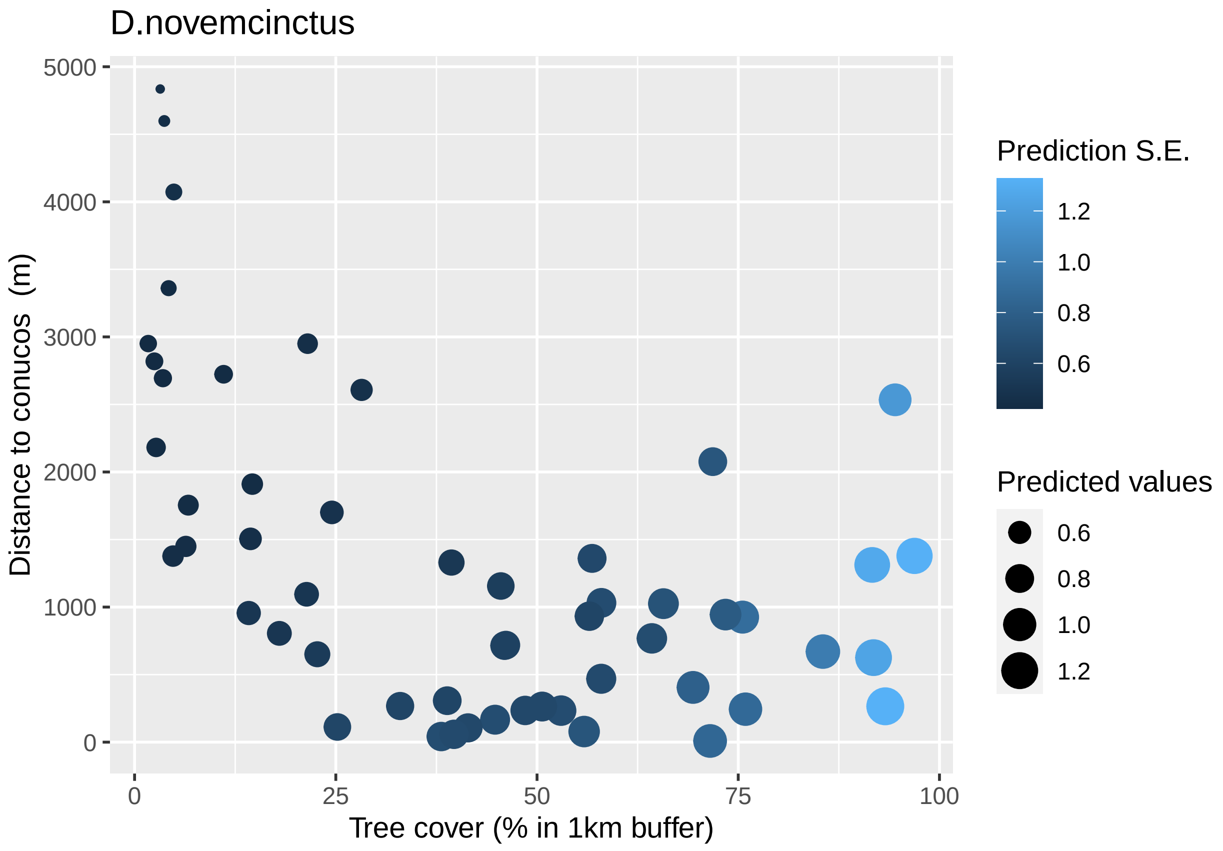

M.americana

No sign of lack of fit, c-hat values less than 1

```r
spp <- "M.americana"
mod <- ifelse(spp %in% with.quad.term,"03","01")

 tbl1 %>% filter(species %in% spp) %>% select(1:5)
```

```
##       species n.detect chi.square p.value c.hat.est
## 1 M.americana       17   242.7862  0.7573  0.317484
```

Most support for variables:

```r
sw(get(sprintf("oms%s.%s",mod,spp)))
```

```
##                        p(sfrz) lam(tree_1000m) p(date) lam(drios) lam(dcon)
## Sum of weights:        1.00    0.98            0.88    0.25       0.24
## N containing models:   32      32              32      32         32
##                        p(dras)
## Sum of weights:        0.23
## N containing models:   32
```

Summary of model averaging estimates (use conditional average): [Check coefficients (too high?)]

```r
summary(get(sprintf("mavg%s.%s",mod,spp)))
```

```
##
## Call:
## model.avg(object = get.models(object = oms01, subset = delta <
##     10))
##
## Component model call:
## occuRN(formula = ~<17 unique rhs>, data = UMF, K = 50)
##
## Component models:
##        df logLik   AICc delta weight
## 346     5 -40.07  91.39  0.00   0.39
## 2346    6 -39.90  93.59  2.20   0.13
## 3456    6 -40.02  93.84  2.44   0.12
## 1346    6 -40.03  93.84  2.45   0.12
## 36      4 -43.56  95.93  4.54   0.04
## 12346   7 -39.78  95.99  4.60   0.04
## 23456   7 -39.84  96.10  4.71   0.04
## 13456   7 -40.00  96.43  5.04   0.03
## 356     5 -43.00  97.26  5.86   0.02
## 136     5 -43.09  97.43  6.04   0.02
## 236     5 -43.47  98.18  6.79   0.01
## 123456  8 -39.75  98.69  7.30   0.01
## 1356    6 -42.77  99.34  7.94   0.01
## 1236    6 -42.81  99.41  8.02   0.01
## 2356    6 -42.85  99.48  8.09   0.01
## 146     5 -44.41 100.07  8.68   0.01
## 46      4 -46.27 101.36  9.97   0.00
##
## Term codes:
##       lam(dcon)      lam(drios) lam(tree_1000m)         p(date)         p(dras)
##               1               2               3               4               5
##         p(sfrz)
##               6
##
## Model-averaged coefficients:
## (full average)
##                 Estimate Std. Error z value Pr(>|z|)
## lam(Int)        -2.02466    0.68942   2.937  0.00332 **
## lam(tree_1000m)  1.42569    0.50834   2.805  0.00504 **
## p(Int)          -7.28819    2.43592   2.992  0.00277 **
## p(date)         -1.55978    0.88360   1.765  0.07752 .
## p(sfrz)          6.18262    2.47429   2.499  0.01246 *
## lam(drios)       0.05930    0.21944   0.270  0.78698
## p(dras)          0.06708    0.37205   0.180  0.85692
## lam(dcon)       -0.11300    0.60532   0.187  0.85192
##
## (conditional average)
##                 Estimate Std. Error z value Pr(>|z|)
## lam(Int)         -2.0247     0.6894   2.937  0.00332 **
## lam(tree_1000m)   1.4370     0.4942   2.907  0.00364 **
## p(Int)           -7.2882     2.4359   2.992  0.00277 **
```

```
## p(date)          -1.7639     0.7231   2.439  0.01471 *
## p(sfrz)           6.1826     2.4743   2.499  0.01246 *
## lam(drios)        0.2413     0.3899   0.619  0.53605
## p(dras)           0.2905     0.7312   0.397  0.69112
## lam(dcon)        -0.4781     1.1729   0.408  0.68355
## ---
## Signif. codes:  0 '***' 0.001 '**' 0.01 '*' 0.05 '.' 0.1 ' ' 1
```

```
ss <- match(rownames(get(sprintf("UMF.%s",spp))@y),cam.data$cdg)
prd <- predict(get(sprintf("mavg%s.%s",mod,spp)),type='state')
dtf <- data.frame(fit=prd$fit, se.fit=prd$se.fit, hunting=cam.data[ss,"hunting"], dcon=cam.data[ss,"dcon
ggplot(dtf, aes(y=dcon, x=tree_1000m,size=fit,colour=se.fit)) +
    geom_point() + ylab("Distance to conucos  (m)") + xlab("Tree cover (% in 1km buffer)") +
    labs(title=spp,size='Predicted values',colour='Prediction S.E.')
```

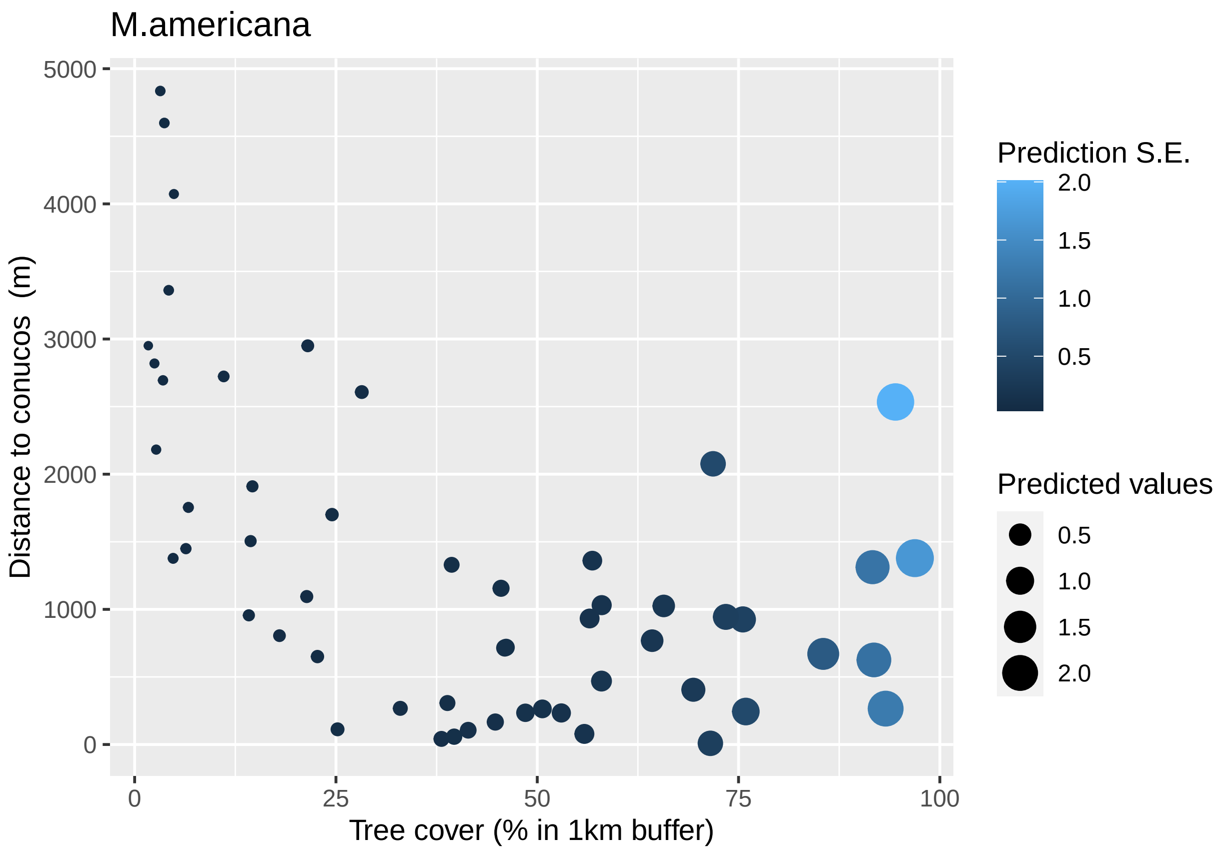

T.major

No sign of lack of fit, c-hat values less than 1 (maybe too low?)

```
spp <- "T.major"
mod <- ifelse(spp %in% with.quad.term,"03","01")

 tbl1 %>% filter(species %in% spp) %>% select(1:5)
```

```
##   species n.detect chi.square p.value c.hat.est
## 1 T.major       18   319.0557  0.9109 0.2109133
```

Most support for variables:

```r
sw(get(sprintf("oms%s.%s",mod,spp)))
```

```
##                        lam(tree_1000m) p(dras) lam(dcon) lam(drios) p(date)
## Sum of weights:        0.97            0.29    0.25      0.24       0.24
## N containing models:     32              32      32        32         32
##                        p(sfrz)
## Sum of weights:        0.23
## N containing models:     32
```

Summary of model averaging estimates (use conditional average):

```r
summary(get(sprintf("mavg%s.%s",mod,spp)))
```

```
##
## Call:
## model.avg(object = get.models(object = oms01, subset = delta <
##     10))
##
## Component model call:
## occuRN(formula = ~<36 unique rhs>, data = UMF, K = 50)
##
## Component models:
##         df logLik   AICc delta weight
## 3        3 -50.82 108.12  0.00   0.23
## 35       4 -50.57 109.96  1.83   0.09
## 23       4 -50.77 110.36  2.23   0.08
## 13       4 -50.79 110.39  2.26   0.08
## 36       4 -50.81 110.43  2.30   0.07
## 34       4 -50.81 110.44  2.32   0.07
## 135      5 -50.33 111.92  3.79   0.03
## 345      5 -50.44 112.12  4.00   0.03
## 235      5 -50.53 112.31  4.19   0.03
## 356      5 -50.56 112.37  4.24   0.03
## 123      5 -50.67 112.59  4.46   0.03
## 236      5 -50.75 112.75  4.63   0.02
## 234      5 -50.76 112.76  4.64   0.02
## 136      5 -50.77 112.78  4.66   0.02
## 134      5 -50.78 112.82  4.69   0.02
## 346      5 -50.79 112.84  4.71   0.02
## 1235     6 -50.12 114.03  5.91   0.01
## 1345     6 -50.23 114.26  6.13   0.01
## 1356     6 -50.32 114.42  6.30   0.01
## 2345     6 -50.39 114.56  6.44   0.01
## 3456     6 -50.42 114.62  6.50   0.01
## 2356     6 -50.52 114.82  6.70   0.01
## 1236     6 -50.64 115.07  6.94   0.01
## 1234     6 -50.67 115.12  7.00   0.01
## 2346     6 -50.74 115.26  7.13   0.01
## 1346     6 -50.76 115.31  7.19   0.01
## (Null)   2 -55.85 115.93  7.81   0.00
## 12345    7 -50.02 116.48  8.35   0.00
## 12356    7 -50.10 116.63  8.50   0.00
## 1        3 -55.13 116.74  8.62   0.00
## 13456    7 -50.21 116.86  8.73   0.00
## 23456    7 -50.37 117.17  9.04   0.00
```

```
## 12346   7 -50.64 117.71  9.59   0.00
## 4       3 -55.63 117.75  9.62   0.00
## 2       3 -55.75 117.98  9.85   0.00
## 5       3 -55.79 118.06  9.93   0.00
## 
## Term codes: 
##       lam(dcon)      lam(drios) lam(tree_1000m)         p(date)         p(dras) 
##               1               2               3               4               5 
##         p(sfrz) 
##               6 
## 
## Model-averaged coefficients:  
## (full average) 
##                 Estimate Std. Error z value Pr(>|z|)   
## lam(Int)        -1.89072    0.60283   3.136  0.00171 **
## lam(tree_1000m)  1.19739    0.44927   2.665  0.00769 **
## p(Int)          -1.35424    0.61794   2.192  0.02841 * 
## p(dras)          0.14388    0.39619   0.363  0.71649   
## lam(drios)      -0.03475    0.20283   0.171  0.86397   
## lam(dcon)        0.09300    0.45821   0.203  0.83916   
## p(sfrz)          0.04127    0.46150   0.089  0.92875   
## p(date)          0.03118    0.26810   0.116  0.90741   
##  
## (conditional average) 
##                 Estimate Std. Error z value Pr(>|z|)   
## lam(Int)         -1.8907     0.6028   3.136  0.00171 **
## lam(tree_1000m)   1.2132     0.4305   2.818  0.00483 **
## p(Int)           -1.3542     0.6179   2.192  0.02841 * 
## p(dras)           0.4962     0.6054   0.820  0.41244   
## lam(drios)       -0.1451     0.3946   0.368  0.71319   
## lam(dcon)         0.3739     0.8597   0.435  0.66363   
## p(sfrz)           0.1812     0.9539   0.190  0.84933   
## p(date)           0.1332     0.5416   0.246  0.80580   
## ---
## Signif. codes:  0 '***' 0.001 '**' 0.01 '*' 0.05 '.' 0.1 ' ' 1
```

```r
ss <- match(rownames(get(sprintf("UMF.%s",spp))@y),cam.data$cdg)
prd <- predict(get(sprintf("mavg%s.%s",mod,spp)),type='state')
dtf <- data.frame(fit=prd$fit, se.fit=prd$se.fit, hunting=cam.data[ss,"hunting"], dcon=cam.data[ss,"dcon
ggplot(dtf, aes(y=dcon, x=tree_1000m,size=fit,colour=se.fit)) +
    geom_point() + ylab("Distance to conucos  (m)") + xlab("Tree cover (% in 1km buffer)") +
    labs(title=spp,size='Predicted values',colour='Prediction S.E.')
```

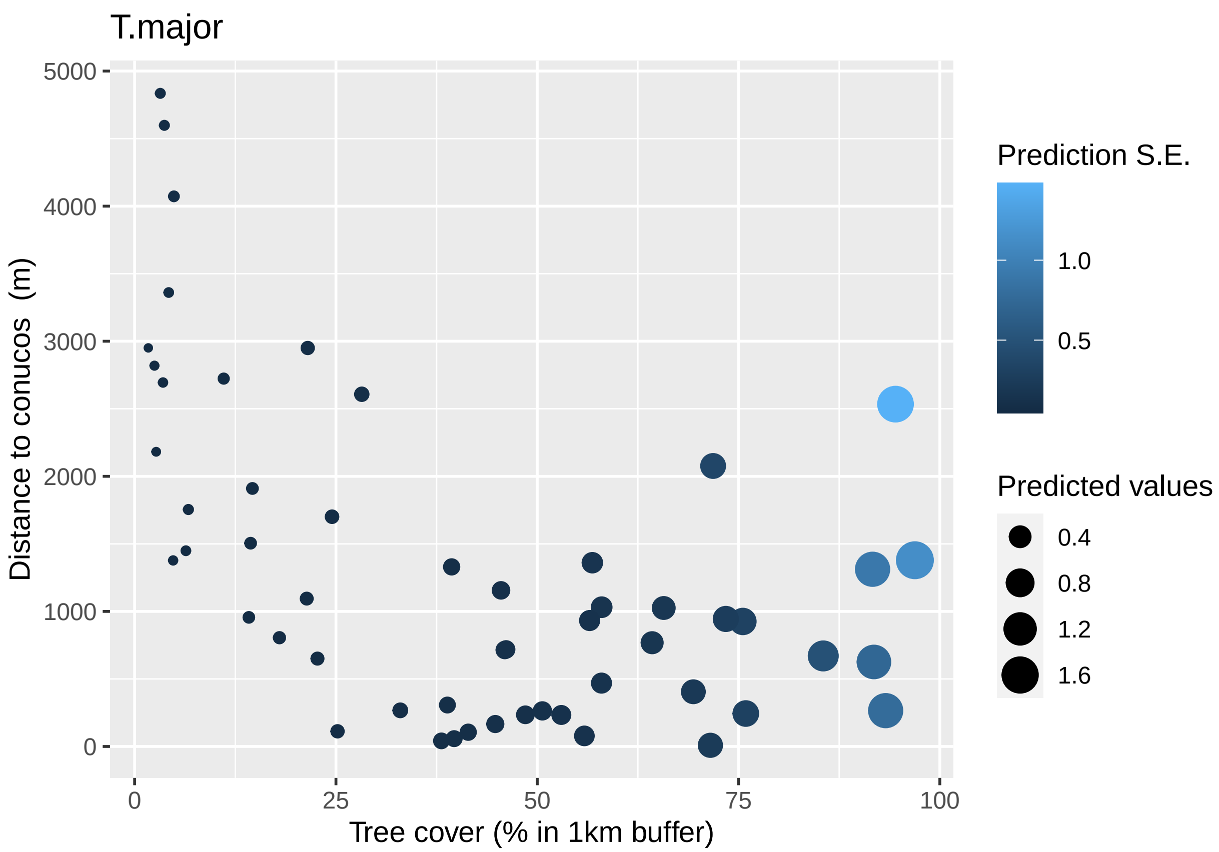

C.thous

No sign of lack of fit, c-hat values less than 1

```
spp <- "C.thous"
mod <- ifelse(spp %in% with.quad.term,"03","01")

 tbl1 %>% filter(species %in% spp) %>% select(1:5)
```

```
##   species n.detect chi.square p.value c.hat.est
## 1 C.thous       21   1217.307  0.4448 0.5860327
```

Most support for variables:

```
sw(get(sprintf("oms%s.%s",mod,spp)))
```

```
##                        lam(tree_1000m) p(sfrz) lam(dcon) lam(drios) p(dras)
## Sum of weights:        0.54            0.42    0.31      0.31       0.26
## N containing models:     32              32      32        32         32
##                        p(date)
## Sum of weights:        0.23
## N containing models:     32
```

Summary of model averaging estimates (use conditional average):

```
summary(get(sprintf("mavg%s.%s",mod,spp)))
```

```
##
## Call:
```

```
## model.avg(object = get.models(object = oms01, subset = delta <
##     10))
##
## Component model call:
## occuRN(formula = ~<64 unique rhs>, data = UMF, K = 50)
##
## Component models:
##        df logLik   AICc delta weight
## 3       3 -50.33 107.14  0.00   0.09
## 36      4 -49.44 107.70  0.56   0.07
## (Null)  2 -51.86 107.96  0.82   0.06
## 6       3 -51.02 108.52  1.38   0.05
## 23      4 -50.00 108.82  1.68   0.04
## 1       3 -51.26 109.01  1.87   0.04
## 35      4 -50.18 109.19  2.05   0.03
## 236     5 -49.06 109.37  2.24   0.03
## 13      4 -50.33 109.47  2.34   0.03
## 34      4 -50.33 109.47  2.34   0.03
## 16      4 -50.42 109.66  2.52   0.03
## 5       3 -51.64 109.76  2.62   0.03
## 356     5 -49.31 109.86  2.72   0.02
## 12      4 -50.53 109.87  2.73   0.02
## 2       3 -51.74 109.97  2.83   0.02
## 346     5 -49.44 110.12  2.98   0.02
## 136     5 -49.44 110.13  2.99   0.02
## 4       3 -51.86 110.20  3.06   0.02
## 126     5 -49.58 110.40  3.27   0.02
## 56      4 -50.82 110.45  3.31   0.02
## 26      4 -50.88 110.58  3.44   0.02
## 46      4 -51.01 110.83  3.69   0.01
## 235     5 -49.84 110.93  3.79   0.01
## 123     5 -49.84 110.93  3.79   0.01
## 15      4 -51.12 111.06  3.92   0.01
## 234     5 -49.99 111.24  4.10   0.01
## 14      4 -51.26 111.35  4.21   0.01
## 1236    6 -48.88 111.55  4.42   0.01
## 345     5 -50.17 111.59  4.45   0.01
## 135     5 -50.18 111.62  4.48   0.01
## 2356    6 -48.92 111.63  4.49   0.01
## 156     5 -50.29 111.83  4.69   0.01
## 25      4 -51.51 111.84  4.70   0.01
## 2346    6 -49.04 111.86  4.72   0.01
## 134     5 -50.33 111.91  4.77   0.01
## 45      4 -51.63 112.07  4.94   0.01
## 146     5 -50.42 112.08  4.94   0.01
## 125     5 -50.42 112.09  4.95   0.01
## 124     5 -50.50 112.26  5.12   0.01
## 24      4 -51.74 112.29  5.15   0.01
## 3456    6 -49.30 112.39  5.25   0.01
## 1356    6 -49.30 112.39  5.25   0.01
## 256     5 -50.67 112.60  5.46   0.01
## 1346    6 -49.44 112.66  5.52   0.01
## 1256    6 -49.50 112.79  5.65   0.01
## 1246    6 -49.52 112.83  5.69   0.01
```

```
## 456      5 -50.81 112.87  5.74   0.01
## 246      5 -50.87 112.98  5.84   0.01
## 1235     6 -49.72 113.23  6.09   0.00
## 1234     6 -49.82 113.44  6.30   0.00
## 2345     6 -49.83 113.45  6.32   0.00
## 145      5 -51.11 113.47  6.33   0.00
## 12356    7 -48.79 114.02  6.88   0.00
## 12346    7 -48.84 114.11  6.97   0.00
## 1345     6 -50.17 114.12  6.98   0.00
## 245      5 -51.50 114.26  7.12   0.00
## 23456    7 -48.92 114.28  7.14   0.00
## 1456     6 -50.29 114.37  7.23   0.00
## 1245     6 -50.42 114.62  7.48   0.00
## 13456    7 -49.30 115.03  7.90   0.00
## 2456     6 -50.67 115.13  7.99   0.00
## 12456    7 -49.49 115.41  8.27   0.00
## 12345    7 -49.72 115.88  8.74   0.00
## 123456   8 -48.78 116.77  9.63   0.00
##
## Term codes:
##       lam(dcon)      lam(drios) lam(tree_1000m)         p(date)         p(dras)
##               1               2               3               4               5
##         p(sfrz)
##               6
##
## Model-averaged coefficients:
## (full average)
##                  Estimate Std. Error z value Pr(>|z|)
## lam(Int)        -1.625398   0.375109   4.333 1.47e-05 ***
## lam(tree_1000m) -0.358238   0.461803   0.776    0.438
## p(Int)          -0.641664   0.871699   0.736    0.462
## p(sfrz)          0.606779   1.010982   0.600    0.548
## lam(drios)      -0.114682   0.303275   0.378    0.705
## lam(dcon)        0.136280   0.406504   0.335    0.737
## p(dras)         -0.052414   0.204854   0.256    0.798
## p(date)          0.007965   0.314492   0.025    0.980
##
## (conditional average)
##                 Estimate Std. Error z value Pr(>|z|)
## lam(Int)        -1.62540    0.37511   4.333 1.47e-05 ***
## lam(tree_1000m) -0.66083    0.43981   1.503    0.133
## p(Int)          -0.64166    0.87170   0.736    0.462
## p(sfrz)          1.44066    1.10696   1.301    0.193
## lam(drios)      -0.37186    0.45011   0.826    0.409
## lam(dcon)        0.43677    0.63116   0.692    0.489
## p(dras)         -0.20219    0.36277   0.557    0.577
## p(date)          0.03444    0.65327   0.053    0.958
## ---
## Signif. codes:  0 '***' 0.001 '**' 0.01 '*' 0.05 '.' 0.1 ' ' 1
```

```
ss <- match(rownames(get(sprintf("UMF.%s",spp))@y),cam.data$cdg)
prd <- predict(get(sprintf("mavg%s.%s",mod,spp)),type='state')
dtf <- data.frame(fit=prd$fit, se.fit=prd$se.fit, hunting=cam.data[ss,"hunting"], dcon=cam.data[ss,"dcon
ggplot(dtf, aes(y=dcon, x=tree_1000m,size=fit,colour=se.fit)) +
```

```
    geom_point() + ylab("Distance to conucos  (m)") + xlab("Tree cover (% in 1km buffer)") +
    labs(title=spp,size='Predicted values',colour='Prediction S.E.')
```

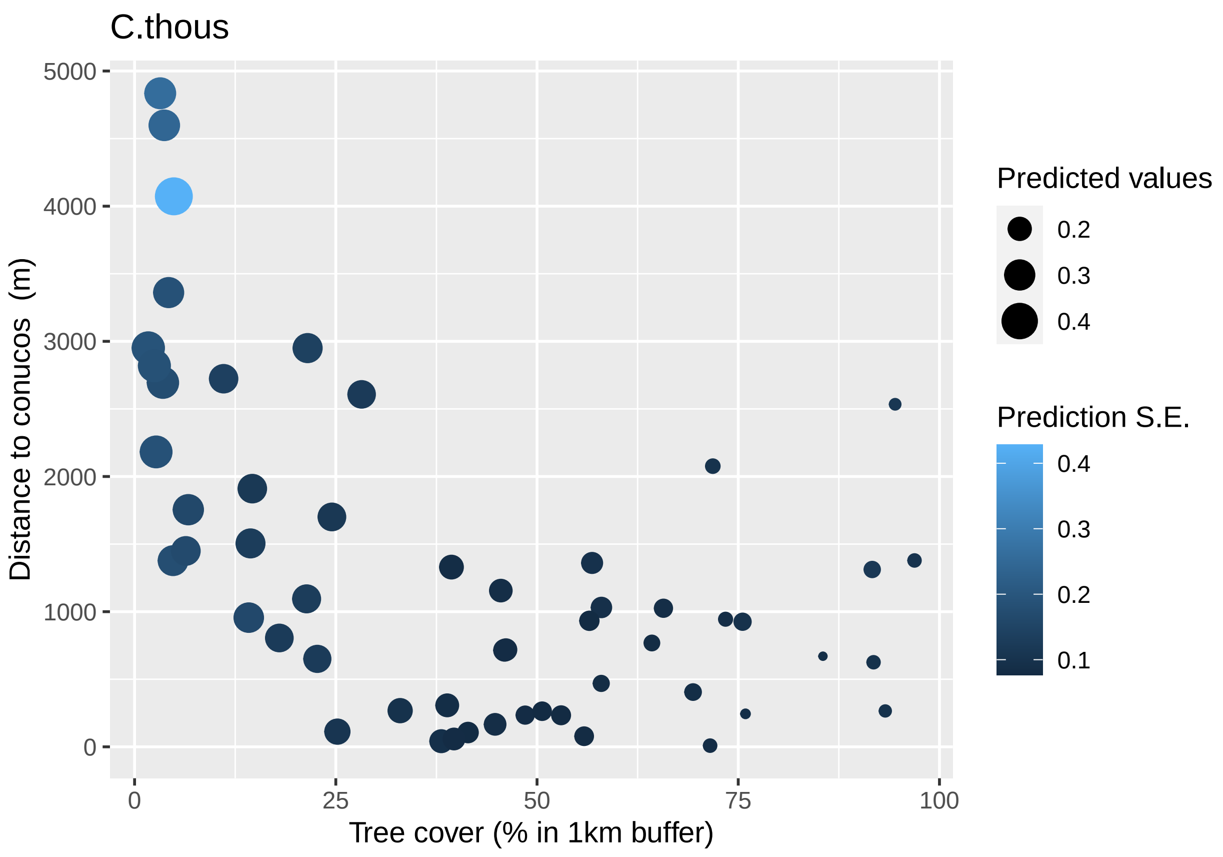

D.kappleri

No sign of lack of fit, c-hat values less than 1

```
spp <- "D.kappleri"
mod <- ifelse(spp %in% with.quad.term,"03","01")

 tbl1 %>% filter(species %in% spp) %>% select(1:5)
```

```
##      species n.detect chi.square p.value c.hat.est
## 1 D.kappleri       25   922.3673  0.4659 0.7568927
```

Most support for variables:

```
sw(get(sprintf("oms%s.%s",mod,spp)))
```

```
##                      lam(tree_1000m) p(sfrz) p(dras) lam(dcon) p(date)
## Sum of weights:      1.00            0.65    0.49    0.47      0.46
## N containing models:   32              32      32      32        32
##                      lam(drios)
## Sum of weights:      0.45
## N containing models:   32
```

Summary of model averaging estimates (use conditional average):

```r
summary(get(sprintf("mavg%s.%s",mod,spp)))
```

```
##
## Call:
## model.avg(object = get.models(object = oms01, subset = delta <
##     10))
##
## Component model call:
## occuRN(formula = ~<32 unique rhs>, data = UMF, K = 50)
##
## Component models:
##        df logLik   AICc delta weight
## 2346    6 -61.22 136.24  0.00   0.06
## 1356    6 -61.32 136.44  0.20   0.05
## 136     5 -62.60 136.46  0.22   0.05
## 356     5 -62.69 136.62  0.39   0.05
## 2356    6 -61.44 136.67  0.43   0.05
## 346     5 -62.74 136.73  0.50   0.05
## 1346    6 -61.54 136.86  0.62   0.04
## 236     5 -62.83 136.91  0.67   0.04
## 3456    6 -61.61 137.01  0.78   0.04
## 135     5 -62.96 137.18  0.94   0.04
## 23456   7 -60.37 137.18  0.94   0.04
## 36      4 -64.22 137.25  1.01   0.04
## 1236    6 -61.79 137.37  1.14   0.03
## 12356   7 -60.53 137.49  1.25   0.03
## 13      4 -64.35 137.51  1.27   0.03
## 12346   7 -60.56 137.55  1.31   0.03
## 13456   7 -60.57 137.58  1.35   0.03
## 35      4 -64.42 137.66  1.43   0.03
## 235     5 -63.31 137.88  1.64   0.03
## 134     5 -63.35 137.96  1.72   0.02
## 345     5 -63.42 138.09  1.86   0.02
## 234     5 -63.45 138.15  1.91   0.02
## 34      4 -64.73 138.27  2.03   0.02
## 1235    6 -62.26 138.30  2.06   0.02
## 1345    6 -62.28 138.35  2.12   0.02
## 2345    6 -62.34 138.47  2.23   0.02
## 123     5 -63.69 138.62  2.38   0.02
## 123456  8 -59.73 138.67  2.43   0.02
## 3       3 -66.14 138.76  2.52   0.02
## 23      4 -64.98 138.77  2.54   0.02
## 1234    6 -62.56 138.91  2.67   0.02
## 12345   7 -61.56 139.56  3.32   0.01
##
## Term codes:
##       lam(dcon)      lam(drios) lam(tree_1000m)         p(date)         p(dras)
##               1               2               3               4               5
##         p(sfrz)
##               6
##
## Model-averaged coefficients:
## (full average)
##                  Estimate Std. Error z value Pr(>|z|)
```

```
## lam(Int)          -0.9813     1.3775   0.712   0.4763
## lam(drios)        -0.2241     0.3433   0.653   0.5139
## lam(tree_1000m)    1.5173     0.3864   3.927 8.62e-05 ***
## p(Int)            -3.8593     1.6843   2.291   0.0219 *
## p(date)           -0.2657     0.3922   0.677   0.4982
## p(sfrz)            1.0020     1.0263   0.976   0.3289
## lam(dcon)         -0.6004     0.8795   0.683   0.4948
## p(dras)            0.3198     0.4378   0.730   0.4651
##
## (conditional average)
##                  Estimate Std. Error z value Pr(>|z|)
## lam(Int)          -0.9813     1.3775   0.712   0.4763
## lam(drios)        -0.5006     0.3533   1.417   0.1566
## lam(tree_1000m)    1.5173     0.3864   3.927 8.62e-05 ***
## p(Int)            -3.8593     1.6843   2.291   0.0219 *
## p(date)           -0.5763     0.3934   1.465   0.1429
## p(sfrz)            1.5491     0.8837   1.753   0.0796 .
## lam(dcon)         -1.2759     0.8842   1.443   0.1490
## p(dras)            0.6506     0.4179   1.557   0.1195
## ---
## Signif. codes:  0 '***' 0.001 '**' 0.01 '*' 0.05 '.' 0.1 ' ' 1
```

```r
ss <- match(rownames(get(sprintf("UMF.%s",spp))@y),cam.data$cdg)
prd <- predict(get(sprintf("mavg%s.%s",mod,spp)),type='state')
dtf <- data.frame(fit=prd$fit, se.fit=prd$se.fit, hunting=cam.data[ss,"hunting"], dcon=cam.data[ss,"dcon
ggplot(dtf, aes(y=dcon, x=tree_1000m,size=fit,colour=se.fit)) +
    geom_point() + ylab("Distance to conucos  (m)") + xlab("Tree cover (% in 1km buffer)") +
    labs(title=spp,size='Predicted values',colour='Prediction S.E.')
```

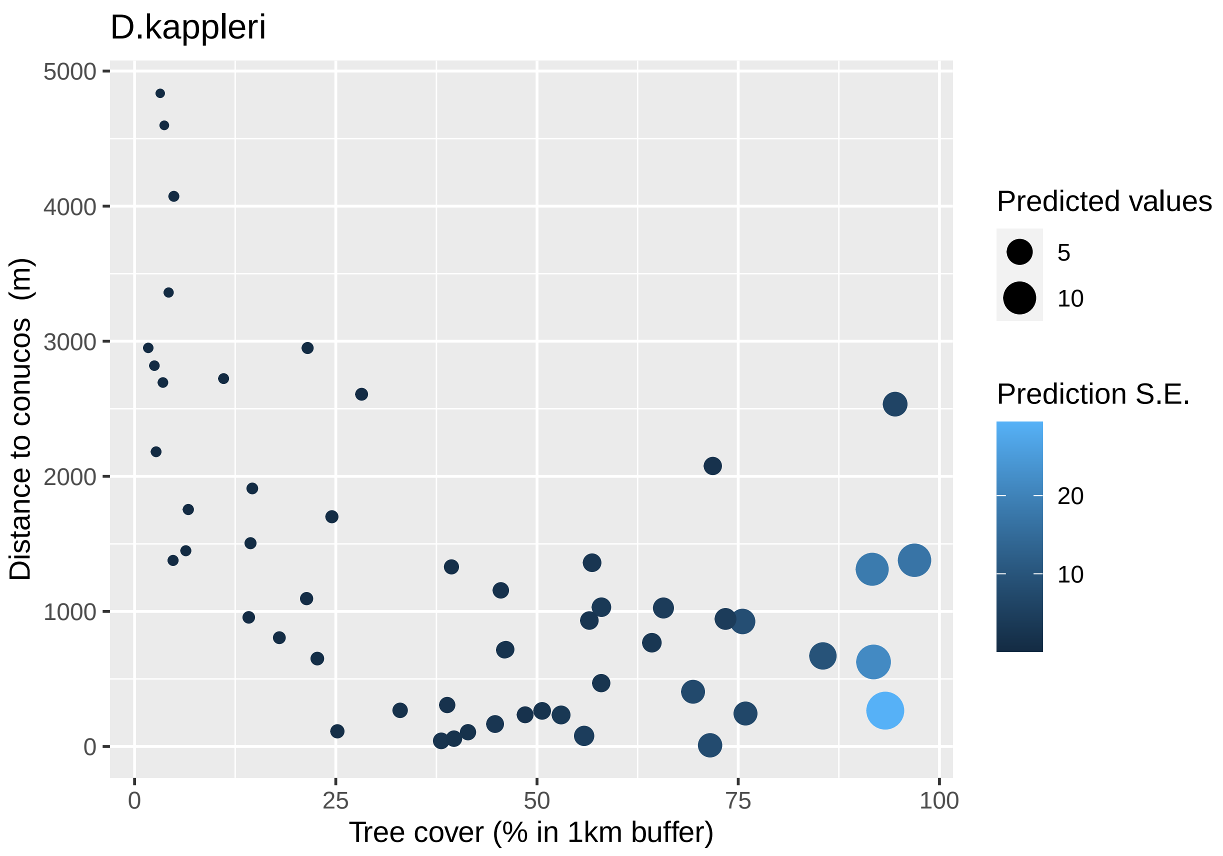

C.alector

No sign of lack of fit, c-hat values less than 1

```
spp <- "C.alector"
mod <- ifelse(spp %in% with.quad.term,"03","01")

 tbl1 %>% filter(species %in% spp) %>% select(1:5)
```

```
##     species n.detect chi.square p.value c.hat.est
## 1 C.alector       31    1098.64  0.5244 0.6225908
```

Most support for variables:

```
sw(get(sprintf("oms%s.%s",mod,spp)))
```

```
##                      lam(tree_1000m) p(sfrz) p(dras) lam(dcon) p(date)
## Sum of weights:      0.98            0.73    0.71    0.64      0.24
## N containing models:   32              32      32      32        32
##                      lam(drios)
## Sum of weights:      0.23
## N containing models:   32
```

Summary of model averaging estimates (use conditional average):

```
summary(get(sprintf("mavg%s.%s",mod,spp)))
```

```
##
## Call:
```

```
## model.avg(object = get.models(object = oms01, subset = delta <
##     10))
##
## Component model call:
## occuRN(formula = ~<33 unique rhs>, data = UMF, K = 50)
##
## Component models:
##        df logLik   AICc delta weight
## 1356    6 -67.24 148.26  0.00   0.24
## 135     5 -69.41 150.08  1.82   0.10
## 36      4 -70.79 150.40  2.14   0.08
## 12356   7 -67.19 150.81  2.55   0.07
## 356     5 -69.80 150.84  2.59   0.07
## 13456   7 -67.22 150.87  2.61   0.07
## 136     5 -70.30 151.85  3.59   0.04
## 346     5 -70.53 152.30  4.04   0.03
## 1235    6 -69.33 152.44  4.18   0.03
## 1345    6 -69.39 152.57  4.32   0.03
## 35      4 -71.94 152.69  4.43   0.03
## 236     5 -70.78 152.81  4.55   0.02
## 2356    6 -69.57 152.92  4.66   0.02
## 3       3 -73.26 153.00  4.74   0.02
## 3456    6 -69.79 153.37  5.12   0.02
## 123456  8 -67.17 153.53  5.27   0.02
## 1346    6 -69.90 153.60  5.34   0.02
## 1236    6 -70.26 154.30  6.04   0.01
## 235     5 -71.60 154.46  6.20   0.01
## 13      4 -72.87 154.55  6.29   0.01
## 34      4 -72.98 154.77  6.52   0.01
## 2346    6 -70.51 154.80  6.54   0.01
## 12345   7 -69.31 155.05  6.79   0.01
## 345     5 -71.94 155.12  6.86   0.01
## 23      4 -73.25 155.31  7.05   0.01
## 23456   7 -69.57 155.57  7.31   0.01
## 12346   7 -69.87 156.18  7.92   0.00
## 134     5 -72.48 156.21  7.96   0.00
## 6       3 -74.96 156.40  8.14   0.00
## 123     5 -72.85 156.94  8.68   0.00
## 2345    6 -71.60 157.00  8.74   0.00
## 234     5 -72.96 157.16  8.90   0.00
## 56      4 -74.41 157.65  9.39   0.00
##
## Term codes:
##      lam(dcon)      lam(drios) lam(tree_1000m)         p(date)         p(dras)
##              1               2               3               4               5
##        p(sfrz)
##              6
##
## Model-averaged coefficients:
## (full average)
##                 Estimate Std. Error z value Pr(>|z|)
## lam(Int)        -0.79852    0.61944   1.289   0.1974
## lam(dcon)        0.92375    0.90787   1.017   0.3089
## lam(tree_1000m)  1.10629    0.39767   2.782   0.0054 **
```

```
## p(Int)          -2.83407    1.16655   2.429   0.0151 *
## p(dras)          0.83791    0.68300   1.227   0.2199
## p(sfrz)          1.23388    1.04486   1.181   0.2376
## lam(drios)       0.02569    0.17240   0.149   0.8815
## p(date)         -0.03600    0.21755   0.165   0.8686
##
## (conditional average)
##                  Estimate Std. Error z value Pr(>|z|)
## lam(Int)          -0.7985     0.6194   1.289  0.19736
## lam(dcon)          1.4378     0.7374   1.950  0.05120 .
## lam(tree_1000m)    1.1133     0.3890   2.862  0.00421 **
## p(Int)            -2.8341     1.1666   2.429  0.01512 *
## p(dras)            1.1706     0.5121   2.286  0.02227 *
## p(sfrz)            1.6889     0.8519   1.982  0.04743 *
## lam(drios)         0.1124     0.3468   0.324  0.74587
## p(date)           -0.1549     0.4304   0.360  0.71892
## ---
## Signif. codes:  0 '***' 0.001 '**' 0.01 '*' 0.05 '.' 0.1 ' ' 1
```

```
ss <- match(rownames(get(sprintf("UMF.%s",spp))@y),cam.data$cdg)
prd <- predict(get(sprintf("mavg%s.%s",mod,spp)),type='state')
dtf <- data.frame(fit=prd$fit, se.fit=prd$se.fit, hunting=cam.data[ss,"hunting"], dcon=cam.data[ss,"dcon
ggplot(dtf, aes(y=dcon, x=tree_1000m,size=fit,colour=se.fit)) +
    geom_point() + ylab("Distance to conucos  (m)") + xlab("Tree cover (% in 1km buffer)") +
    labs(title=spp,size='Predicted values',colour='Prediction S.E.')
```

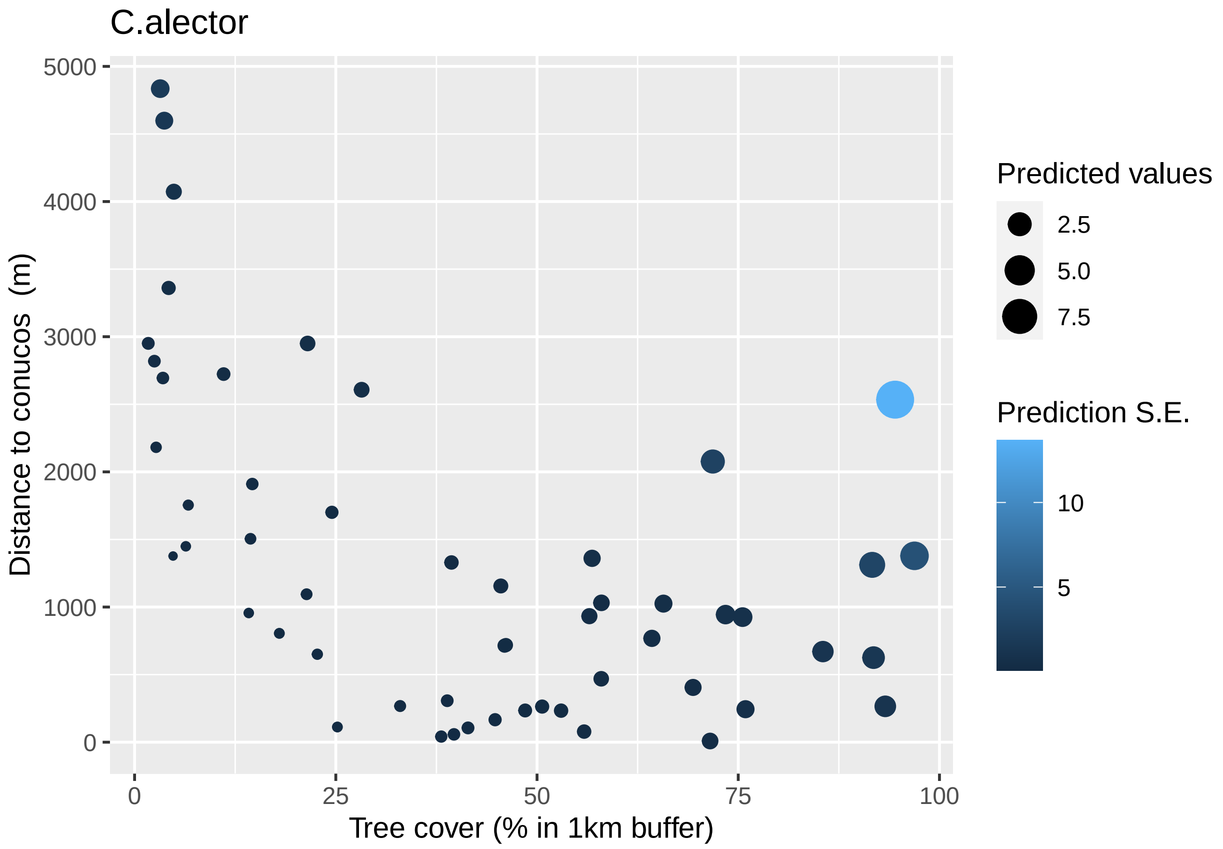

L.rufaxilla

No sign of lack of fit, c-hat values less than 1

```
spp <- "L.rufaxilla"
mod <- ifelse(spp %in% with.quad.term,"03","01")

 tbl1 %>% filter(species %in% spp) %>% select(1:5)
```

```
##       species n.detect chi.square p.value c.hat.est
## 1 L.rufaxilla       33   650.0822  0.6297 0.3598612
```

Most support for variables:

```
sw(get(sprintf("oms%s.%s",mod,spp)))
```

```
##                      p(sfrz) lam(tree_1000m) lam(I(tree_1000m^2)) p(date)
## Sum of weights:      1.00    0.85            0.79                 0.37
## N containing models:   48      64              32                   48
##                      lam(dcon) lam(drios) p(dras)
## Sum of weights:      0.31      0.23       0.22
## N containing models:   48        48         48
```

Summary of model averaging estimates (use conditional average):

```
summary(get(sprintf("mavg%s.%s",mod,spp)))
```

```
##
## Call:
## model.avg(object = get.models(object = oms03, subset = delta <
##     10))
##
## Component model call:
## occuRN(formula = ~<42 unique rhs>, data = UMF, K = 50)
##
## Component models:
##         df logLik   AICc delta weight
## 347      5 -69.58 150.40  0.00   0.22
## 3457     6 -68.76 151.31  0.91   0.14
## 1347     6 -69.46 152.70  2.30   0.07
## 2347     6 -69.51 152.81  2.41   0.07
## 3467     6 -69.52 152.83  2.43   0.07
## 13457    7 -68.61 153.66  3.26   0.04
## 23457    7 -68.66 153.76  3.36   0.04
## 34567    7 -68.76 153.96  3.56   0.04
## 17       4 -72.58 153.97  3.57   0.04
## 13467    7 -69.36 155.15  4.75   0.02
## 7        3 -74.38 155.23  4.83   0.02
## 12347    7 -69.42 155.28  4.88   0.02
## 23467    7 -69.46 155.35  4.95   0.02
## 157      5 -72.09 155.44  5.04   0.02
## 47       4 -73.50 155.83  5.43   0.01
## 147      5 -72.44 156.14  5.74   0.01
## 123457   8 -68.56 156.32  5.92   0.01
## 127      5 -72.56 156.37  5.97   0.01
## 134567   8 -68.60 156.40  6.00   0.01
## 167      5 -72.58 156.40  6.00   0.01
## 234567   8 -68.66 156.52  6.12   0.01
```

```
## 57       4 -73.94 156.69  6.29   0.01
## 27       4 -74.06 156.94  6.54   0.01
## 457      5 -73.16 157.56  7.16   0.01
## 67       4 -74.38 157.57  7.17   0.01
## 247      5 -73.23 157.70  7.30   0.01
## 1457     6 -72.00 157.78  7.38   0.01
## 123467   8 -69.33 157.87  7.47   0.01
## 1567     6 -72.05 157.89  7.49   0.01
## 1257     6 -72.07 157.92  7.52   0.01
## 467      5 -73.50 158.26  7.86   0.00
## 257      5 -73.59 158.43  8.03   0.00
## 1467     6 -72.44 158.67  8.27   0.00
## 1247     6 -72.44 158.67  8.27   0.00
## 1267     6 -72.56 158.90  8.50   0.00
## 567      5 -73.92 159.10  8.70   0.00
## 1234567  9 -68.55 159.19  8.79   0.00
## 267      5 -74.06 159.37  8.97   0.00
## 2457     6 -72.86 159.51  9.11   0.00
## 4567     6 -73.13 160.06  9.66   0.00
## 2467     6 -73.23 160.24  9.84   0.00
## 14567    7 -71.95 160.34  9.94   0.00
##
## Term codes:
##            lam(dcon)           lam(drios) lam(I(tree_1000m^2))
##                    1                    2                    3
##      lam(tree_1000m)              p(date)              p(dras)
##                    4                    5                    6
##              p(sfrz)
##                    7
##
## Model-averaged coefficients:
## (full average)
##                      Estimate Std. Error z value Pr(>|z|)
## lam(Int)             -0.49996    0.62023   0.806  0.42020
## lam(tree_1000m)       0.93811    0.69158   1.356  0.17494
## lam(I(tree_1000m^2)) -1.00069    0.73134   1.368  0.17122
## p(Int)               -3.48506    0.98063   3.554  0.00038 ***
## p(sfrz)               3.20399    1.04117   3.077  0.00209 **
## p(date)               0.23773    0.45717   0.520  0.60306
## lam(dcon)            -0.04180    0.61825   0.068  0.94610
## lam(drios)            0.02220    0.20451   0.109  0.91358
## p(dras)              -0.01376    0.17237   0.080  0.93638
##
## (conditional average)
##                      Estimate Std. Error z value Pr(>|z|)
## lam(Int)             -0.49996    0.62023   0.806  0.42020
## lam(tree_1000m)       1.09710    0.62042   1.768  0.07701 .
## lam(I(tree_1000m^2)) -1.26470    0.58487   2.162  0.03059 *
## p(Int)               -3.48506    0.98063   3.554  0.00038 ***
## p(sfrz)               3.20399    1.04117   3.077  0.00209 **
## p(date)               0.65547    0.54995   1.192  0.23331
## lam(dcon)            -0.13776    1.11654   0.123  0.90180
## lam(drios)            0.09874    0.42251   0.234  0.81522
## p(dras)              -0.06337    0.36566   0.173  0.86240
```

```
## ---
## Signif. codes:  0 '***' 0.001 '**' 0.01 '*' 0.05 '.' 0.1 ' ' 1
```

```
ss <- match(rownames(get(sprintf("UMF.%s",spp))@y),cam.data$cdg)
prd <- predict(get(sprintf("mavg%s.%s",mod,spp)),type='state')
dtf <- data.frame(fit=prd$fit, se.fit=prd$se.fit, hunting=cam.data[ss,"hunting"], dcon=cam.data[ss,"dcon
ggplot(dtf, aes(y=dcon, x=tree_1000m,size=fit,colour=se.fit)) +
    geom_point() + ylab("Distance to conucos  (m)") + xlab("Tree cover (% in 1km buffer)") +
    labs(title=spp,size='Predicted values',colour='Prediction S.E.')
```

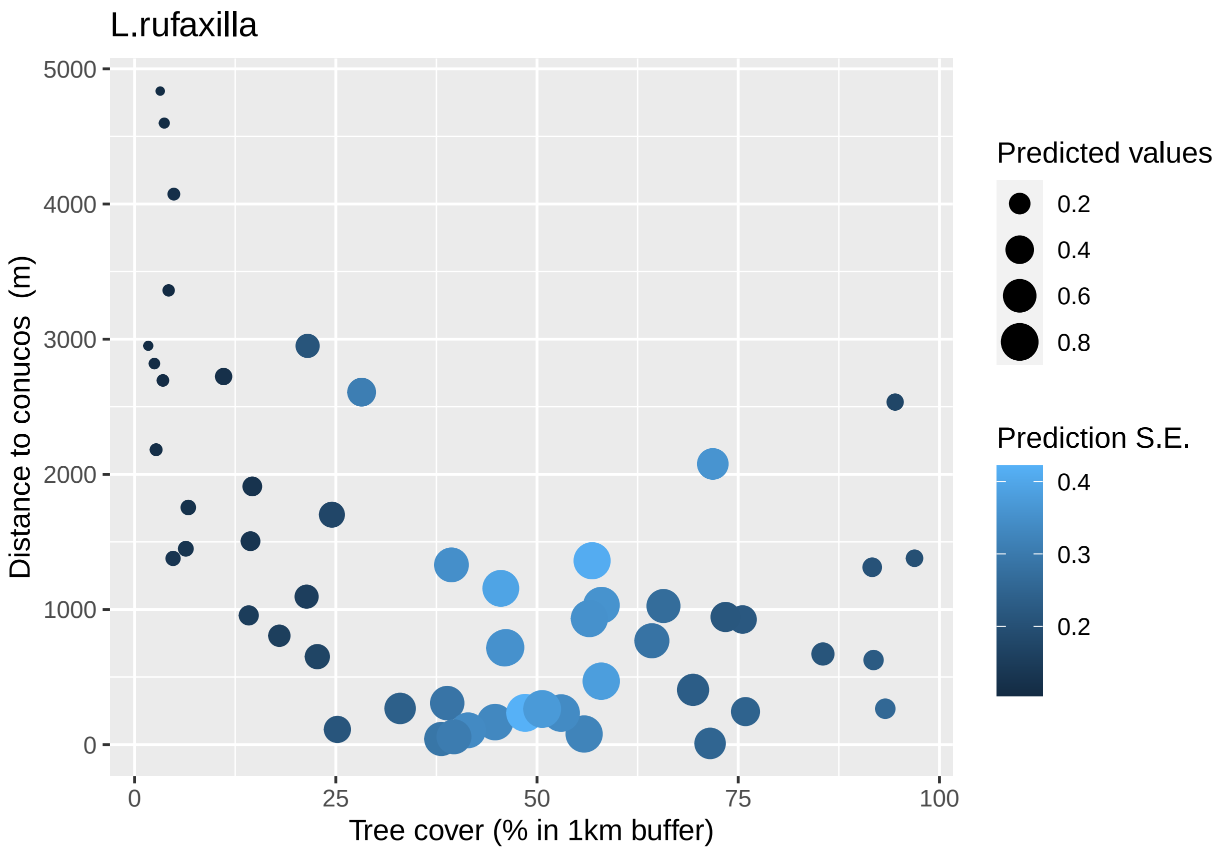

M.gouazoubira

No sign of lack of fit, c-hat values less than 1

```
spp <- "M.gouazoubira"
mod <- ifelse(spp %in% with.quad.term,"03","01")

 tbl1 %>% filter(species %in% spp) %>% select(1:5)
```

```
##          species n.detect chi.square p.value c.hat.est
## 1 M.gouazoubira       33   846.9679  0.6531 0.5205965
```

Most support for variables:

```
sw(get(sprintf("oms%s.%s",mod,spp)))
```

```
##                      lam(tree_1000m) p(sfrz) lam(dcon) lam(drios) p(date)
## Sum of weights:      1.00            0.97    0.57      0.30       0.22
## N containing models:   32              32      32        32         32
```

```
##                        p(dras)
## Sum of weights:        0.22
## N containing models:     32
```

Summary of model averaging estimates (use conditional average):

```
summary(get(sprintf("mavg%s.%s",mod,spp)))
```

```
##
## Call:
## model.avg(object = get.models(object = oms01, subset = delta <
##     10))
##
## Component model call:
## occuRN(formula = ~<24 unique rhs>, data = UMF, K = 50)
##
## Component models:
##        df logLik   AICc delta weight
## 136     5 -78.64 168.53  0.00   0.22
## 36      4 -80.02 168.86  0.32   0.19
## 1236    6 -77.95 169.69  1.15   0.12
## 1356    6 -78.64 171.06  2.53   0.06
## 1346    6 -78.64 171.07  2.54   0.06
## 346     5 -79.95 171.15  2.62   0.06
## 356     5 -79.97 171.19  2.65   0.06
## 236     5 -80.00 171.25  2.72   0.06
## 12356   7 -77.95 172.33  3.80   0.03
## 12346   7 -77.95 172.34  3.80   0.03
## 2346    6 -79.92 173.63  5.09   0.02
## 3456    6 -79.92 173.63  5.10   0.02
## 2356    6 -79.93 173.64  5.11   0.02
## 13456   7 -78.64 173.71  5.18   0.02
## 13      4 -83.13 175.08  6.54   0.01
## 123456  8 -77.95 175.10  6.57   0.01
## 3       3 -84.79 176.07  7.54   0.01
## 123     5 -82.44 176.13  7.60   0.00
## 23456   7 -79.87 176.18  7.64   0.00
## 134     5 -83.12 177.48  8.95   0.00
## 135     5 -83.13 177.51  8.98   0.00
## 34      4 -84.65 178.12  9.58   0.00
## 35      4 -84.69 178.20  9.67   0.00
## 23      4 -84.78 178.37  9.84   0.00
##
## Term codes:
##       lam(dcon)      lam(drios) lam(tree_1000m)         p(date)         p(dras)
##               1               2               3               4               5
##         p(sfrz)
##               6
##
## Model-averaged coefficients:
## (full average)
##                  Estimate Std. Error z value Pr(>|z|)
## lam(Int)         -0.33615    1.28549   0.261  0.79371
## lam(dcon)        -0.59391    0.70928   0.837  0.40240
## lam(tree_1000m)   1.13872    0.28034   4.062 4.87e-05 ***
```

```
## p(Int)          -4.62624    1.43921   3.214  0.00131 **
## p(sfrz)          2.21285    0.95209   2.324  0.02011 *
## lam(drios)       0.07215    0.19568   0.369  0.71235
## p(dras)          0.00967    0.17808   0.054  0.95669
## p(date)         -0.01368    0.18180   0.075  0.94003
##
## (conditional average)
##                 Estimate Std. Error z value Pr(>|z|)
## lam(Int)        -0.33615    1.28549   0.261  0.79371
## lam(dcon)       -1.03516    0.64813   1.597  0.11023
## lam(tree_1000m)  1.13872    0.28034   4.062 4.87e-05 ***
## p(Int)          -4.62624    1.43921   3.214  0.00131 **
## p(sfrz)          2.27752    0.88638   2.569  0.01019 *
## lam(drios)       0.24182    0.29549   0.818  0.41313
## p(dras)          0.04384    0.37718   0.116  0.90747
## p(date)         -0.06171    0.38233   0.161  0.87177
## ---
## Signif. codes:  0 '***' 0.001 '**' 0.01 '*' 0.05 '.' 0.1 ' ' 1
```

```
ss <- match(rownames(get(sprintf("UMF.%s",spp))@y),cam.data$cdg)
prd <- predict(get(sprintf("mavg%s.%s",mod,spp)),type='state')
dtf <- data.frame(fit=prd$fit, se.fit=prd$se.fit, hunting=cam.data[ss,"hunting"], dcon=cam.data[ss,"dcon
ggplot(dtf, aes(y=dcon, x=tree_1000m,size=fit,colour=se.fit)) +
    geom_point() + ylab("Distance to conucos  (m)") + xlab("Tree cover (% in 1km buffer)") +
    labs(title=spp,size='Predicted values',colour='Prediction S.E.')
```

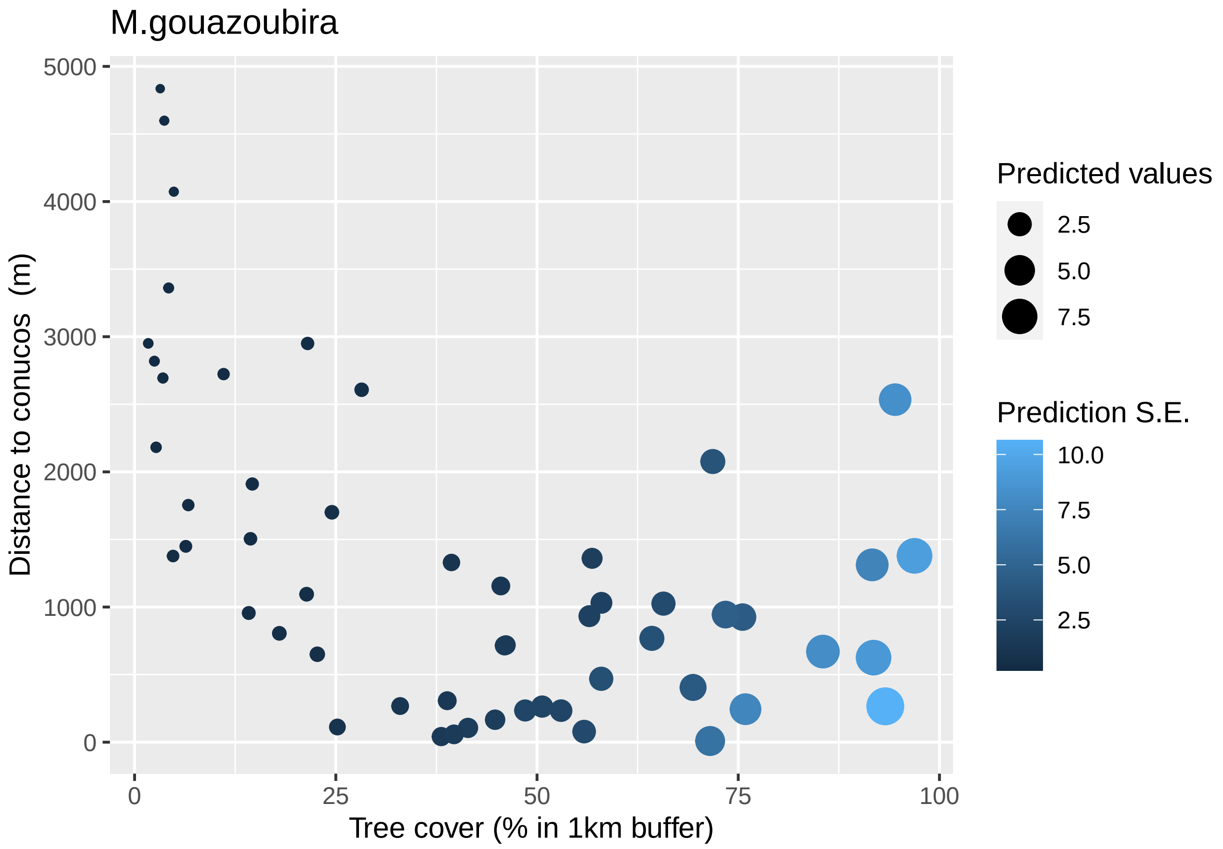

D.leporina

No sign of lack of fit, c-hat values less than 1

```
spp <- "D.leporina"
mod <- ifelse(spp %in% with.quad.term,"03","01")

 tbl1 %>% filter(species %in% spp) %>% select(1:5)
```

```
##      species n.detect chi.square p.value c.hat.est
## 1 D.leporina       66   1093.789  0.7151 0.5210335
```

Most support for variables:

```
sw(get(sprintf("oms%s.%s",mod,spp)))
```

```
##                      lam(tree_1000m) p(sfrz) lam(drios) p(dras) lam(dcon)
## Sum of weights:      1.00            0.98    0.43       0.42    0.31
## N containing models:   32              32      32         32      32
##                      p(date)
## Sum of weights:      0.29
## N containing models:   32
```

Summary of model averaging estimates (use conditional average):

```
summary(get(sprintf("mavg%s.%s",mod,spp)))
```

```
##
## Call:
## model.avg(object = get.models(object = oms01, subset = delta <
##     10))
##
## Component model call:
## occuRN(formula = ~<24 unique rhs>, data = UMF, K = 50)
##
## Component models:
##        df  logLik   AICc delta weight
## 236     5 -104.89 221.04  0.00   0.14
## 36      4 -106.13 221.09  0.05   0.14
## 356     5 -105.06 221.36  0.32   0.12
## 136     5 -105.33 221.91  0.87   0.09
## 2356    6 -104.11 222.01  0.98   0.09
## 3456    6 -104.51 222.82  1.78   0.06
## 1236    6 -104.62 223.04  2.00   0.05
## 2346    6 -104.63 223.05  2.01   0.05
## 1356    6 -104.69 223.16  2.12   0.05
## 346     5 -105.97 223.19  2.15   0.05
## 23456   7 -103.50 223.43  2.39   0.04
## 1346    6 -104.99 223.77  2.73   0.04
## 12356   7 -104.02 224.48  3.44   0.02
## 13456   7 -104.03 224.50  3.47   0.02
## 12346   7 -104.27 224.97  3.93   0.02
## 123456  8 -103.35 225.90  4.87   0.01
## 35      4 -110.04 228.89  7.86   0.00
## 13      4 -110.25 229.31  8.27   0.00
## 3       3 -111.42 229.31  8.28   0.00
## 23      4 -110.38 229.57  8.53   0.00
## 235     5 -109.30 229.84  8.80   0.00
```

```
## 135     5 -109.48 230.21  9.18   0.00
## 345     5 -109.75 230.76  9.72   0.00
## 123     5 -109.79 230.83  9.80   0.00
##
## Term codes:
##       lam(dcon)      lam(drios) lam(tree_1000m)         p(date)         p(dras)
##               1               2               3               4               5
##         p(sfrz)
##               6
##
## Model-averaged coefficients:
## (full average)
##                 Estimate Std. Error z value Pr(>|z|)
## lam(Int)        -0.54672    0.39949   1.369  0.17114
## lam(drios)      -0.14321    0.22857   0.627  0.53096
## lam(tree_1000m)  1.12224    0.23507   4.774 1.80e-06 ***
## p(Int)          -2.70254    0.63155   4.279 1.88e-05 ***
## p(sfrz)          1.73029    0.61870   2.797  0.00516 **
## p(dras)          0.20053    0.32625   0.615  0.53878
## lam(dcon)       -0.14991    0.37141   0.404  0.68648
## p(date)          0.07737    0.20506   0.377  0.70596
##
## (conditional average)
##                 Estimate Std. Error z value Pr(>|z|)
## lam(Int)         -0.5467     0.3995   1.369  0.17114
## lam(drios)       -0.3321     0.2417   1.374  0.16953
## lam(tree_1000m)   1.1222     0.2351   4.774 1.80e-06 ***
## p(Int)           -2.7025     0.6315   4.279 1.88e-05 ***
## p(sfrz)           1.7556     0.5865   2.993  0.00276 **
## p(dras)           0.4762     0.3485   1.366  0.17185
## lam(dcon)        -0.4806     0.5323   0.903  0.36658
## p(date)           0.2656     0.3072   0.865  0.38730
## ---
## Signif. codes:  0 '***' 0.001 '**' 0.01 '*' 0.05 '.' 0.1 ' ' 1
```

```r
ss <- match(rownames(get(sprintf("UMF.%s",spp))@y),cam.data$cdg)
prd <- predict(get(sprintf("mavg%s.%s",mod,spp)),type='state')
dtf <- data.frame(fit=prd$fit, se.fit=prd$se.fit, hunting=cam.data[ss,"hunting"], dcon=cam.data[ss,"dcon
ggplot(dtf, aes(y=dcon, x=tree_1000m,size=fit,colour=se.fit)) +
    geom_point() + ylab("Distance to conucos  (m)") + xlab("Tree cover (% in 1km buffer)") +
    labs(title=spp,size='Predicted values',colour='Prediction S.E.')
```

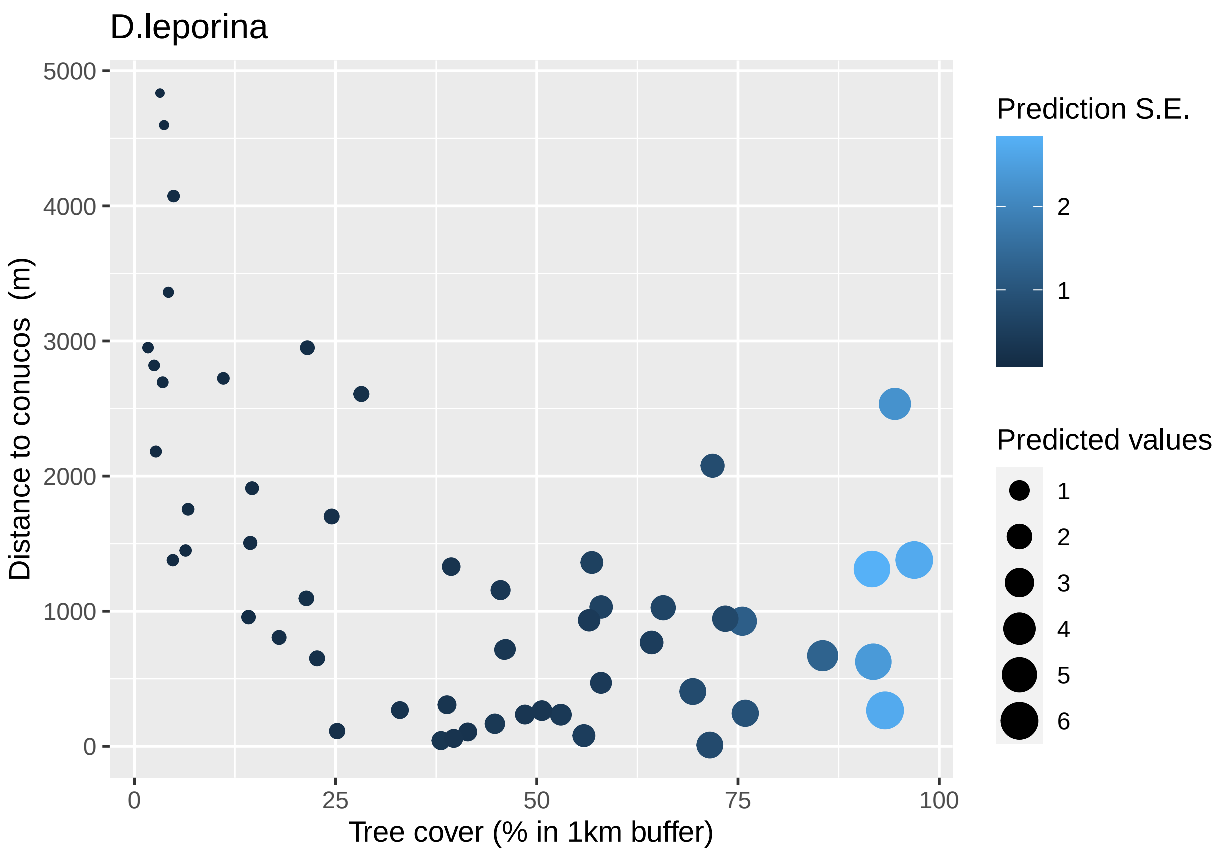

C.paca

No sign of lack of fit, c-hat values less than 1

```
spp <- "C.paca"
mod <- ifelse(spp %in% with.quad.term,"03","01")

 tbl1 %>% filter(species %in% spp) %>% select(1:5)
```

```
##   species n.detect chi.square p.value c.hat.est
## 1  C.paca       71   966.5061  0.8243 0.4413452
```

Most support for variables:

```
sw(get(sprintf("oms%s.%s",mod,spp)))
```

```
##                        p(sfrz) p(dras) lam(dcon) lam(drios) lam(tree_1000m)
## Sum of weights:        0.97    0.92    0.87      0.31       0.30
## N containing models:     32      32      32        32         32
##                        p(date)
## Sum of weights:        0.25
## N containing models:     32
```

Summary of model averaging estimates (use conditional average):

```
summary(get(sprintf("mavg%s.%s",mod,spp)))
```

```
##
## Call:
```

```
## model.avg(object = get.models(object = oms01, subset = delta <
##     10))
##
## Component model call:
## occuRN(formula = ~<27 unique rhs>, data = UMF, K = 50)
##
## Component models:
##        df  logLik   AICc delta weight
## 156     5 -114.63 240.51  0.00   0.32
## 1256    6 -114.31 242.41  1.89   0.12
## 1356    6 -114.45 242.69  2.18   0.11
## 1456    6 -114.46 242.71  2.20   0.11
## 12356   7 -114.04 244.51  4.00   0.04
## 12456   7 -114.14 244.72  4.21   0.04
## 13456   7 -114.26 244.96  4.44   0.03
## 2356    6 -115.78 245.36  4.84   0.03
## 356     5 -117.06 245.37  4.85   0.03
## 16      4 -118.38 245.58  5.07   0.03
## 256     5 -117.38 246.01  5.49   0.02
## 56      4 -118.93 246.67  6.15   0.01
## 123456  8 -113.85 246.89  6.38   0.01
## 146     5 -117.94 247.13  6.62   0.01
## 3456    6 -116.75 247.28  6.76   0.01
## 126     5 -118.07 247.40  6.88   0.01
## 15      4 -119.31 247.43  6.92   0.01
## 23456   7 -115.54 247.51  6.99   0.01
## 136     5 -118.22 247.70  7.18   0.01
## 2456    6 -117.22 248.23  7.72   0.01
## 456     5 -118.69 248.63  8.12   0.01
## 1246    6 -117.64 249.07  8.56   0.00
## 1346    6 -117.74 249.27  8.76   0.00
## 145     5 -119.01 249.28  8.76   0.00
## 135     5 -119.06 249.38  8.86   0.00
## 125     5 -119.09 249.43  8.92   0.00
## 1236    6 -117.83 249.44  8.93   0.00
##
## Term codes:
##       lam(dcon)      lam(drios) lam(tree_1000m)         p(date)         p(dras)
##               1               2               3               4               5
##         p(sfrz)
##               6
##
## Model-averaged coefficients:
## (full average)
##                 Estimate Std. Error z value Pr(>|z|)
## lam(Int)        -0.60703    0.39003   1.556 0.119623
## lam(dcon)       -1.09246    0.65289   1.673 0.094277 .
## p(Int)          -2.32967    0.61499   3.788 0.000152 ***
## p(dras)          0.82161    0.39147   2.099 0.035836 *
## p(sfrz)          1.71387    0.64584   2.654 0.007961 **
## lam(drios)      -0.08817    0.21720   0.406 0.684776
## lam(tree_1000m)  0.06292    0.16725   0.376 0.706762
## p(date)         -0.05039    0.18218   0.277 0.782084
##
```

```
## (conditional average)
##                   Estimate Std. Error z value Pr(>|z|)
## lam(Int)           -0.6070     0.3900   1.556 0.119623
## lam(dcon)          -1.2476     0.5415   2.304 0.021224 *
## p(Int)             -2.3297     0.6150   3.788 0.000152 ***
## p(dras)             0.8816     0.3340   2.640 0.008293 **
## p(sfrz)             1.7515     0.6003   2.918 0.003524 **
## lam(drios)         -0.2880     0.3107   0.927 0.353955
## lam(tree_1000m)     0.2129     0.2504   0.850 0.395326
## p(date)            -0.2019     0.3200   0.631 0.528048
## ---
## Signif. codes:  0 '***' 0.001 '**' 0.01 '*' 0.05 '.' 0.1 ' ' 1
```

```
ss <- match(rownames(get(sprintf("UMF.%s",spp))@y),cam.data$cdg)
prd <- predict(get(sprintf("mavg%s.%s",mod,spp)),type='state')
dtf <- data.frame(fit=prd$fit, se.fit=prd$se.fit, hunting=cam.data[ss,"hunting"], dcon=cam.data[ss,"dcon
ggplot(dtf, aes(y=dcon, x=tree_1000m,size=fit,colour=se.fit)) +
    geom_point() + ylab("Distance to conucos  (m)") + xlab("Tree cover (% in 1km buffer)") +
    labs(title=spp,size='Predicted values',colour='Prediction S.E.')
```

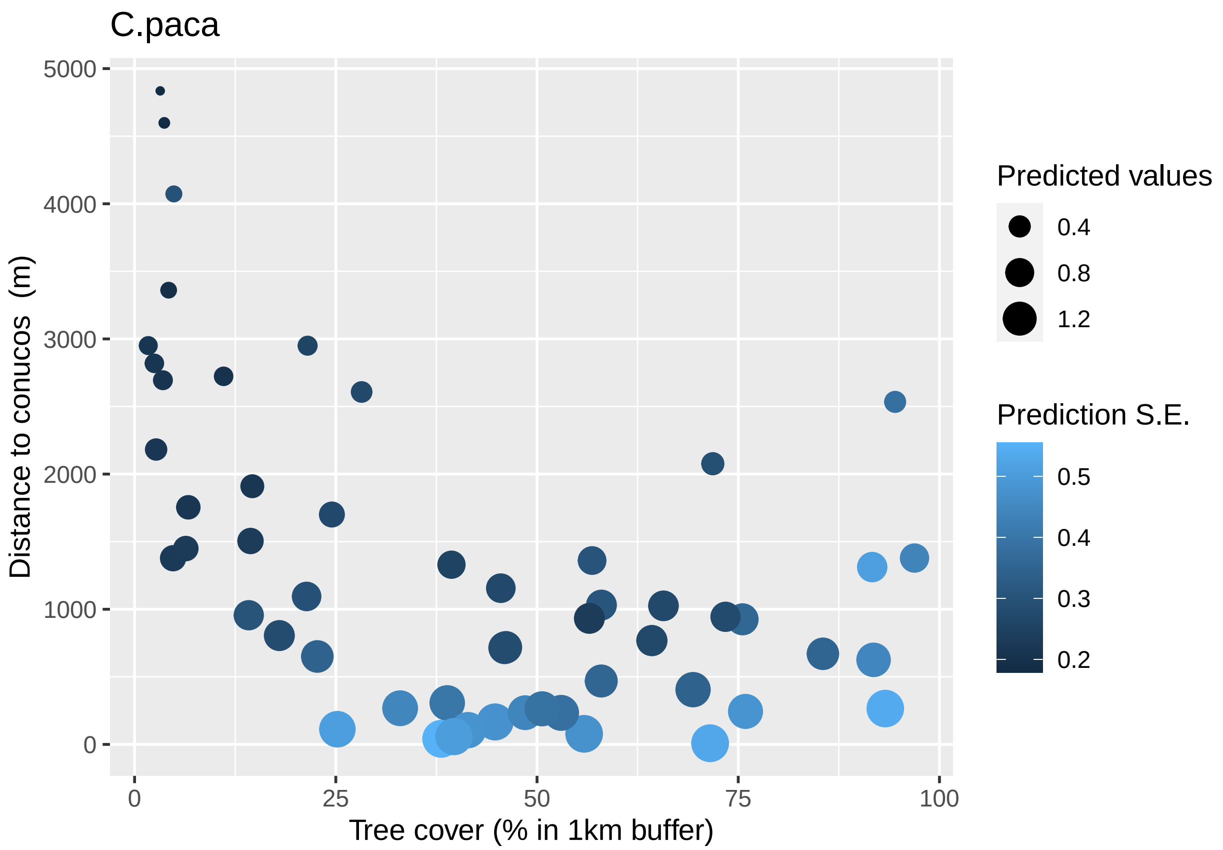

Combining results from all species

Summary of support for all variables

```
tbl1 %>% filter(n.detect>=10) %>% arrange(n.detect) %>% pull(species) -> spps
```

```r
ccs <- sws <- data.frame()
for (spp in spps) {
##  print(spp)
  if (spp %in% with.quad.term) {
    prb <- sw(get(sprintf("oms03.%s",spp)))
    mavg <- get(sprintf("mavg03.%s",spp))
  } else {
    prb <- sw(get(sprintf("oms01.%s",spp)))
    mavg <- get(sprintf("mavg01.%s",spp))
  }
  sws <- rbind(sws,data.frame(spp,var=names(prb),w=prb))

  ccs <- rbind(ccs,data.frame(spp, coef(mavg,full=F),confint(mavg,full=F)))
}
```

```r
dts <- dcast(sws,spp~var,value.var="w")
dts %>% select(spp,`p(sfrz)`, `p(dras)`, `p(date)`, `lam(tree_1000m)`, `lam(I(tree_1000m^2))`, `lam(dco
```

```
##                spp   p(sfrz)   p(dras)   p(date) lam(tree_1000m)
## 1    D.imperfecta 0.4494450 0.9551473 0.2276180       0.3753545
## 2          P.onca 0.2341765 0.2544872 0.2387225       0.6751411
## 3    M.tridactyla 0.4790563 0.6020121 0.2275080       0.3512140
## 4      L.pardalis 0.2445099 0.2450353 0.2258898       0.3461923
## 5       E.barbara 0.2435057 0.2237059 0.2276751       0.8690906
## 6  D.novemcinctus 0.2246002 0.2282753 0.8502132       0.4065220
## 7     M.americana 0.9984840 0.2349651 0.8772999       0.9797946
## 8         T.major 0.2316186 0.2917358 0.2374788       0.9744634
## 9         C.thous 0.4211804 0.2592308 0.2312622       0.5421025
## 10     D.kappleri 0.6467966 0.4913864 0.4608847       0.9996079
## 11      C.alector 0.7286448 0.7101610 0.2350957       0.9812248
## 12    L.rufaxilla 0.9951120 0.2203712 0.3652459       0.8534100
## 13  M.gouazoubira 0.9661162 0.2230601 0.2241691       0.9991263
## 14     D.leporina 0.9811035 0.4212674 0.2939650       0.9999112
## 15         C.paca 0.9683059 0.9240713 0.2538367       0.3020965
##    lam(I(tree_1000m^2)) lam(dcon) lam(drios)
## 1                    NA 0.2366762  0.2355505
## 2                    NA 0.2647327  0.3024281
## 3                    NA 0.3797467  0.8933935
## 4                    NA 0.2588223  0.2620176
## 5             0.2335889 0.8431175  0.2387931
## 6                    NA 0.3182860  0.2283743
## 7                    NA 0.2442779  0.2507425
## 8                    NA 0.2529395  0.2418776
## 9                    NA 0.3120142  0.3084007
## 10                   NA 0.4706912  0.4476599
## 11                   NA 0.6395410  0.2310792
## 12            0.7865129 0.3058415  0.2295178
## 13                   NA 0.5746615  0.3012296
## 14                   NA 0.3131305  0.4317585
## 15                   NA 0.8686392  0.3114654
```

Hypothesis test: effect of conucos

```
ccs  %>% filter(grepl('dcon',rownames(ccs))) %>% dplyr::arrange(coef.mavg..full...F.) -> ss
##ccs  %>% filter(grepl('drios',rownames(ccs))) %>% dplyr::arrange(coef.mavg..full...F.) -> ss
##ccs  %>% filter(grepl('tree_1000m',rownames(ccs))) %>% dplyr::arrange(coef.mavg..full...F.) -> ss
par(mar=c(4,8,3,1))
plot(ss$coef.mavg.,1:nrow(ss),xlim=c(-3.8,3.8), pch=19,xlab=expression(hat(beta)),ylab='',axes=F,main='
segments(ss$X2.5..,   1:nrow(ss),   ss$X97.5..,1:nrow(ss))
axis(1)
axis(2,1:nrow(ss),ss$spp,las=2,font=3)
box()
abline(v=0,lty=2,lwd=2,col=2)
```

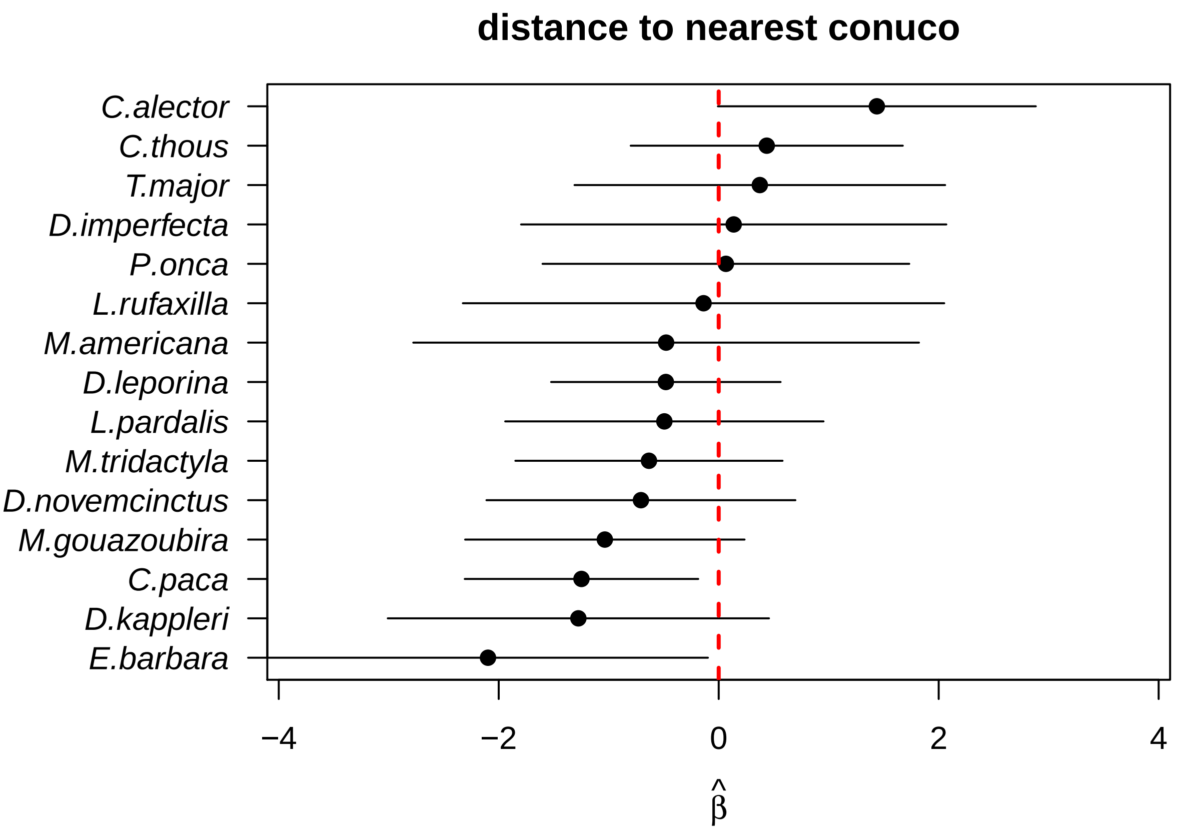


```
# dev.copy(png,file='Fig-coefficient-distance.png')
# dev.off()
```

Predicted abundance in hunting sites

For all species reported as hunted (need to reorder plot, maybe exclude species with large uncertainty in prediction).

```
Hv <- c('C.paca'=6.336,'C.alector'=4.630, 'D.leporina'=2.681, 'T.terrestris'=2.681,'T.major'=1.949, 'M.
mtz <- data.frame()

for (k in spps[spps %in% names(Hv)]) {
  if (spp %in% with.quad.term) {
      mtz <- rbind(mtz,data.frame(species=k,abundance=predict(get(sprintf("mavg03.%s",k)),type='state')
```

```r
    } else {
      mtz <- rbind(mtz,data.frame(species=k,abundance=predict(get(sprintf("mavg01.%s",k)),type='state')
    }
}

#mtz$hunting <- ifelse(mtz$caza==1,'yes','no')
#mtz$hunting <- ifelse(mtz$caza>0,'yes','no')

# text_Hv1 <- textGrob(sprintf("(%s)",Hv[1]), gp=gpar(fontsize=7))
# text_Hv2 <- textGrob(sprintf("(%s)",Hv[2]), gp=gpar(fontsize=7))
# text_Hv3 <- textGrob(sprintf("(%s)",Hv[3]), gp=gpar(fontsize=7))
# text_Hv4 <- textGrob(sprintf("(%s)",Hv[4]), gp=gpar(fontsize=7))
# text_Hv5 <- textGrob(sprintf("(%s)",Hv[5]), gp=gpar(fontsize=7))
# text_Hv6 <- textGrob(sprintf("(%s)",Hv[6]), gp=gpar(fontsize=7))
# text_Hv8 <- textGrob(sprintf("(%s)",Hv[8]), gp=gpar(fontsize=7))

# grouped boxplot
ggplot(mtz %>% filter(), aes(x=species, y=abundance, fill=hunting)) +
    geom_boxplot(notch=F) + # or notch=T
    labs(title="Model prediction of abundance at sites with and without hunting") +
    labs(y=expression( hat(lambda)), x="",caption="Species (Hv values)") +
    theme(axis.text.x = element_text( size = 7, hjust = .5, vjust=.5, face = "italic"),
    plot.margin = unit(c(1,1,2,1), "lines")) +
    coord_cartesian(clip="off")
```

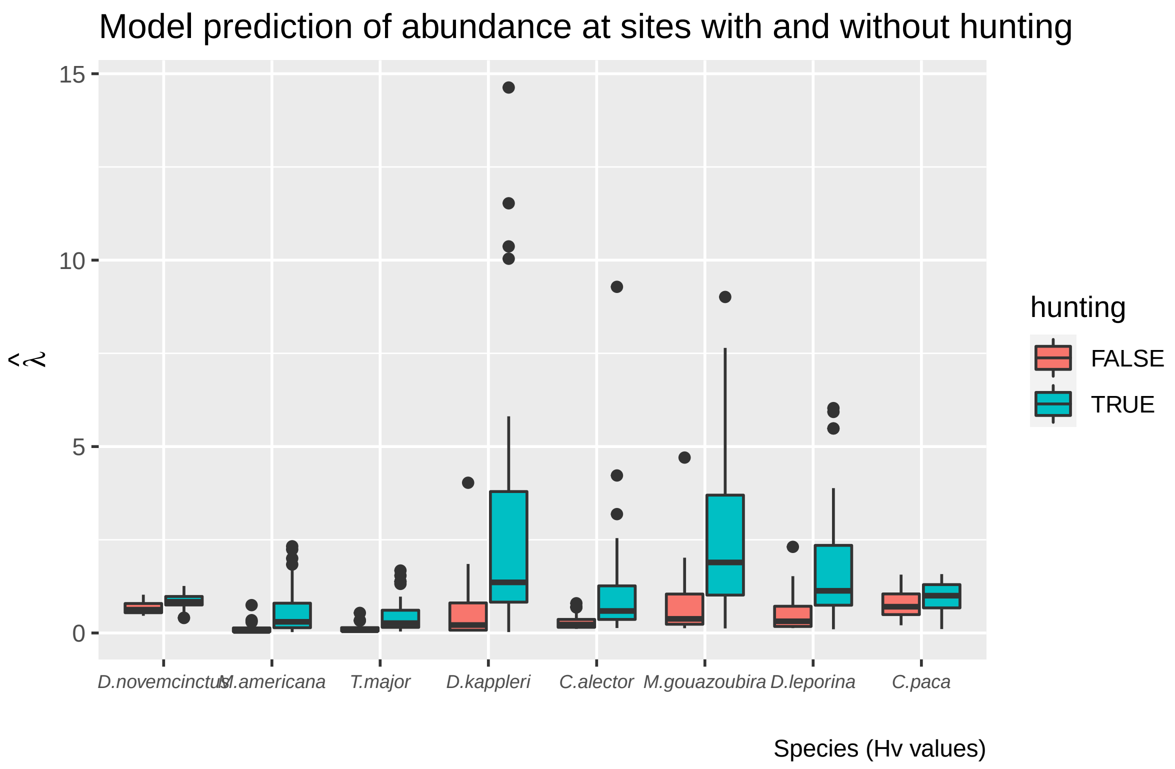

```r
  # P +  annotation_custom(text_Hv1,xmin=1,xmax=1,ymin=-0.5,ymax=-0.5) +
  # annotation_custom(text_Hv2,xmin=2,xmax=2,ymin=-0.5,ymax=-0.5) +
  #  annotation_custom(text_Hv3,xmin=3,xmax=4,ymin=-0.5,ymax=-0.5) +
  #   annotation_custom(text_Hv5,xmin=5,xmax=5,ymin=-0.5,ymax=-0.5) +
  #   annotation_custom(text_Hv6,xmin=6,xmax=7,ymin=-0.5,ymax=-0.5) +
  #   annotation_custom(text_Hv8,xmin=8,xmax=9,ymin=-0.5,ymax=-0.5)


# vjust = c(.3,.3,.3,.7,.3,.7,.3,.3,.3,.3)
## ggsave("Fig-abundance-hunting.png",width=8,height=5)
# ggsave("Fig-abundance-hunting-with-notches.png",width=8,height=5)
```

Exclude M.tridactyla and E.barbara (predictions are unrealistic, too high).

```r
exc <- c('M.tridactyla','E.barbara')

mtz <- data.frame()

for (k in spps[!(spps %in% names(Hv)) & !(spps %in% exc)]) {
  if (spp %in% c('C.alector','L.rufaxilla','T.tetradactyla')) {
      mtz <- rbind(mtz,data.frame(species=k,abundance=predict(get(sprintf("mavg03.%s",k)),type='state')$
    } else {
      mtz <- rbind(mtz,data.frame(species=k,abundance=predict(get(sprintf("mavg01.%s",k)),type='state')$
    }
}

# grouped boxplot
ggplot(mtz %>% filter(), aes(x=species, y=abundance, fill=hunting)) +
    geom_boxplot(notch=F) + # or notch=T
    labs(title="Model prediction of abundance at sites with and without hunting") +
    labs(y=expression( hat(lambda)), x="") +
theme(axis.text.x = element_text( size = 7, hjust = .5, vjust=.5, face = "italic"))
```

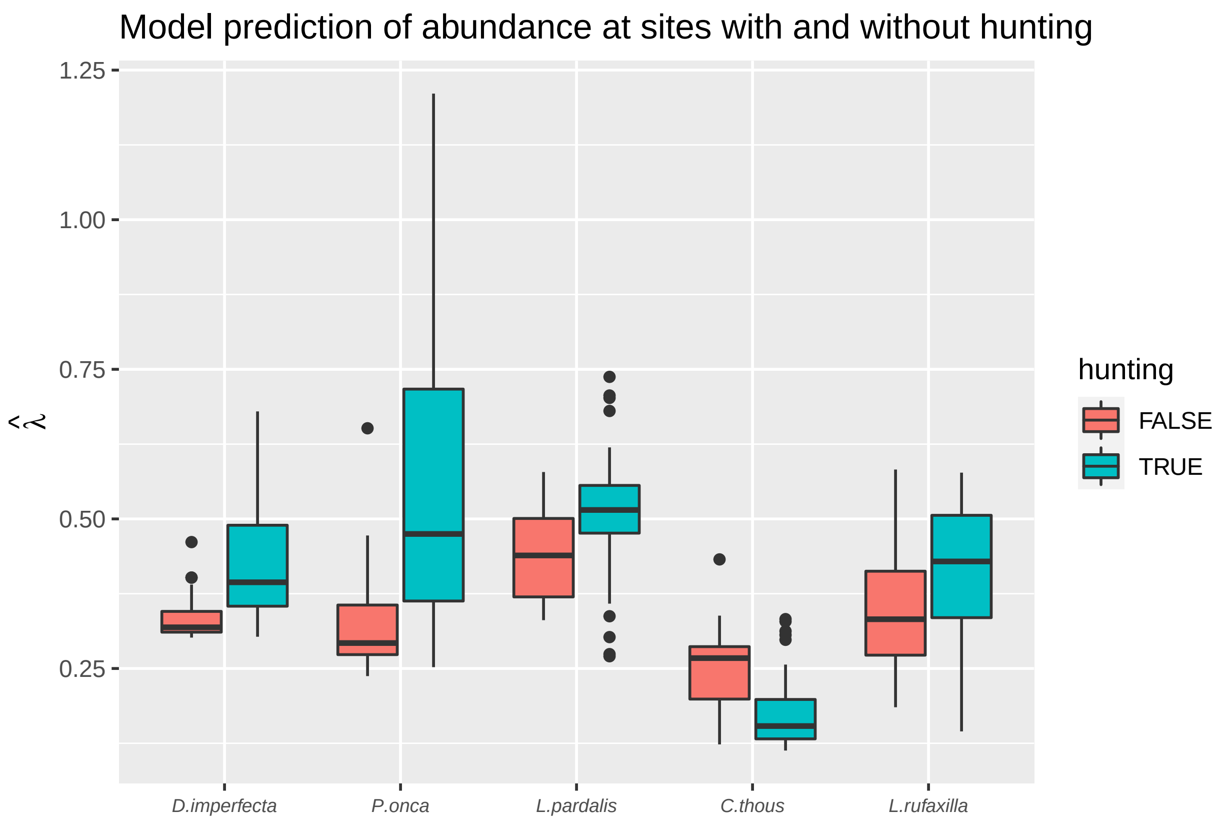


```
# ggsave("Fig-abundance-hunting-not-reported.png",width=8,height=5)
```

Location of hunting sites

Logistic regression (binomial glm) for reported hunting sites (hunting vs. no hunting) vs. habitat and conuco variables:

```
mdl <- glm(hunting~tree_1000m+drios+dcom+dcon, data=cam.data,family=binomial)
summary(mdl)
```

```
##
## Call:
## glm(formula = hunting ~ tree_1000m + drios + dcom + dcon, family = binomial,
##     data = cam.data)
##
## Deviance Residuals:
##     Min       1Q   Median       3Q      Max
## -2.4240  -0.8136   0.3159   0.8162   1.7830
##
## Coefficients:
##               Estimate Std. Error z value Pr(>|z|)
## (Intercept) -2.5180962  1.0110348  -2.491   0.0128 *
## tree_1000m   0.0341940  0.0149561   2.286   0.0222 *
## drios        0.0010746  0.0005327   2.017   0.0437 *
## dcom         0.0002970  0.0001640   1.811   0.0702 .
## dcon        -0.0005164  0.0003071  -1.682   0.0926 .
## ---
```

```
## Signif. codes:  0 '***' 0.001 '**' 0.01 '*' 0.05 '.' 0.1 ' ' 1
##
## (Dispersion parameter for binomial family taken to be 1)
##
##     Null deviance: 82.108  on 59  degrees of freedom
## Residual deviance: 58.950  on 55  degrees of freedom
## AIC: 68.95
##
## Number of Fisher Scoring iterations: 5
```
