# Supplementary material for: Shifting cultivation and hunting across the savanna-forest mosaic in the Gran Sabana, Venezuela: facing changes
Source: PeerJ. 2021 Jun 17;9:e11612. doi: 10.7717/peerj.11612 (PMC8214850; doi:10.7717/peerj.11612)
Supplement: Supplemental Information 3 [file peerj-09-11612-s003.pdf]

Figure S1. Wildlife recorded with camera traps in the study area.

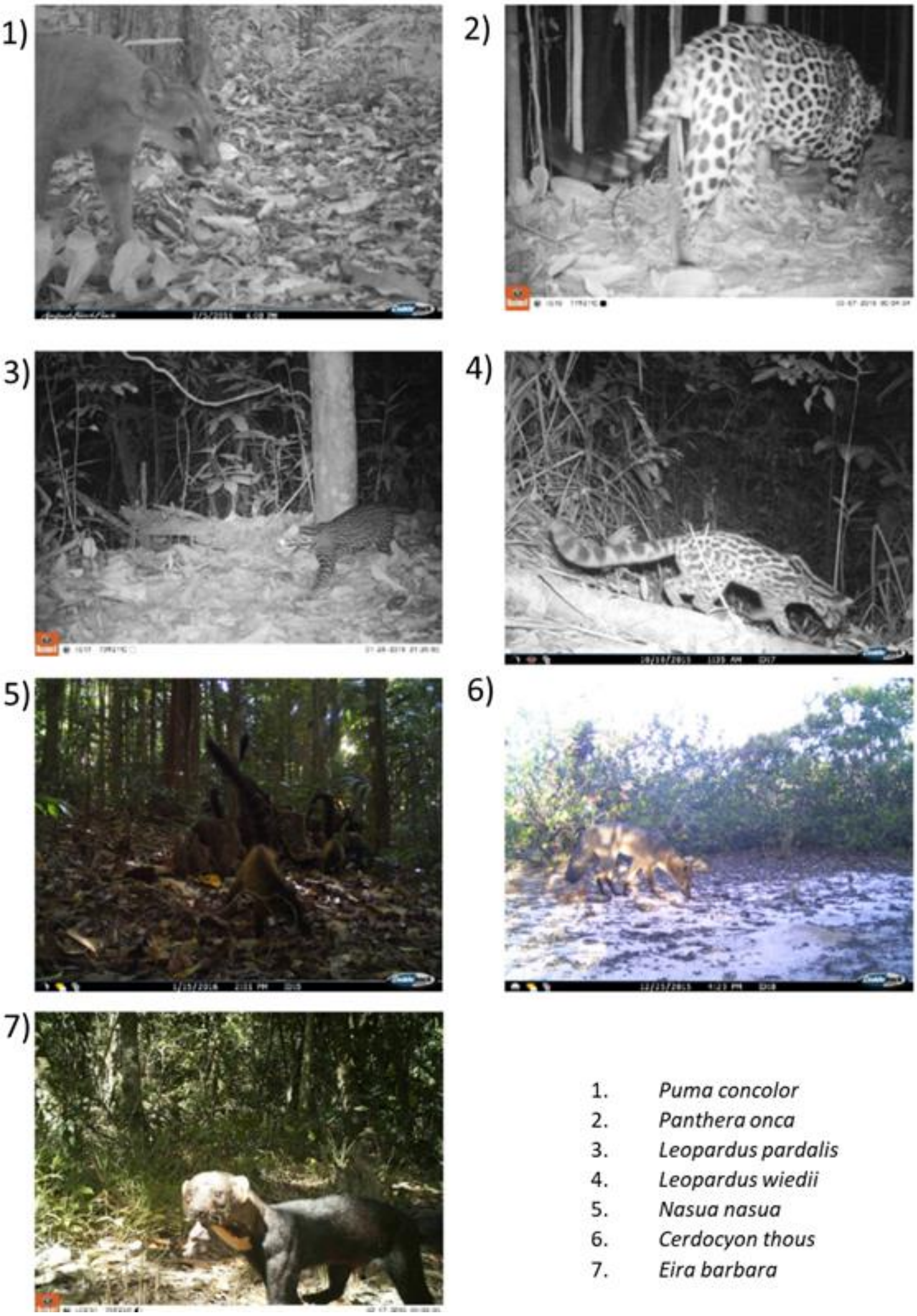

1. *Puma concolor*
2. *Panthera onca*
3. *Leopardus pardalis*
4. *Leopardus wiedii*
5. *Nasua nasua*
6. *Cerdocyon thous*
7. *Eira barbara*

Figure S1. Wildlife recorded with camera traps in the study area.

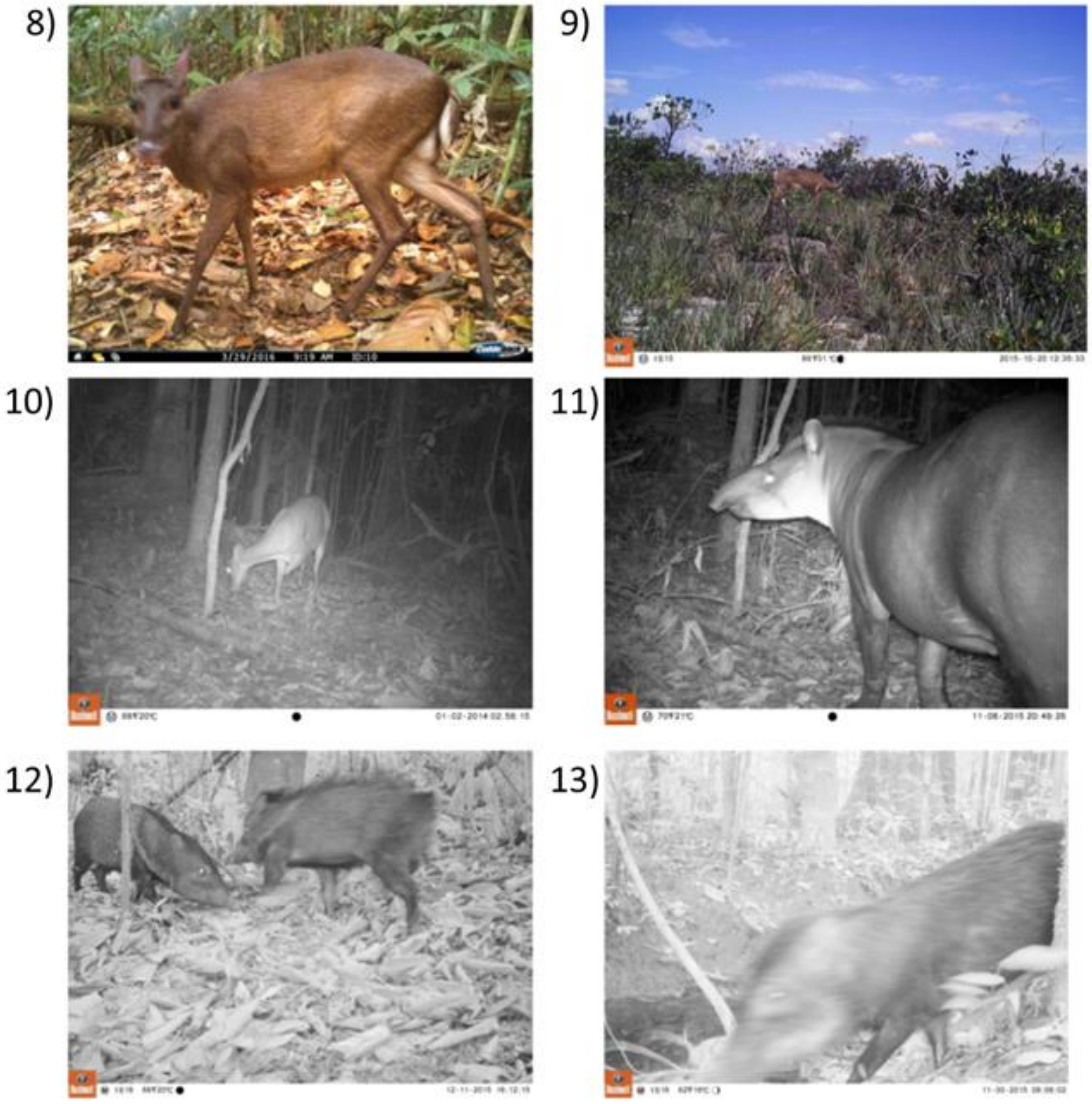

8. *Mazama gouazoubira*
9. *Odocoileus virginianus*
10. *Mazama americana*
11. *Tapirus terrestris*
12. *Pecari tajacu*
13. *Tayassu pecari*

Figure S1. Wildlife recorded with camera traps in the study area.

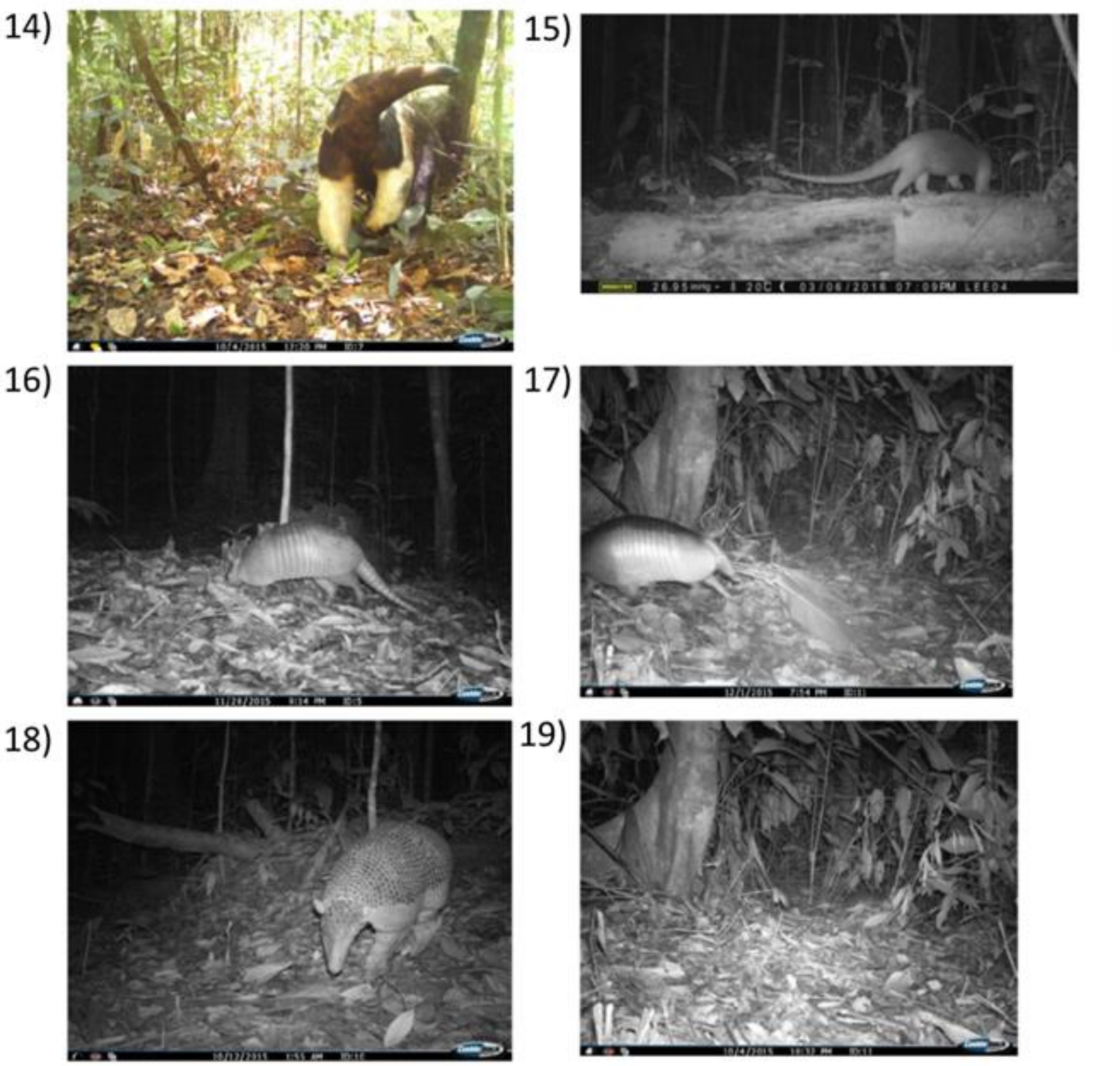

14. *Myrmecophaga tridactyla*
15. *Tamandua tetradactyla*
16. *Dasypus kappleri*
17. *Dasypus novemcinctus*
18. *Priodontes maximus*
19. *Cabassous unicinctus*
