# Supplementary material for: Shifting cultivation and hunting across the savanna-forest mosaic in the Gran Sabana, Venezuela: facing changes
Source: PeerJ. 2021 Jun 17;9:e11612. doi: 10.7717/peerj.11612 (PMC8214850; doi:10.7717/peerj.11612)
Supplement: Supplemental Information 5 [file peerj-09-11612-s005.pdf]

Encuesta de uso de fauna: proyecto "Mamiferos de la Gran Sabna"

Entrevista codigo # ______ Fecha ______

Localidad ______ Punto GPS ______

Señor(a). Mi nombre es Izabela Stachowicz, soy estudiante de doctorado en ecología en el Instituto Venezolano de Investigaciones Científicas, en donde realizo el estudio sobre la fauna de la Gran Sabana y los factores que la impactan como el fuego, cacería y deforestación. Nos gustaría nos conceda parte de su valioso tiempo para responder unas preguntas que nos permita comprender mejor la problemática socio-ambiental sobre la actividad de cacería en su comunidad. Es importante que sepa que sus respuestas son totalmente confidenciales y de uso exclusivo para la investigación. La participación en este estudio es completamente voluntaria. Si decide no participar, no habrá consecuencias negativas. Tenga en cuenta que si decide participar, puede dejar de participar en cualquier momento y puede decidir no responder a ninguna pregunta específica. De antemano agradecemos su disposición y colaboración en esta investigación.

Persona que da la informacion:

Criollo ______ Indigena ______ Edad ______ Sexo ______

Ocupación ______

¿Cuántas personas conforman su núcleo familiar? ______

¿Usted caza? ______

Metodo de caza

______

¿Que actividades realiza para conseguir alimentos? regalado ______

comprado ______ cazado ______ otra ______

¿Que animales se caza en la zona? ______

¿Que animales de esta lista se caza? LISTA

¿Cuál es la zona donde se consiguen más animales?

¿Tiene la zona preferible? si no como se llama? ______

bosque ______ sabana ______ rio ______ mixto ______

otro ______

¿En que época del año se caza más animales? ______

¿Que otra actividad realiza?

Agricultura ______ Pesca ______ Ganaderia ______ Comercio ______

Turismo ______ Otra ______

Contacto: Carretera Panamericana, Km. 11, Altos de Pipe Teléfonos:Izabela Stachowicz, 04123639334

Encuesta de uso de fauna: proyecto "Mamiferos de la Gran Sabna"

Entrevista # [ ] Fecha [ ]

¿Alguien más en su famila caza? si [ ] no [ ]

¿Qué animales y cuántos de c/u cazó usted? semana pasada........... el mes pasado.........

¿Qué animales y cuántos de c/a cazó usted?

	1-2 por sem	semanal	1-2 por mes	mensual	otra
1.					
2.					
3.					
4.					
5.					
6.					
...					

¿Para que utilza los animales cazados? para comer [ ]

venta de carne venta de cuero [ ] otro [ ]

¿Cuales animales son mas escasos hoy que antes?

[ ]

DATOS DE LA ULTIMA FECHA DE CAZA No caza [ ]

¿Cuándo fue la última vez que fue a cazar? [ ]

¿Donde cazó? (distancia o tiempo desde su sitio de vivienda) [ ]

¿Cuánto tiempo estuve cazando? [ ]

¿Qué presas y cuantas trajo?

[ ]

Gracias por brindarnos su valioso tiempo y proveernos de tan importante información para este proyecto.

Contacto: Carretera Panamericana, Km. 11, Altos de Pipe Teléfonos:Izabela Stachowicz, 04123639334
