# Supplementary material for: Shifting cultivation and hunting across the savanna-forest mosaic in the Gran Sabana, Venezuela: facing changes
Source: PeerJ. 2021 Jun 17;9:e11612. doi: 10.7717/peerj.11612 (PMC8214850; doi:10.7717/peerj.11612)
Supplement: Supplemental Information 6 [file peerj-09-11612-s006.pdf]

Original version in Spanish provided in Spanish in the Gran Sabana, Venezuela.

Fauna use survey: "Mamiferos de la Gran Sabna" project

Interview code # Date

Location GPS point

Mr/Mrs. My name is Izabela Stachowicz, I am a doctoral student in ecology at the Venezuelan Institute of Research Scientific, where I carry out a study on the fauna of the Gran Sabana and the factors that impact it such as fire, hunting and deforestation. We would like you to give us some of your valuable time to answer some questions that will allow us to better understand the socio-environmental problem about hunting activity in his community. It is important that you know that your answers are totally confidential and for the exclusive use of research. Participation in this study is completely voluntary. If you decide do not participate, there will be no negative consequences. Please note that if you decide to participate, you can stop participating at any time and you can decide not to answer any specific questions. We thank you in advance for your willingness and collaboration in this research.

Person giving the information:

Creole Indigenous Age Sex

Occupation

How many people make up your family nucleus?

Do you hunt?
Hunting method

What activities do you do to get food? given

bought hunted other

What animals are hunted in the area?

What animals on this list are hunted? LIST
What is the area where you get the most animals?
Do you have the preferable area? Yes do not as it is called?

Forest bed sheet River mixed
other

At what time of the year do you hunt the most animals?

What other activity do you do?

farming Fishing Cattle raising Trade

tourism Other

Fauna use survey: "Mamiferos de la Gran Sabna" project

Interview # Date

Does anyone else in your family hunt? Yes do not

What animals and how many of each did you hunt? last week .... last month ....

What animals and how many of c / a did you hunt?

1-2 per week 1-2 per month monthly other

1.
two.
3.
Four.
5.
6.
...

What do you use hunted animals for? to eat

sale of meat leather sale other

What animals are scarcer today than before?

DATA OF THE LAST HUNTING DATE No hunting

When was the last time you went hunting?

Where did he hunt? (distance or time from your place of residence)

How long have I been hunting?

What prey and how many did it bring?

Thank you for giving us your valuable time and providing us with such important information for this project.
